# HOO^•^ as the Chain Carrier for the Autocatalytic Photooxidation of Benzylic Alcohols

**DOI:** 10.3390/molecules29143429

**Published:** 2024-07-22

**Authors:** Xiao-Yu Wang, Huan-E Lao, Hao-Yue Zhang, Yi Wang, Qing Zhang, Jie-Qing Wu, Yu-Feng Li, Hong-Jun Zhu, Jian-You Mao, Yi Pan

**Affiliations:** 1 School of Chemistry and Molecular Engineering, Nanjing Tech University, Nanjing 211816, China; wangxiaoyu@njtech.edu.cn (X.-Y.W.); laohuane@163.com (H.-E.L.); 202161205130@njtech.edu.cn (H.-Y.Z.); 202262105017@njtech.edu.cn (Q.Z.); 202262105004@njtech.edu.en (J.-Q.W.); 2 School of Chemistry and Chemical Engineering, Nanjing University, Nanjing 210023, China; yiwang@nju.edu.cn (Y.W.); yipan@nju.edu.cn (Y.P.)

**Keywords:** hydroperoxyl radical, autocatalysis, photooxidation, benzylic alcohols, aromatic acids, aromatic ketones

## Abstract

The oxidation of benzylic alcohols is an important transformation in modern organic synthesis. A plethora of photoredox protocols have been developed to achieve the aerobic oxidation of alcohols into carbonyls. Recently, several groups described that ultraviolet (UV) or purple light can initiate the aerobic oxidation of benzylic alcohols in the absence of an external catalyst, and depicted different mechanisms involving the photoinduction of ^•^O_2_^−^ as a critical reactive oxygen species (ROS). However, based on comprehensive mechanistic investigations, including control experiments, radical quenching experiments, EPR studies, UV–vis spectroscopy, kinetics studies, and density functional theory calculations (DFT), we elucidate here that HOO^•^, which is released via the H_2_O_2_ elimination of α-hydroxyl peroxyl radicals [ArCR(OH)OO^•^], serves as the real chain carrier for the autocatalytic photooxidation of benzylic alcohols. The mechanistic ambiguities depicted in the precedent literature are clarified, in terms of the crucial ROS and its evolution, the rate-limiting step, and the primary radical cascade. This work highlights the necessity of stricter mechanistic analyses on UV-driven oxidative reactions that involve aldehydes’ (or ketones) generation.

## 1. Introduction

The oxidation of alcohols is a fundamental transformation in modern organic synthesis [1,2]. In this field, catalytic oxygen oxidation that uses oxygen or air as the clean oxidant, with the promise to enhance the reaction economics and minimize the potential environmental impact, has attracted growing interest. One of the underlying topics in oxygen oxidation reactions is the utilization of effective catalysts to initiate the inert triplet oxygen (^3^O_2_) into reactive oxygen species (ROS) for reaction triggering [3]. Over the past few decades, a plethora of O_2_-excitation-based methods have been established for benzylic alcohols’ oxidation, piloted by photo- and electro-chemical strategies [4,5,6,7,8,9,10,11,12,13,14,15,16,17,18,19,20,21]. Among them, the rapidly growing photoredox catalysis holds the greatest promise for achieving new industrial breakthroughs. However, most photocatalytic processes still rely on elaborately designed photocatalysts, including costly and toxic transition metal complexes, structurally intricate and expensive organic dyes, as well as semiconductor materials capable of inducing the photodegradation of organic compounds. Thus, these inherent flaws of photocatalysts hamper their industrial application.

Recently, several external catalyst-free methods have been presented, using UV light for the direct oxidation of alcohols [22,23,24]. For example, Vandana and coworkers described that the exposure of a dimethyl sulfoxide (DMSO) solution of benzylic alcohols in presence of oxygen under UV light with a 254 nm wavelength led to the selective formation of arylaldehydes. Liu and coworkers further discovered that long-wavelength ultraviolet light (UVA, 365–395 nm) irradiation could more efficiently promote the aerobic oxidation of benzylic alcohols in acetone solution. In Liu’s work, singlet oxygen (^1^O_2_) was claimed as the initiating ROS evolved from ^3^O_2_ via energy transfer (ET) from UV light. They also suggested that superoxide anion (^•^O_2_^−^), which was derived via single-electron transfer (SET) from the benzylic methylene group to ^1^O_2_, was another key ROS for the overall transformation.

Considering our long-term interest in green oxidation [25,26], we describe here a more effective aerobic oxidation of benzylic alcohols in the presence of 1,2-dichloroethane under 365 nm LED irradiation. Based on further comprehensive mechanistic studies, we also clarify the unreasonable and ambiguous parts of the reaction mechanism described in the precedent literature. As our investigations demonstrate, the UVA response of aryl aldehydes and ketones induces the reaction to exhibit an entire autocatalytic feature and only hydroxyl peroxide (HOO^•^), proven to be the essential chain carrier in the radical cascade. This work underscores that the UVA sensitization of aromatic aldehydes and ketones should be taken into account for strict mechanistic analyses on UVA-driven oxidative reactions that involve their generation. 

## 2. Results and Discussions

We initiated our studies using benzyl alcohol (**1a**, 1 mmol) as the template substrate for catalyst exploration and condition optimization (Table 1). When various solvents were examined in the presence of O_2_ (1 atm) under 365 nm light (25 w) irradiation, we found that both DMSO and acetone were inferior solvents compared to acetonitrile (MeCN) and 1,2-dichloroethane (DCE) (entries 1–7). In particular, DCE emerged as the most efficacious, leading to almost a stoichiometric yield of **1c** (98% assay yield) within 8 h (entry 4). Upon tuning the wavelength to 400 and 455 nm, the reactions failed to take place in DCE, demonstrating a conspicuous reaction initiation dependence on the light wavelength (entries 8 and 9). *p*-Toluenesulfonic acid (*p*-TsOH, 10 mol%) and methanesulfonic acid (MsOH 10 mol%), which have been identified as initiating photocatalysts for ^1^O_2_ generation [25,27], were also examined. However, they both resulted in an obvious lowered conversion under 365 or 400 nm light (entries 9–12). Hereto, the optimum conditions for this catalyst-free oxidation were accomplished by using DCE solvent and 365 nm light irradiation.

**Figure 1:**
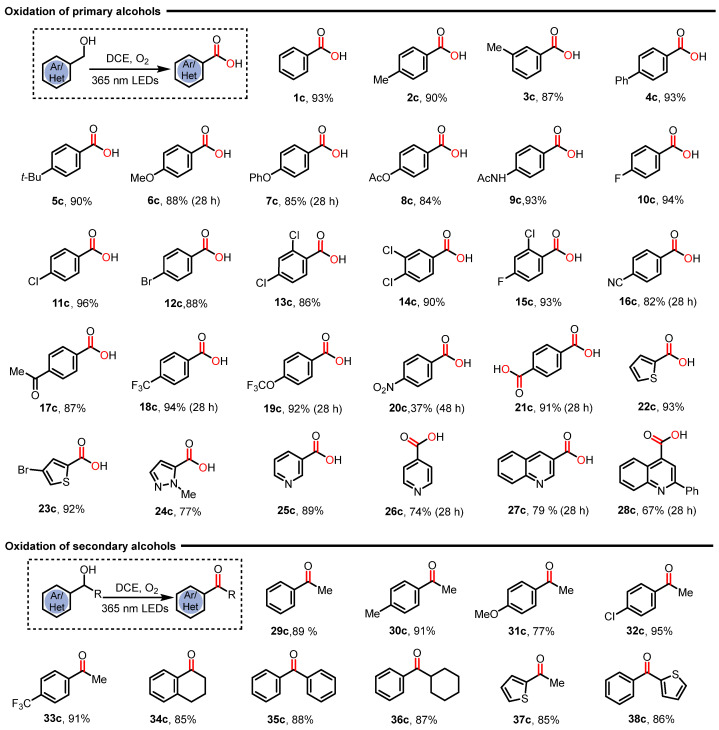
Substrate scope studies. Standard conditions: substrates (1.0 mmol), DCE (2 mL), O_2_ (1 atm), 20 °C, 365 nm LEDs (25 w), 8 h, unless otherwise stated. Yields were obtained by flash chromatography.

By utilizing the optimized reaction conditions, we expanded the substrate scope of the photo protocol (Figure 1). A wide range of benzylic alcohols containing electron-donating groups were all smoothly oxidized into the corresponding acids with good yields (**1**–**9c**, 76–94%). When substrates bearing electron-withdrawing groups, such as halogen (F, Cl, and Br), –CN, –COCH_3_, –CF_3_, and –OCF_3_, were examined, excellent yields of the corresponding acids were obtained (**10**–**19c**, 82–96%). In the case of 4-nitrobenzyl alcohol oxidation, the light exposure was prolonged for 48 h to obtain a 37% yield of 4-nitrobenzoic acid (**20c**). Bifunctional benzene-1,4-dimethanol was also conveniently converted to *p*-phthalic acid (**21c**) with a 91% yield. As a class of five membered hetero substrates were examined under the standard conditions, high yields of (hetero)aromatic acids were obtained (**22**–**24c**, 77–93%). Commendably, common dysoxidizable pyridinemethanols and quinolinemethanols were also susceptible to this protocol, leading to the desired acids in good yields (**25**–**28c**, 67–79%), yet requiring a longer reaction time (28 h). Next, we turned to the application of this method for the oxidation of secondary benzylic alcohols. Under similar conditions, a range of secondary benzylic alcohols bearing substituents with different electron effects were all compatible, outputting the corresponding aryl ketones in excellent yields (**29**–**36c**, 74–95%). Two secondary heteroaryl alcohols containing thiophene scaffold proceeded this oxidation efficiently to give the corresponding heteroaryl ketones **37c** (85%) and **38c** (86%), respectively. To preliminarily evaluate the scalability of this external-catalyst-free photooxidation, an experiment was conducted with **1a** at a 10 mmol scale based on the standard conditions. **1c** was conveniently obtained with a commendable yield (94%) (Supplementary Materials, Section S4).

**Scheme 1:**
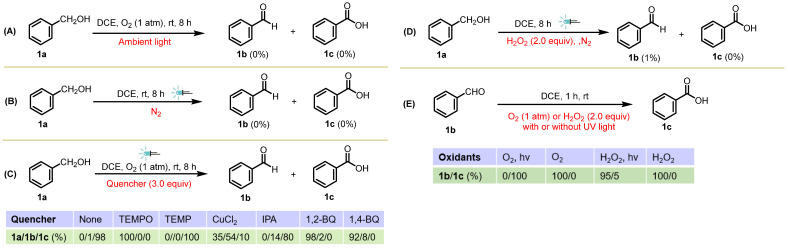
Control experiment on the oxidation of **1a** with conditions variation: (**A**) without UV light irradiation, (**B**) in N_2_ atmosphere, (**C**) by the addition of chemical quenching reagents, and (**D**) using H_2_O_2_ oxidant in N_2_ atmosphere. (**E**) Control experiments on the oxidation of **1b** with O_2_ (1 atm) and H_2_O_2_ (2.0 equiv) in presence or absence of UV light.

To obtain more clues on the underlying mechanism of this oxidation, mechanistic studies were performed. When the reaction of **1a** was performed under ambient light exposure or in the absence of O_2_, no desired product was produced within 8 h, indicating the necessity of both O_2_ and UV light irradiation (Scheme 1A,B). Drawing from the O_2_-absent experiment, we also ruled out the possible direct dehydrogenation event of alcohol. To disclose the underlying reactive species in the oxidation process, an array of chemical quenching experiments was then performed (Scheme 1C) [28,29,30]. When 2,2,6,6-tetramethylpiperdine-N-oxyl (TEMPO, 3.0 equiv) was submitted into the reaction mixture, the desired reaction was completely prevented according to GC–MS monitoring, indicating a cascade radical mechanism. The addition of 2,2,6,6-tetramethyl-4-piperidine (TEMP, a ^1^O_2_ scavenger, 3.0 equiv) or isopropyl alcohol (IPA, a HO^•^ scavenger, 3.0 equiv) exerted no significant influence on the conversion rate of **1a**, suggesting that ^1^O_2_ and HO^•^ were not the operative ROS involved in the rate-limiting step. When CuCl_2_ (30 mol%) was added as a recognized electron scavenger, a 50% yield of **1b** was still produced after irradiation for 8 h, indicating that SET to O_2_ or ^1^O_2_ must not be integrated in the primary mechanism. As 1,2- benzoquinone or 1,4-benzoquinone (BQ, a HOO^•^/^•^O_2_^−^ scavenger, 3.0 equiv) was added thereto, the conversion of **1a** was almost completely prevented, and 1,2-BQ showed a slightly stronger inhibition than 1,4-BQ. This suggested that the ROS involved in the turnover-limiting step of the reaction was more likely to be HOO^•^ rather than ^•^O_2_^−^ [31,32]. To clarify whether H_2_O_2_ played a role in this oxidation, a control experiment was carried out by the addition of H_2_O_2_ into **1a** solution, leading to no obvious formation of **1b** and **1c** after 8 h of UV light exposure in a N_2_ atmosphere. This disclosed that H_2_O_2_ could not serve as an initiating agent (Scheme 1D). Followingly, the oxidation of **1b** was examined individually with O_2_ and H_2_O_2_ (2.0 equiv) (Scheme 1E). With 365 nm light irradiation, the oxidation of **1b** in the presence of O_2_ could be completed within 1 h to give **1c** in a 100% assay yield, while the presence of H_2_O_2_ only resulted in a trace yield of **1c** within the same reaction time. Of note, the high efficiency of O_2_ displayed here also indicated the probable UVA sensitivity of benzaldehyde, since, in the absence of UV light, O_2_ actually failed to result in the obvious formation of **1c** within 1 h. These experiments also indicated that H_2_O_2_ exerted little influence on the radical cascade.

**Figure 2:**
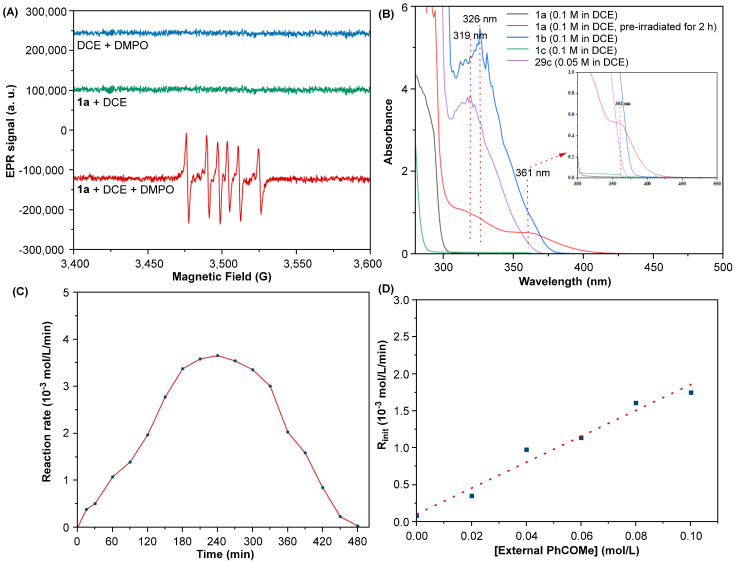
(**A**) EPR experiments with DMPO. (**B**) UV–vis spectroscopy studies. (**C**) Time course of depletion of **1a**. (**D**) R_init_ dependence on the concentrations of acetophenone.

Then, to evidence the radical species involved in the reaction, in situ EPR spectroscopy was performed using 5,5-dimethyl-1-pyrroline N-oxide (DMPO) as the radical probe. As displayed in Figure 2A, six characteristic signal peaks of the α-hydroxybenzyl adduct of DMPO (DMPO–C_6_H_5_CH^•^OH) were observed with diagnostic hydrogen and nitrogen hyperfine splittings for the nitroxide nitrogen as α_H_ 21.4 and α_N_ 13.7, respectively, which are in agreement with previous reports [33,34]. Furthermore, to obtain deeper insights into the question of how C_6_H_5_CH^•^OH species are formed in the absence of classical photosensitizers, UV–vis spectroscopy investigations were carried out individually with benzyl alcohol (**1a**), benzaldehyde (**1b**), benzoic acid (**1c**), acetophenone, and a reaction mixture that was pre-irradiated for 2 h. As shown in Figure 2B, both **1a** and **1c** displayed no prominent absorption band near 365 nm, while benzaldehyde and acetophenone presented a definite absorption band between 310 and 360 nm, in which the absorption at the 360 nm band was assigned to the dipole forbidden n→π* electronic transition of the carbonyl group [35]. As imaged, pre-irradiating the benzyl alcohol solution in an O_2_ atmosphere for 2 h allowed for the observation of a similar but a broadened absorption band that extended to about 375 nm. These results indicated the autocatalytic character of this external-catalyst-free oxidation driven by the response of carbonyl groups in aryl aldehydes and ketones near the 360 nm band.

To further understand the autocatalytic feature of the present reaction, we performed a series of kinetics studies. Figure 2C demonstrates clearly a definite self-accelerating character, though the induction period seemingly did not appear. Next, oxidation-stable acetophenone as an external catalyst was added with different loadings to the diluted DCE solution of **1a** for reaction rate measurements. The initial reaction rates (R_init_) were calculated by GC data for the consumption of **1a** within the first 15 min in 5 min increments. As Figure 2D depicts, a linear and positive rate dependence on the concentrations of acetophenone confirms the crucial catalytic role of aromatic aldehydes and ketones in this autocatalytic process. This result also suggests that the rate-determining transition state might integrate both the excited triplet aldehydes (or ketones) and alcohol substrates.

**Scheme 2:**
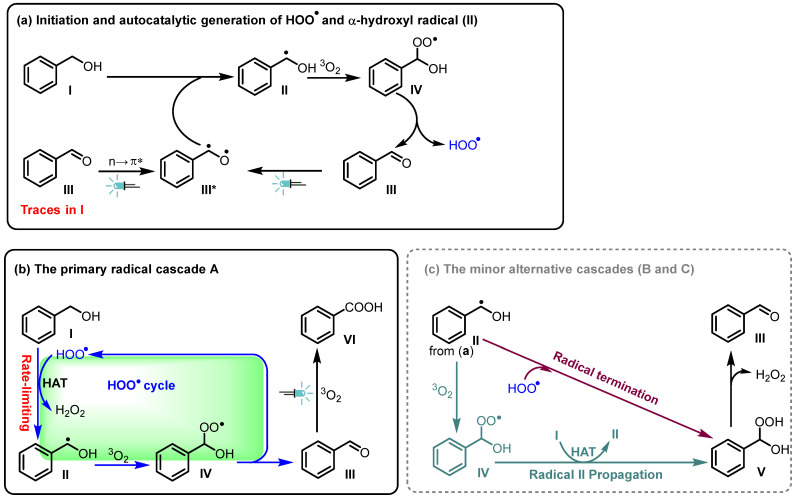
Plausible mechanism for the catalyst-free photooxidation of benzylic alcohols.

**Figure 3:**
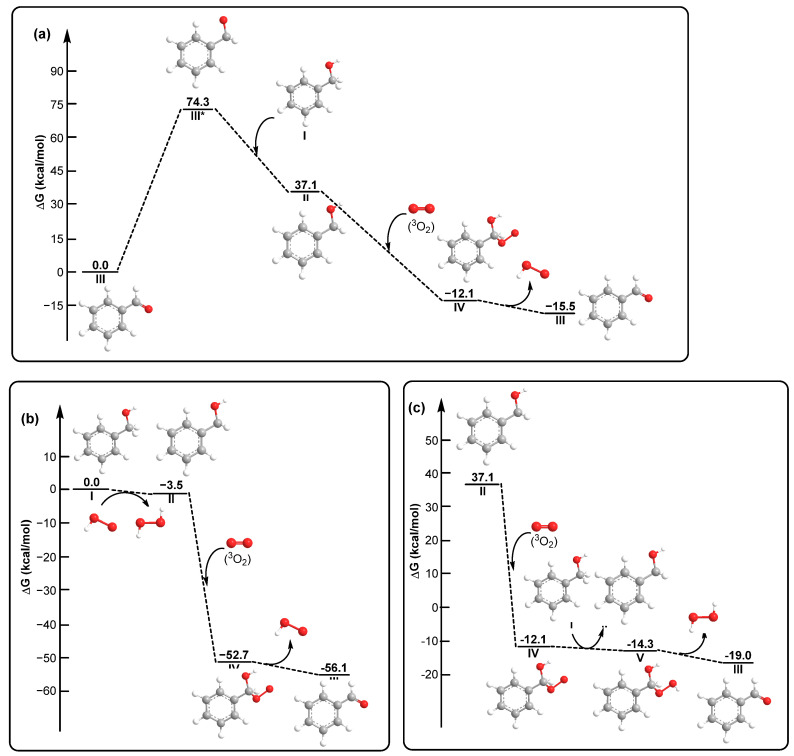
(**a**) Free energy profile for the initiation of HOO^•^. (**b**) Free energy profile for the Radical Cascade **A** (**I**→**II**→**IV**→**III**). (**c**) Free energy profile for the Radical Cascade **C** (**II**→**IV**→**V**→**III**). The potential energies shown in figures are in units of kcal/mol.

Based on the above analysis, a plausible mechanism for this catalyst-free oxidation was proposed (Scheme 2). At the induction stage, traces of aldehyde (**III**) contained in alcohol (**I**) were excited by UVA light through an electron transition n→π* to III*. An HAT from **I** to III* led to the formation of α-hydroxy radical **II**, which was captured by O_2_ to form **the** species **IV**. **IV** underwent β-elimination to allow for the release of the aldehyde **III** and HOO^•^ (Scheme 2a). Following this auto-acceleration stage, the aggressive HAT species HOO^•^ enabled a more efficient formation of **II** from **I** as the rate-limiting step, driven by the thermodynamically favored formation of H_2_O_2_ (bond dissociation energies of H-O bonds in H_2_O_2_ is about 87 Kcal/mol) [36,37]. Importantly, a dominant cascade from **I**→**II**→**IV**→**III** (Cascade **A**, Scheme 2b) eased the desired cycling of HOO^•^ and propagation of **II**, which could boost the oxidation reaction. The light-driven oxidation of **III** by O_2_ (or HOO^•^, rather than H_2_O_2_) then readily produced the ultimate aromatic acid (**VI**). In addition to Cascade **A**, two alternative paths from **II** to **III** were also assumed as related studies [25,26] (Scheme 2c), including Cascade **B** (**II**→**V**→**III**, marked with red arrows) and Cascade **C** (**II**→**IV**→**V**→**III**, marked with green arrows). Since Cascade **B** is a radical–radical termination, we confirmed that it should not be involved as the dominant cascade. In terms of Cascade **C**, it fails to explain the cruciality of HOO^•^ for the overall chain transfer that was confirmed by the above-described BQ-quenching results. To further evaluate the rationality of the proposed mechanism, density functional theory calculations (DFT) were performed with the Gaussian16 software package at the b3lyp/6-311++G* level of theory [38]. As shown in Figure 3a, III* that is formed from **III** by UVA induction (+74.3 Kcal/mol) abstracted a hydrogen atom from **I** to generate two molecules of II (+37.1 Kcal/mol). As shown in Figure 3b, HOO^•^ was cycled via Cascade **A** (**I**→**II**→**IV**→**III**, −56.1 Kcal/mol), wherein each step was thermodynamically favored. In this cascade, HAT from **I** by HOO^•^ was the rate-determining step. In comparison, Cascade **B** shown in Figure 3c was less favored, since it must start from high-potential intermediate **II**. This cascade should base on the initiating process of III* + **I**→**II** as the rate-determining step, which requires overcoming a relatively higher energy barrier of +37.1 Kcal/mol. Hereto, the critical operative ROS and its evolution, the turnover rate-limiting step, and the dominant radical cascade were clarified.

## 3. Experimental Section

### 3.1. General Information

The starting materials and reagents were commercially purchased and used without further purification, unless specifically mentioned. Gas chromatography–mass spectrometry (GC–MS) was carried out on Thermo Fisher Trace 1300 gas chromatograph systems (Thermo Fisher, Norristown, PA, USA) using a TRACE TR-5MS GC chromatographic column. NMR spectra were recorded on a Bruker WM 400 spectrometer (Billerica, MA, USA) (400 MHz for 1H, 100 MHz for 13C, and 376 MHz for 19F) at 298 K, unless otherwise indicated. Chemical shifts δ are given in ppm, using residual solvent as an internal standard. Coupling constants J are reported in Hz. High-resolution mass spectra were obtained on Acquity UPLC/XEVO G2-XS QTOF (Waters, MA, USA), equipped with a linear ion trap and orbitrap analyzers (Thermo Scientific, Waltham, MA, USA). The EPR measurements were performed on a Bruker Model A200 spectrometer (Bruker Instrument, Karlsruhe, Germany) equipped with a Bruker ER4112SHQ X-band resonator. UVvvis spectra were recorded on a TU-1900 UV–vis spectrophotometer (Shimadzu, Kyoto, Japan). The progress of the reactions was monitored by thin-layer chromatography using TLC plates and visualized by shortwave ultraviolet light. Flash chromatography was performed with Qingdao Dingkang flash silica gel (Qingdao Dingkang Silicone Co., Ltd, Qingdao, China) (200–300 mesh). Melting points were measured on a WRS-1C Melt-Temp apparatus (Wuhan Bonnin Technology Ltd., Wuhan, China) and were uncorrected. The photocatalytic reactions were performed using 10 mL or 30 mL quartz tubes placed in the parallel light reaction instrument, and the model was Titan PR-6 (Figure S1). The reaction temperature was measured using a thermometer when the reactions were complete, and reproducible results (20–25 °C) indicated that the reactions were performed consistently within this range.

### 3.2. General Procedure for the Oxidation

Alcohol substrate (1 mmol) and DCE (2 mL) were added in a 10 mL quartz tube, which was then vacuumed and purged with nitrogen via a nitrogen balloon three times. Then, the tube was placed into the reactor and the reaction mixture was stirred vigorously under UV irradiation (365 nm, 25 W) for a given time. The reaction was monitored with TLC. At the end of the reaction, the mixture was concentrated under vacuum to remove the solvent. The residue was purified over a column of silica gel to afford the corresponding carbonyls.

Compounds **1**–**38c** were synthesized according to this procedure.

### 3.3. Characterization Data of Products ***1–38c***

*Benzoic acid (**1c**)* [39]: white solid (113 mg, 93% yield); mp 124–125 °C; ^1^H NMR (400 MHz, CDCl_3_) δ 12.41 (s, 1H), 8.14 (dt, *J* = 8.4, 1.5 Hz, 2H), 7.65–7.58 (m, 1H), 7.51–7.45 (m, 2H); ^13^C NMR (400 MHz, CDCl_3_) δ 172.74, 133.93, 130.33, 129.45, 128.59.

*4-Methylbenzoic acid (**2c**)* [39]: white solid (122 mg, 90% yield); mp 181–183 °C; ^1^H NMR (400 MHz, DMSO-*d*6) δ 12.81 (s, 1H), 7.85 (d, *J* = 8.1 Hz, 2H), 7.26 (d, *J* = 8.1 Hz, 2H), 2.33 (s, 3H); ^13^C NMR (101 MHz, DMSO-*d*6) δ 167.81, 143.42, 129.78, 129.50, 128.48, 21.50.

*3-Methylbenzoic acid (**3c**)* [39]: white solid (118 mg, 87% yield), mp 112–13 °C; ^1^H NMR (400 MHz, DMSO-*d*6) δ 12.33 (s, 1H), 7.85–7.68 (m, 2H), 7.42–7.31 (m, 2H), 2.33 (s, 3H); ^13^C NMR (101 MHz, DMSO-*d*6) δ 167.91, 138.28, 133.83, 131.18, 130.18, 128.81, 126.90, 21.19.

*Biphenyl-4-carboxylic acid (**4c**)* [39]: white solid (186 mg, 94% yield); mp 221–224 °C; ^1^H NMR (400 MHz, DMSO-*d*6) δ 12.98 (s, 1H), 8.03 (d, *J* = 8.4 Hz, 2H), 7.81 (d, *J* = 8.4 Hz, 2H), 7.74 (d, *J* = 7.2 Hz, 2H), 7.51 (t, *J* = 7.5 Hz, 2H), 7.43 (t, *J* = 7.3 Hz, 1H); ^13^C NMR (101 MHz, DMSO-*d*6) δ 167.59, 144.75, 139.48, 130.42, 130.12, 129.54, 128.74, 127.42, 127.27.

*4-tert-Butylbenzoic acid (**5c**)* [40]: white solid (160 mg, 90% yield), mp 164–165 °C (lit [40] 164.5–165.5 °C); ^1^H NMR (400 MHz, DMSO-*d*6) δ 12.75 (s, 1H), 7.91 (d, *J* = 8.5 Hz, 2H), 7.40 (d, *J* = 8.5 Hz, 2H), 1.20 (s, 9H); ^13^C NMR (101 MHz, DMSO-*d*6) δ 167.75, 156.03, 129.69, 128.59, 125.54, 34.97, 31.15.

*4-Methoxybenzoic acid (**6c**)* [41]: white solid (134 mg, 88% yield); mp 185–186 °C; ^1^H NMR (400 MHz, DMSO-*d*6) δ 12.67 (s, 1H), 7.91 (d, *J* = 8.0 Hz, 2H), 7.09–6.92 (m, 2H), 3.82 (s, 3H); ^13^C NMR (101 MHz, DMSO-*d*6) δ 167.49, 163.28, 131.80, 123.40, 114.23, 55.84.

*4-phenoxybenzoic acid (**7c**)* [42]: white solid (182 mg, 85% yield); mp 159–161 °C; ^1^H NMR (400 MHz, DMSO-*d*6) δ 8.05–7.89 (m, 2H), 7.43 (tt, *J* = 7.6, 2.2 Hz, 2H), 7.26–7.17 (m, 1H), 7.16–7.05 (m, 2H), 7.05–6.97 (m, 2H); ^13^C NMR (101 MHz, DMSO-*d*6) δ 167.25, 161.43, 155.56, 132.12, 130.72, 125.06, 120.36, 117.60.

*4-Acetyloxybenzoic acid (**8c**)* [42]: white solid (180 mg, 84% yield); mp 189–192 °C; ^1^H NMR (400 MHz, DMSO-*d*6) δ 12.53 (s, 1H), 8.09–7.96 (m, 2H), 7.27 (s, 2H), 2.29 (s, 3H); ^13^C NMR (101 MHz, DMSO-*d*6) δ 169.28, 167.13, 154.38, 131.32, 128.86, 122.45, 21.24.

*4-Acetamido benzoic acid (**9c**)* [43]: white solid (170 mg, 93% yield); mp 260–262 °C; ^1^H NMR (400 MHz, DMSO-*d*6) δ 12.67 (s, 1H), 10.18 (s, 1H), 7.93 (d, *J* = 8.7 Hz, 2H), 7.72 (d, *J* = 8.7 Hz, 2H), 2.09 (s, 3H); ^13^C NMR (101 MHz, DMSO-*d*6) δ 169.35, 167.50, 143.73, 130.83, 125.39, 118.73, 24.49.

*4-Fluorobenzoic acid (**10c**)* [41]: white solid (132 mg, 94% yield); mp 180–182 °C; ^1^H NMR (400 MHz, DMSO-*d*6) δ 13.02 (s, 1H), 8.00 (dd, *J* = 8.3, 5.8 Hz, 2H), 7.28 (t, *J* = 8.8 Hz, 2H); ^13^C NMR (101 MHz, DMSO-*d*6) δ 166.83, 165.35 (d, *J* = 250.6 Hz), 132.51, 127.78, 115.96 (d, *J* = 22.0 Hz); ^19^F NMR (376 MHz, DMSO-*d*6) δ -106.92.

*4-Chlorobenzoic acid (**11c**)* [42]: white solid (150 mg, 95% yield); mp 241–243 °C; ^1^H NMR (400 MHz, DMSO-*d*6) δ 13.15 (s, 1H), 7.94 (s, 2H), 7.53 (s, 2H); ^13^C NMR (101 MHz, DMSO-*d*6) δ 166.92, 138.26, 131.55, 130.07, 129.10.

*4-Bromobenzoic acid (**12c**)* [39]: white solid (174 mg, 88% yield); mp 252–254 °C; ^1^H NMR (400 MHz, DMSO-*d*6) δ 13.19 (s, 1H), 7.87 (s, 2H), 7.69 (s, 2H); ^13^C NMR (101 MHz, DMSO-*d*6) δ 167.07, 132.11, 131.72, 130.44, 127.33.

*2,4-Dichlorobenzoic acid (**13c**)* [39]: white solid (164 mg, 86% yield); mp 161–162 °C; ^1^H NMR (400 MHz, DMSO-*d*6) δ 13.38 (s, 1H), 7.80 (d, *J* = 8.4 Hz, 1H), 7.58 (d, *J* = 2.0 Hz, 1H), 7.43 (dd, *J* = 8.4, 2.0 Hz, 1H); ^13^C NMR (101 MHz, DMSO-*d*6) δ 166.18, 137.03, 133.69, 132.86, 130.59, 130.32, 127.74.

*3,4-Dichlorobenzoic acid (**14c**)* [39]: white solid (172 mg, 90% yield); mp 209–210 °C; ^1^H NMR (400 MHz, DMSO-*d*6) δ 13.30 (s, 1H), 8.07 (d, *J* = 1.9 Hz, 1H), 7.89 (dd, *J* = 8.4, 1.9 Hz, 1H), 7.78 (s, 1H); ^13^C NMR (101 MHz, DMSO-*d*6) δ 165.87, 136.24, 131.97, 131.82, 131.46, 131.40, 129.74.

*2-Chloro-4-fluorobenzoic acid (**15c**)* [42]: white solid (162 mg, 90% yield); mp 180–181 °C; ^1^H NMR (400 MHz, DMSO-*d*6) δ 13.48 (s, 1H), 7.91 (dd, *J* = 8.7, 6.3 Hz, 1H), 7.56 (dd, *J* = 8.9, 2.6 Hz, 1H), 7.33 (td, *J* = 8.6, 2.5 Hz, 1H); ^13^C NMR (101 MHz, DMSO-*d*6) δ 166.17, 163.60 (d, *J* = 252.8 Hz), 134.17 (d, *J* = 11.1 Hz), 133.74 (d, *J* = 9.8 Hz), 128.07 (d, *J* = 3.5 Hz), 118.54 (d, *J* = 25.2 Hz), 114.98 (d, *J* = 21.3 Hz); ^19^F NMR (376 MHz, DMSO-*d*6) δ -106.75.

*4-Cyanobenzoic acid (**16c**)* [42]: white solid (121 mg, 82% yield); mp 221–222 °C; ^1^H NMR (400 MHz, DMSO-*d*6) δ 13.41 (s, 1H), 8.01 (d, *J* = 8.3 Hz, 2H), 7.85 (d, *J* = 8.3 Hz, 2H); ^13^C NMR (101 MHz, DMSO-*d*6) δ 166.42, 135.19, 132.85, 130.26, 118.52, 115.49.

*4-Acetylbenzoic acid (**17c**)* [42]: white solid (142 mg, 94% yield); mp 207–209 °C; ^1^H NMR (400 MHz, DMSO-*d*6) δ 13.34 (s, 1H), 8.05 (s, 4H), 2.63 (s, 3H); ^13^C NMR (101 MHz, DMSO-*d*6) δ 198.17, 167.10, 140.28, 129.91, 128.77, 27.46.

*4-(Trifluoromethyl) benzoic acid (**18c**)* [41]: white solid (171 mg, 90% yield); mp 218–219 °C; ^1^H NMR (400 MHz, DMSO-*d*6) δ 13.34 (s, 1H), 8.12 (d, *J* = 8.1 Hz, 2H), 7.82 (d, *J* = 8.2 Hz, 2H); ^13^C NMR (101 MHz, DMSO-*d*6) δ 166.64, 135.01, 132.78 (q, *J* = 32.0 Hz), 130.49, 125.90, 124.21 (d, *J* = 272.7 Hz); ^19^F NMR (376 MHz, DMSO-*d*6) δ -61.82.

*4-(Trifluoromethoxy) benzoic acid (**19c**)* [44]: white solid (190 mg, 92% yield); mp 152–153 °C; ^1^H NMR (400 MHz, DMSO-*d*6) δ 13.24 (s, 1H), 8.11–7.98 (m, 2H), 7.39 (d, *J* = 8.1 Hz, 2H); ^13^C NMR (101 MHz, DMSO-*d*6) δ 166.62, 151.90, 132.02, 130.23, 120.87,120.82(m); ^19^F NMR (376 MHz, DMSO-*d*6) δ -57.32.

*4-Nitrobenzoic acid (**20c**)* [44]: white solid (62 mg, 37% yield); mp 241–242 °C; ^1^H NMR (400 MHz, DMSO-*d*6) δ 12.42 (s, 1H), ), 8.30–8.23 (m, 2H), 8.15–8.08 (m, 2H); ^13^C NMR (101 MHz, DMSO-*d*6) δ 166.21, 150.36, 136.75, 131.07, 124.05.

*Terephthalic acid (**21c**)* [39]: white solid (151 mg, 91% yield); mp 318–321 °C; ^1^H NMR (400 MHz, DMSO-*d*6) δ 13.31 (s, 2H), 8.04 (s, 4H); ^13^C NMR (101 MHz, DMSO-*d*6) δ 167.13, 134.88, 129.90.

*2-Thiophenic acid (**22c**)* [41]: white solid (119 mg, 93% yield); mp 126–127 °C; ^1^H NMR (400 MHz, DMSO-*d*6) δ 13.02 (s, 1H), 7.79 (dd, *J* = 5.0, 1.3 Hz, 1H), 7.73 (dd, *J* = 3.7, 1.3 Hz, 1H), 7.13 (dd, *J* = 5.0, 3.7 Hz, 1H); ^13^C NMR (101 MHz, DMSO-*d*6) δ 163.43, 135.16, 133.62, 133.46, 128.56.

*4-Bromo-2-thiophenecarboxylic acid (**23c**)* [45]: white solid (190 mg, 92% yield); mp 122–124 °C; ^1^H NMR (400 MHz, DMSO-*d*6) δ 12.49 (s, 1H), 7.91 (s, 1H), 7.62 (s, 1H); ^13^C NMR (101 MHz, DMSO-*d*6) δ 162.60, 138.30, 134.29, 130.44, 109.71.

*1-Methyl-1H-pyrazole-5-carboxylic acid (**24c**)* [46]: white solid (97 mg, 77% yield); mp 222–225 °C; ^1^H NMR (400 MHz, DMSO-*d*6) δ 12.25 (s, 1H), 7.49 (s, 1H), 6.81 (s, 1H), 4.07 (s, 3H); ^13^C NMR (101 MHz, DMSO-*d*6) δ 161.16, 137.89, 133.39, 111.44, 39.58.

*Nicotinic Acid (**25c**)* [47]: white solid (109 mg, 89% yield); mp 236–238 °C; ^1^H NMR (400 MHz, DMSO-*d*6) δ 9.07 (s, 1H), 8.76 (d, *J* = 4.7 Hz, 1H), 8.25 (d, *J* = 7.9 Hz, 1H), 7.50 (dd, *J* = 7.7, 5.0 Hz, 1H); ^13^C NMR (101 MHz, DMSO-*d*6) δ 166.71, 153.56, 150.62, 137.41, 127.02, 124.16.

*4-Pyridinecarboxylic acid (**26c**)* [48]: white solid (91 mg, 74% yield); mp 314–315 °C; ^1^H NMR (400 MHz, DMSO-*d*6) δ 13.20 (s, 1H), 8.78 (d, *J* = 4.8 Hz, 2H), 7.82 (d, *J* = 4.8 Hz, 2H); ^13^C NMR (101 MHz, DMSO-*d*6) δ 166.65, 151.07, 138.52, 123.21.

*Quinoline-3-carboxylic acid (**27c**)* [49]: white solid (137 mg, 79% yield); mp 277–279 °C; ^1^H NMR (400 MHz, DMSO-*d*6) δ 13.25 (s, 1H), 9.32 (s, 1H), 8.98 (s, 1H), 8.19 (d, *J* = 8.2 Hz, 1H), 8.10 (d, *J* = 8.5 Hz, 1H), 7.91 (t, *J* = 7.7 Hz, 1H), 7.71 (t, *J* = 7.5 Hz, 1H); ^13^C NMR (101 MHz, DMSO-*d*6) δ 166.78, 150.28, 149.54, 138.94, 132.38, 130.01, 129.22, 127.92, 127.04, 124.07.

*2-Phenylquinoline-4-carboxylic acid (**28c**)* [50]: white solid (167 mg, 67% yield); mp 214–215 °C; ^1^H NMR (400 MHz, DMSO-*d*6) δ 13.78 (s, 1H), 8.71 (d, *J* = 8.5 Hz, 1H), 8.48 (s, 1H), 8.28 (d, *J* = 7.4 Hz, 2H), 8.16 (d, *J* = 8.4 Hz, 1H), 7.82 (t, *J* = 7.6 Hz, 1H), 7.68 (t, *J* = 7.7 Hz, 1H), 7.53 (dt, *J* = 12.0, 6.9 Hz, 3H); ^13^C NMR (101 MHz, DMSO-*d*6) δ 168.12, 156.22, 148.88, 138.37, 137.96, 130.56, 130.36, 130.24, 129.38, 128.15, 127.65, 125.90, 123.98, 119.68.

*Acetophenone (**29c**)* [39]: colorless oil (109 mg, 89% yield); ^1^H NMR (400 MHz, CDCl_3_) δ 7.93 (d, *J* = 7.5 Hz, 2H), 7.52 (t, *J* = 7.3 Hz, 1H), 7.42 (t, *J* = 7.6 Hz, 2H), 2.56 (s, 3H); ^13^C NMR (101 MHz, CDCl3) δ 198.05, 137.06, 133.08, 128.55, 128.27, 26.57.

*4′-Methylacetophenone (**30c**)* [39]: colorless oil (122 mg, 91% yield); ^1^H NMR (400 MHz, DMSO-*d*6) δ 7.85 (d, *J* = 7.7 Hz, 2H), 7.31 (d, *J* = 7.8 Hz, 2H), 2.54 (s, 3H), 2.36 (s, 3H); ^13^C NMR (101 MHz, DMSO-*d*6) δ 197.78, 143.90, 134.84, 129.61, 128.70, 26.96, 21.54.

*4′-Methoxyacetophenone (**31c**)* [40]: colorless oil (116 mg, 77% yield); ^1^H NMR (400 MHz, DMSO-*d*6) δ 7.92 (d, *J* = 8.9 Hz, 2H), 7.00 (d, *J* = 8.9 Hz, 2H), 3.82 (s, 3H), 2.50 (s, 3H); ^13^C NMR (101 MHz, DMSO-*d*6) δ 196.57, 163.52, 130.85, 130.33, 114.16, 55.82, 26.66.

*4′-Chloroacetophenone (**32c**)* [51]: colorless oil (147 mg, 95% yield).; ^1^H NMR (400 MHz, DMSO-*d*6) δ 7.93–7.83 (m, 2H), 7.52–7.36 (m, 2H), 2.53 (s, 3H); ^13^C NMR (101 MHz, DMSO-*d*6) δ 196.82, 138.63, 135.72, 130.25, 128.98, 26.80 

*4′-Trifluoromethylacetophenone (**33c**)* [52]: colorless liquid (171 mg, 91% yield); ^1^H NMR (400 MHz, CDCl_3_) δ 8.04 (d, *J* = 8.2 Hz, 2H), 7.70 (d, *J* = 8.2 Hz, 2H), 2.63 (s, 3H); ^13^C NMR (101 MHz, Chloroform-d) δ 196.90, 139.63, 134.29 (q, *J* = 32.7 Hz), 128.57, 125.59, 123.58 (d, *J* = 272.6 Hz), 26.64; ^19^F NMR (376 MHz, CDCl_3_) δ -63.23.

*3,4-Dihydronaphthalen-1(2H)-one (**34c**)* [53]: colorless liquid (124 mg, 85% yield); ^1^H NMR (400 MHz, DMSO-*d*6) δ 7.89 (d, *J* = 7.8 Hz, 1H), 7.50 (t, *J* = 7.4 Hz, 1H), 7.36–7.24 (m, 2H), 2.89 (t, *J* = 5.9 Hz, 2H), 2.56 (t, *J* = 6.4 Hz, 2H), 1.99 (p, *J* = 5.9 Hz, 2H); ^13^C NMR (101 MHz, DMSO-*d*6) δ 197.73, 145.01, 133.74, 132.59, 129.36, 126.87, 126.70, 39.05, 29.37, 23.34.

*Benzophenone (**35c**)* [52]: colorless solid (160 mg, 88% yield), mp 47–49 °C; ^1^H NMR (400 MHz, DMSO-*d*6) δ 7.73 (d, *J* = 7.8 Hz, 4H), 7.67 (t, *J* = 7.4 Hz, 2H), 7.55 (t, *J* = 7.5 Hz, 4H); ^13^C NMR (101 MHz, DMSO-*d*6) δ 196.23, 137.46, 133.10, 130.06, 128.98.

*Cyclohexyl(phenyl)methanone (**36c**)* [52]: white solid (164 mg, 87% yield); mp 55–56 °C; ^1^H NMR (400 MHz, DMSO-*d*6) δ 7.95 (d, *J* = 7.8 Hz, 2H), 7.62 (t, *J* = 7.3 Hz, 1H), 7.52 (t, *J* = 7.5 Hz, 2H), 3.38 (d, *J* = 11.0 Hz, 2H), 1.72 (td, *J* = 26.8, 24.5, 12.7 Hz, 5H), 1.36 (dp, *J* = 23.6, 11.7, 10.9 Hz, 4H), 1.17 (q, *J* = 12.1 Hz, 1H); ^13^C NMR (101 MHz, DMSO-*d*6) δ 203.50, 136.24, 133.34, 129.18, 128.51, 44.86, 29.51, 26.06, 25.58.

*2-Acetylthiophene (**37c**)* [54]: light brown liquid (107 mg, 85% yield); ^1^H NMR (400 MHz, CDCl_3_) δ 7.51 (d, *J* = 3.7 Hz, 1H), 7.45 (d, *J* = 5.0 Hz, 1H), 6.94–6.90 (m, 1H), 2.34 (s, 3H); ^13^C NMR (101 MHz, CDCl_3_) δ 190.57, 144.35, 133.82, 132.69, 128.19, 26.66.

*Phenyl(thiophen-2-yl)methanone (**38c**)* [55]: colorless solid (162 mg, 86% yield); mp 55–57 °C; ^1^H NMR (400 MHz, CDCl_3_) δ 7.89–7.83 (m, 2H), 7.70 (d, *J* = 4.9 Hz, 1H), 7.65–7.61 (m, 1H), 7.58 (t, *J* = 7.4 Hz, 1H), 7.49 (t, *J* = 7.5 Hz, 2H), 7.18–7.11 (m, 1H); ^13^C NMR (101 MHz, CDCl_3_) δ 188.20, 143.60, 138.11, 134.95, 134.32, 132.33, 129.17, 128.46, 128.08.

## 4. Conclusions

In summary, an external-catalyst-free UVA-light-driven aerobic oxidation method was established in the presence of DCE solvent. It enables the high-yielding synthesis of various (hetero)aromatic acids and ketones. Comprehensive mechanistic investigations clarified the autocatalytic character of the oxidation, driven by the UVA-sensitivity of carbonyl groups in aromatic aldehydes and ketones. HOO^•^ proved to be the operative ROS as the real chain carrier involved in the primary radical cascade, and, thereby, the ambiguities and irrationality in previous reports have been revised. The present work serves as a cautionary note on strict mechanistic identification for UVA-driven redox reactions that involve the generation of aromatic aldehydes and ketones.

## Figures and Tables

**Table 1:** Conditions optimization for oxidation of benzyl alcohol into benzoic acid ^a^.

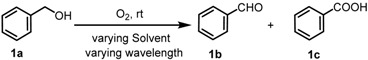					
Entry	Cat.	Solvent	Wavelength (nm)	Time (h)	1a/1b/1c (%) ^b^
1	-	MeCN	365	12	1/9/90
2	-	Benzene	365	12	34/37/29
3 ^c^	-	DCM	365	8	1/2/82
4	-	DCE	365	8	0/2/98
5	-	H_2_O	365	12	99/1/0
6	-	DMSO	365	8	8/52/40
7	-	Acetone	365	8	13/0/87
8	-	DCE	400	12	98/2/0
9	-	DCE	455	12	100/0/0
8 ^d^	TsOH	DCE	365	8	12/79/9
9 ^d^	MsOH	DCE	365	8	36/60/4
10 ^d^	TsOH	DCE	400	8	96/4/0
11 ^d^	MsOH	DCE	400	8	99/1/0

^a^ Reaction conditions: benzyl alcohol (**1a**, 1.0 mmol), solvent (2 mL), O_2_ (1 atm), 20 °C, 365–455 nm LEDs (25 w), unless specially mentioned. ^b^ Yields were assayed by GC**–**MS. ^c^ 5% benzyl chloride was observed. ^d^ Catalyst loadings: 10 mol%.

## Data Availability

The data presented in this study are available on request from the corresponding author.

## Supplementary Materials

The following supporting information can be downloaded at: https://www.mdpi.com/article/10.3390/molecules29143429/s1.
