# Supplementary material for: HOO• as the Chain Carrier for the Autocatalytic Photooxidation of Benzylic Alcohols
Source: Molecules. 2024 Jul 22;29(14):3429. doi: 10.3390/molecules29143429 (PMC11279666; doi:10.3390/molecules29143429)
Supplement: Supplementary file 1 [file molecules-29-03429-s001.zip › molecules-3008279-supplementary.pdf]

Supporting Information For

HOO$^{\bullet}$ as the chain–carrier for the autocatalytic photooxidation of benzylic alcohols

Xiao-Yu Wang,[a†] Huan-E Lao,[a†] Hao-Yue Zhang,[a] Yi Wang,[b] Qing Zhang,[a] Jie-Qing Wu,[a] Yu-Feng Li,[a*] Hong-Jun Zhu,[a*] Jian-You Mao,[a*] Yi Pan[b]

[a] *School of Chemistry and Molecular Engineering, Nanjing Tech University, Nanjing, 211816, China;*

[b] *School of chemistry and chemical engineering, Nanjing University, Nanjing, 210023, China;*

Contents:

S1. General information……..…………..………………….…………….…………………...………S2

S1.1 Materials

S1.2 Physical methods

S1.3 Photoredox reactor

S2. Conditions optimization……………..…………………………………….…………………....…S3

S3. General procedure for the oxidation.….……………..………………………….……….……...…S3

S4. Gram scale synthesis of benzoic acid..………................……………………………….…….…S3

S5. Mechanistic studies…..………………………………………………….…………….....…S4

S5.1 Control experiments

S5.2 EPR experiments

S5.3 UV–vis studies

S5.4 Kinetics Studie

S5.5 Quantum yield determination

S5.6 Density functional theory (DFT) calculation

S6. NMR spectra……………………………………….…………………………….……...S16

S1. General information

S1.1 Materials

Starting materials and reagents were purchased from Sigma–Aldrich, Acros, Innochem, Energy Chemical, TCI China or Alfa Aesar, and used without further purification.

S1.2 Physical methods

Gas chromatography–mass spectrometry (GC–MS) was carried out on Thermo Fisher Trace 1300 gas chromatograph systems using TRACE TR–5MS GC chromatographic column. NMR spectra were recorded on a Bruker WM 400 spectrometer (400 MHz for ^{1}H, 100 MHz for ^{13}C and 376 MHz for ^{19}F) at 298 K unless otherwise indicated. Chemical shifts δ are given in ppm, using residual solvent as an internal standard. Coupling constants J are reported in Hz. High–resolution mass spectra were obtained on Acquity UPLC/XEVO G2–XS QTOF, equipped with a linear ion trap and orbitrap analyzers. The EPR measurements were performed on a Bruker Model A200 spectrometer (Bruker Instrument, Germany) equipped with a Bruker ER4112SHQ X–band resonator. UV–vis spectra were recorded on TU–1900 UV–vis spectrophotometer. The progress of the reactions was monitored by thin–layer chromatography using TLC plates and visualized by shortwave ultraviolet light. Flash chromatography was performed with Qingdao Haiyang flash silica gel (200–300 mesh). Melting points were measured on a WRS–1C Melt–Temp apparatus and were uncorrected. The DFT calculations were carried out by using Gaussian16 program with all atoms fully relaxed.

S1.3 Photoredox reactor

The photocatalytic reactions were performed using 10 mL, or 30 mL quartz tubes placed in the parallel light reaction instrument, and the model is Titan PR–6 (Figure S1). The temperatures of the reaction mixtures were measured using a thermometer when the reactions were complete, and reproducible results (20–25 °C) indicated that the reactions were performed consistently within this range.

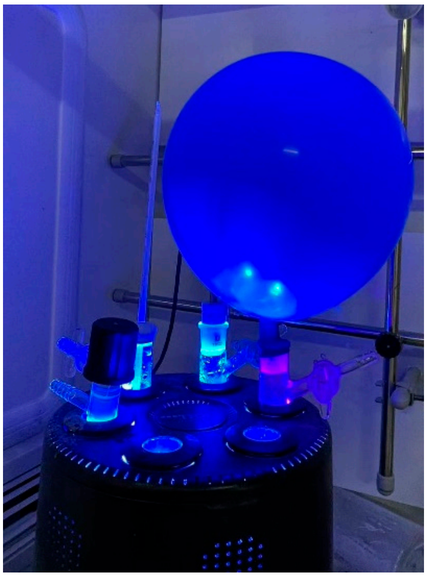

Figure S1. The photocatalytic reactor.

S2. Conditions optimization

All reactions were performed on a 1 mmol of scale in presence of 2.0 mL solvent with quartz tubes equipped with an O_2 balloon under ultraviolet (λ = 365 nm, 25 W) illumination at room temperature (20 °C) for 8 h unless specially noted. Yields of **1c** were assessed on GC–MS instrument.

S3. General procedure for the oxidation

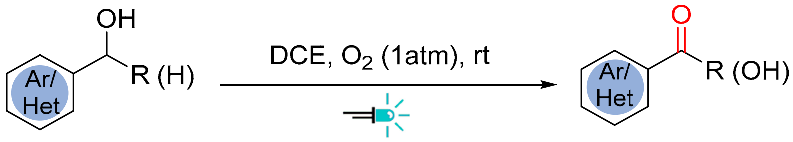

Alcohol substrate (1 mmol) and DCE (2 mL) were added in 10 mL quartz tube, which was then vacuumed and purged with O_2 via an O_2 balloon for three times. Then the tube was placed into the reactor and the reaction mixture was stirred vigorously under UV irradiation (365 nm) for a given time. The reaction was monitored with TLC. At the end of the reaction, the mixture was concentrated under vacuum to remove the solvent. The residue was purified over a column of silica gel to afford the corresponding carbonyls.

Compounds **1**–**38c** were synthesized according to this procedure.

S4. Gram scale synthesis of benzoic acid

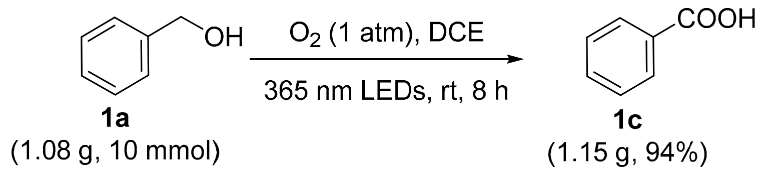

Benzyl alcohol (1.08 g, 10 mmol) and DCE (20 mL) were added in 30 mL quartz tube, which was then vacuumed and purged with O_2 via an O_2 balloon for three times. Then the tube was placed into the reactor and the reaction mixture was stirred vigorously under UV irradiation (365 nm, 25 w) for 8 h. At the end of the reaction, the mixture was concentrated under vacuum to remove the solvent. The residue was purified over a column of silica gel (eluant: EA : PE : MeOH = 1 : 10 : 1) to afford the benzoic acid (**1c**) as a white solid (1.15 g, 94%).

S5. Mechanistic studies

S5.1 Control experiments

S5.1.1 Control experiment on the oxidation of 1a in absence of UV light

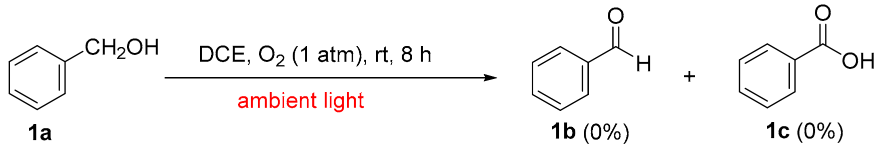

Benzyl alcohol (1 mmol) and DCE (2 mL) were added in 25 mL glass tube, which was then vacuumed and purged with O_2 via an O_2 balloon for three times. Then the reaction mixture was stirred vigorously under ambient light condition for 8 h. The reaction was subsequently monitored by GC–MS method.

S5.1.2 Control experiment on the oxidation of 1a in absence of O_2

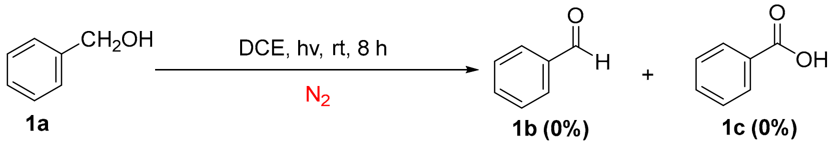

Benzyl alcohol (1 mmol) and DCE (2 mL) were added in 25 mL quartz tube, which was then vacuumed and purged with nitrogen via a nitrogen balloon for three times. Then the tube was placed into the reactor and the reaction mixture was stirred vigorously under UV irradiation (365 nm) for 8 h. The reaction was subsequently monitored by GC–MS method.

S5.1.3 Chemical quenching experiments on the oxidation of 1a

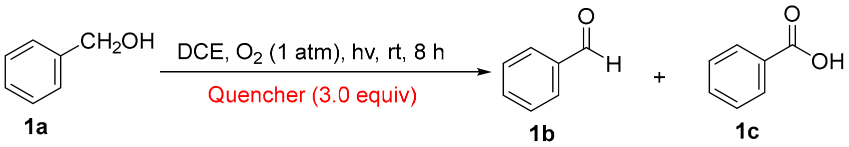

Benzyl alcohol (1 mmol), quenching reagent (3 mmol) and DCE (2 mL) were added in 10 mL quartz tube, which was then vacuumed and purged with O_2 via an O_2 balloon for three times. Then the tube was placed into the reactor and the reaction mixture was stirred vigorously under UV irradiation (365 nm) for 8 h. The reaction was subsequently monitored by GC–MS method.

S5.1.4 Control experiment on the oxidation of 1a with H_2O_2 under UV light

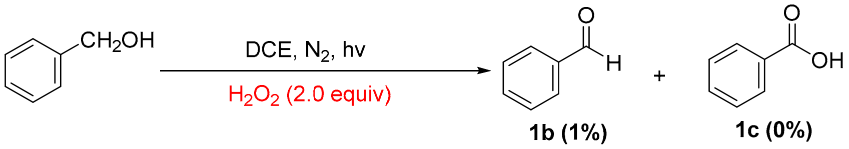

Benzyl alcohol (1 mmol), Hydrogen peroxide (25 w%, 2 mmol) and DCE (2 mL) were added in 10 mL quartz tube, which was then vacuumed and purged with nitrogen via a nitrogen balloon for three times. Then the tube was placed into the reactor and the reaction mixture was stirred vigorously under UV irradiation (365 nm) for 8 h. The reaction was subsequently monitored by GC–MS method.

S5.1.5 Control experiments on the oxidation of 1b with O_2 and H_2O_2

O_2 oxidation procedure: benzaldehyde (1 mmol) and 2 mL DCE were added in 10 mL quartz tube, which was then vacuumed and purged with O_2 via an O_2 balloon for three times. Then the tube was stirred exposed under 365 nm light or ambient light and the reaction mixture for 1 h. The reaction was subsequently monitored by GC–MS method (Table S1).

H_2O_2 oxidation procedure: benzaldehyde (1 mmol), DCE (2 mL) and hydrogen peroxide (25 w%, 2 mmol) were added in 10 mL quartz tube, which was then vacuumed and purged with nitrogen via a nitrogen balloon for three times. Then the tube was stirred exposed under 365 nm light or ambient light and the reaction mixture for 1 h. The reaction was subsequently monitored by GC–MS method (Table S1).

Table S1. Control experiments on the oxidation of **1b** with O_2 and H_2O_2.

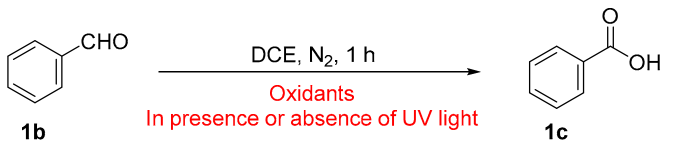

Entry	Oxidant	Light	**1b**/**1c** (Assay yields, %)[a]
1	O_2	365 nm	0/100
2[b]	O_2	–	100/0
3	H_2O_2	365 nm	95/5
4[b]	H_2O_2	–	100/0

[a]Assay yields were measured on GC–MS. [b]Under ambient light.

S5.2 EPR experiments

All samples were prepared in DCE solvent under O_2 (1 atm) atmosphere unless otherwise stated. DMPO was used as the trapping agents to monitor $HOO^{\bullet}$. The relative concentrations of **1a** : DMPO were 10 : 1 (1 M : 100 mM). After the addition of trapping agents, irradiation of each sample with 365 nm LEDs was maintained for 5 min. Samples were then transferred into 1.3 mm outer diameter (1 mm inner diameter) silica capillary EPR tubes (80 mm height; Wilmad LabGlass) and inserted into a 4 mm outer diameter (3 mm inner diameter) quartz tube. All EPR samples were measured at room temperature on a Bruker Model A200 spectrometer (Bruker Instrument, Germany) equipped with a Bruker ER4112SHQ X–band resonator.

S5.3 UV–vis experiments

UV–vis spectrum for benzyl alcohol (1a): Benzyl alcohol (108 mg, 1 mmol) and DCE (10 mL) were added into an oven dried Schlenk tube, which was then vacuumed and purged with Ar for three times. Then, the mixture was stirred at room temperature in dark for 15 min. The solution was subsequently used for UV–vis analysis.

UV–vis spectrum for benzyl alcohol (1a) after pre–irradiating for 2h: Benzyl alcohol (108 mg, 1 mmol) and DCE (10 mL) were added into a 25 mL quartz tube, which was then vacuumed and purged with O_2 via an O_2 balloon for three times. Then the tube was stirred under 365 nm light for 2 h. Then, the mixture was transferred into an oven dried Schlenk tube, which was then vacuumed and purged with Ar for three times. Then, the mixture was stirred at room temperature under 365 nm light (25 W) for 2 h. The solution was subsequently used for UV–vis analysis.

UV–vis spectrum for benzaldehyde (1b): Benzaldehyde (106 mg, 1 mmol) and DCE (10 mL) were added into an oven dried Schlenk tube, which was then vacuumed and purged with Ar for three times. Then, the mixture was stirred at room temperature in dark for 15 min. The solution was subsequently used for UV–vis analysis.

UV–vis spectrum for acetophenone (29c): Acetophenone (60 mg, 0.5 mmol) and DCE (10 mL) were added into an oven dried Schlenk tube, which was then vacuumed and purged with Ar for three times. Then, the mixture was stirred at room temperature in dark for 15 min. The solution was subsequently used for UV–vis analysis.

UV–vis spectrum for benzoic acid (1c): Benzoic acid (122 mg, 1 mmol) and DCE (10 mL) were added into an oven dried Schlenk tube, which was then vacuumed and purged with Ar for three times. Then, the mixture was stirred at room temperature in dark for 15 min. The solution was subsequently used for UV–vis analysis.

S5.4 Kinetics Studie

The standard curve with acetophenone as the internal standard: Different loadings of benzyl alcohol (0.25 mmol, 27 mg; 0.5 mmol, 54 mg; 0.75 mmol, 81 mg; 1 mmol, 108 mg; 1.25 mmol, 135 mg) were added into 25 mL volumetric flasks respectively. Into each of the volumetric flasks, internal standard of acetophenone (1 mmol, 120 mg) was added, and the volume was adjusted to 25.00 mL with DCE for use. The mixtures prepared here was analyzed on GC, and the retention time and peak area were recorded. The concentration ratios of **1a** and the internal standard (acetophenone) in the standard mixtures were listed as C_i/C_s under the x–axis, and the corresponding peak area ratios of **1a** and the internal standard (acetophenone) were listed as A_i/A_s along the y–axis, and the standard curve was depicted in Figure S2. The reciprocal of the slope represents the correction factor (f). The A_i/A_s and the standard deviation (S. D.) of A_i/A_s were obtained from three measurements for C_i/C_s (Table S2 and Figure S2).

Correction factor calculation formula:

$$\mathrm{f} = \frac{A_i \times C_s}{A_s \times C_i}$$

C_i: The concentration of **1a** in the standard mixture

C_s: The concentration of the internal standard (acetophenone)

A_i: The peak area of **1a** in the standard solution

A_s: The peak area of the internal standard (acetophenone)

Table S2. Standard curve data of 1a by internal standard GC.

C_i/C_s	A_i/A_s	S.D. of A_i/A_s
0	0	0
0.25	0.2209	0.0030
0.50	0.4115	0.0257
0.75	0.6799	0.0296
1.00	0.9478	0.0843
1.25	1.0023	0.0743

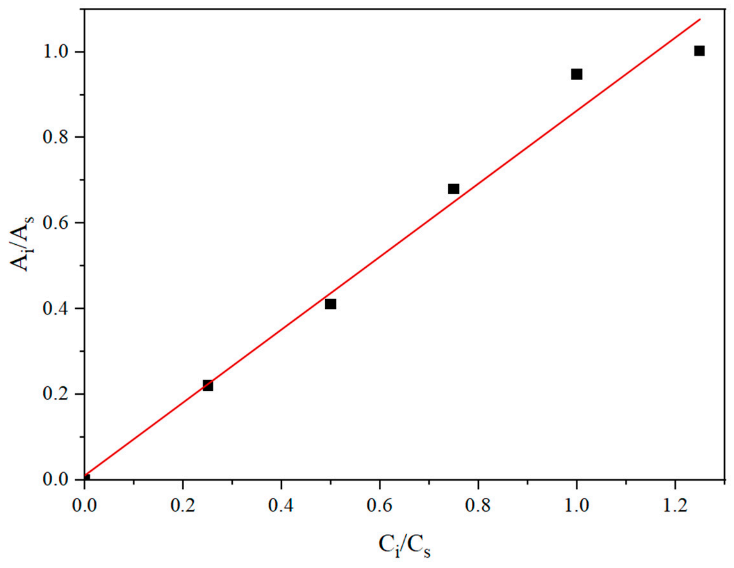

Figure S2. Standard curve of 1a by internal standard GC.

Table S3. Standard equation of benzyl alcohol.

Linear equation	R^2	f
y = 0.8526x + 0.0108	0.9823	1.1729

Time course of depletion of 1a: A 0.1 M solution of benzyl alcohol was prepared with DCE in a 10 mL volumetric flask. 2 mL of this solution was transferred into a 25 mL quartz tube. The tube was vacuumed and purged with oxygen via an oxygen balloon for three times. The mixture

was irradiated under 365 nm LEDs (25 w) and monitored by the addition of acetophenone as the internal standard for GC analysis. The reaction rates for depletion of **1a** at a fixed reaction time was calculated after three parallel experiments.

R_{init} dependence on the concentrations of external acetophone: A 0.1 M solution of benzyl alcohol was prepared with DCE in a 10 mL volumetric flask, containing 1 mmol of benzyl alcohol (1 mmol), different equivalents of acetophenone (0.2 mmol, 24 mg; 0.4 mmol, 48 mg; 0.6 mmol, 72 mg; 0.8 mmol, 96 mg; 1 mmol, 120 mg) and DCE solvent. The mixture was transferred into a 25 mL quartz tube, which was then vacuumed and purged with oxygen via an oxygen balloon for three times. The mixture was irradiated under 365 nm LEDs and measured on gas chromatography determination by dislodging 0.1 mL of each solution in a 5 min increment within 15 min. The calculation of initial reaction rates (R_{init}) based on the gas phase standard curve to obtain the average value after three parallel experiments. The average R_{init} and the standard deviation (S. D.) of R_{init} were obtained from three measurements for each concentration of acetophone (Table S4 and Figure S3).

Calculation formula:

$$m_i = \frac{A_i \times C_s \times \mathrm{V} \times \mathrm{M}}{A_s \times \mathrm{f}}$$

$$\triangle \mathrm{c} = \frac{m_1 - m_i}{\mathrm{M} \times \mathrm{V}}$$

$$\nu = \frac{\triangle \mathrm{c}}{\triangle \mathrm{t}}$$

f: Relative correction factor

m_i: Mass of components to be tested in the reaction solution

m_1: The initial mass of the component to be tested before reaction

V: Total volume of reaction liquid

M: The relative molecular weight of the tested component (benzyl alcohol)

△c: Change in the concentration of benzyl alcohol to be measured

△t: Reaction time

ν: Reaction rate

Table S4. Kinetic data for [acetophenone]–dependent reaction rates.

[External PhCOMe] (mol/L)	R_{init} (10^{-3} mol/L/min)	S.D. of rate (10^{-3} mol/L/min)
0	0.0774	0.0271
0.02	0.3491	0.1065
0.04	0.9744	0.1677
0.06	1.1372	0.0207
0.08	1.6085	0.0995
0.10	1.7470	0.1130

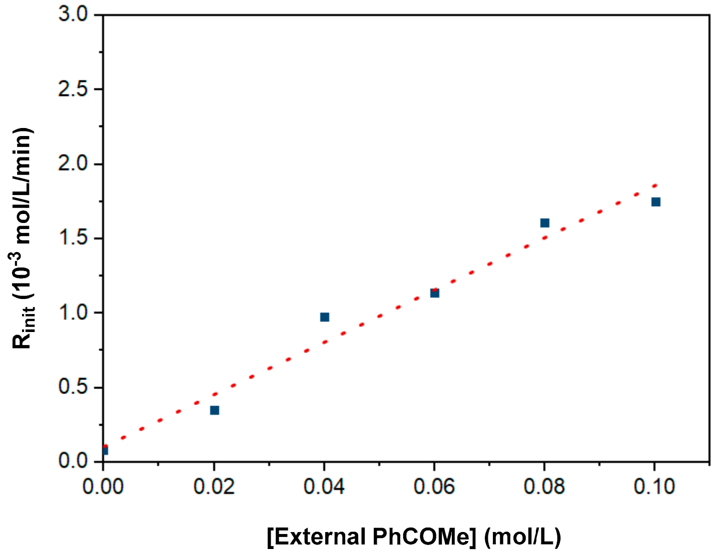

Figure S3. Kinetic data for [external acetophenone]–dependence on reaction rates.

S5.5 Quantum yield determination

Determination of the photon flux of the fluorometer at 365 nm: The photon flux of our photoreactor was determined by standard ferrioxalate actinometry. A 0.15 M solution of ferrioxalate was prepared by dissolving 1.8115 g of potassium ferrioxalate trihydrate in a 25.00 mL volumetric flask of 0.05 M H_2SO_4 in air. A buffered solution of phenanthroline was prepared by dissolving 518 mg of phenanthroline and 117.1 g of sodium acetate in 500.00 mL of 0.5 M H_2SO_4 in air. These two solutions were stored in the dark. Then, a series of ironvitriol solutions were prepared with different concentration gradients to react with the buffered solution of phenanthroline for 20 minutes. The absorbance of ferrous－phenanthroline compounds with different concentrations at 510 nm were measured. Thereby, we get the curve which can tell us how much ferrous is produced in the

potassium ferrioxalate photolysis reaction (Figure S4).

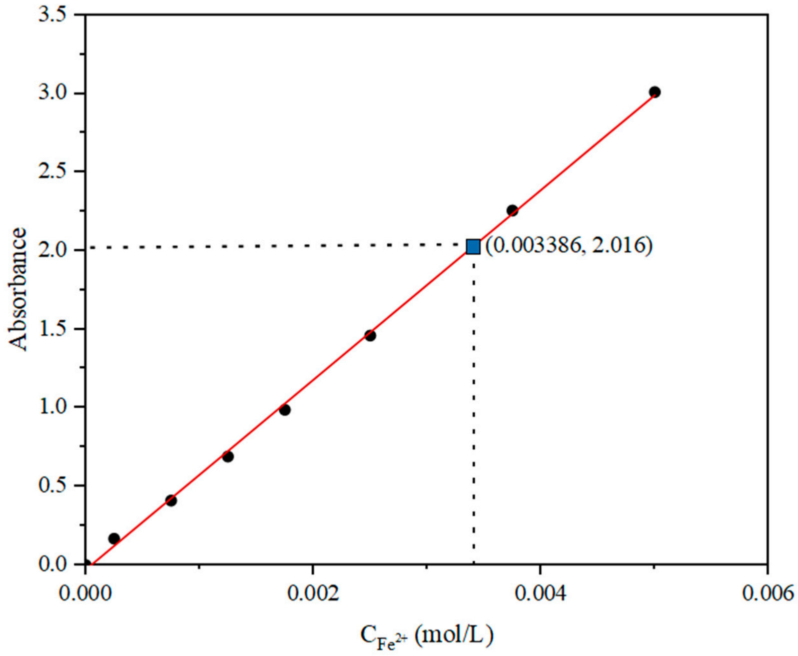

Figure S4. Data for [Fe^{2+}]–dependence on absorbance.

To determine the photon flux of the spectrophotometer, 5.00 mL of the ferrioxalate solution was placed in a quartz tube and irradiated for 600 seconds at 365 nm in our photoreactors. After irradiation, 15.00 mLof phenanthroline buffer solution was added to irradiated solution and then stirred for 20 min in the dark to allow the ferrous ions to completely coordinate to the phenanthroline. The absorbance of the solution was measured at 510 nm. A non–irradiated sample was also prepared and the absorbance at 510 nm measured. The concentration of ferrous ion was obtained by contrast curve (The dotted lines in Figure S1). We can know $\mathrm{n(Fe^{2+})} = 1.693 \times 10^{-5}\ \mathrm{mol}$ by simple calculation. The photon flux under the condition was calculated using eq (S1), where Φ is the quantum yield for the ferrioxalate actinometer (Φ = 1.13), t is the irradiation time (600 s), and f is the fraction of light absorbed at λ = 365 nm (f = 1 in this reaction). The photon flux was calculated to be 2.497 × 10^{-8} mol s^{-1}.

$$\text{photon flux} = \frac{\mathrm{n(Fe^{2+})}}{\Phi \times \mathrm{t} \times \mathrm{f}} \qquad \text{(S1)}$$

Sample calculation: $\text{photon flux} = \frac{\mathrm{n(Fe^{2+})}}{\Phi \times \mathrm{t} \times \mathrm{f}} = \frac{1.693 \times 10^{-5}\ \mathrm{mol}}{1.13 \times 600\ \mathrm{s} \times 1} = 2.497 \times 10^{-8}\ \mathrm{mol/s}$.

Determination of the reaction quantum yield for the benzyl alcohol photocatalytic oxidation: Benzyl alcohol (1 mmol, 0.108 g) and DCE (5 mL) were added to a 25 mL quartz tube,

a 1 MPa oxygen ball was placed at the mouth of the quartz tube, acetophenone (0.5 mmol, 0.0601 g) as an internal standard was added to the reaction solution after irradiation under a 365 nm ultraviolet lamp for 10 min, the amount of benzyl alcohol consumed was calculated according to the gas chromatography standard curve, and the quantum yield of benzyl alcohol photocatalytic oxidation reaction was determined by eq (S2), where n' is the amount of substance of raw materials that our photon reaction consumed in a given reaction time, t' is our reaction time. and the average value was taken for three parallel experiments. The reaction quantum yield was calculated to be 7.2 (± 2%), based on the average of three independent measurements.

$$\Phi = \frac{n'}{t' \times 2.497 \times 10^{-8}\ \mathrm{mol/s}} \qquad \text{(S2)}$$

Sample calculation: $\Phi_1 = \frac{n'}{t' \times 2.497 \times 10^{-8}\ \mathrm{mol/s}} = \frac{0.1075 \times 10^{-3}}{600 \times 2.497 \times 10^{-8}} = 7.18$

S5.6 Density functional theory (DFT) calculation

All calculations were performed using density functional theory with the hybrid functional B3LYP as implemented in the Gaussian 09 program and the 6–311G* basis set was used for all the atoms. Structural optimizations were performed without any symmetry constraints. Geometry optimizations were carried out with the def2–SVP basis set was used for all elements. Frequency calculations at the same level of theory were also performed to identify all stationary points as minima (zero imaginary frequency) or transition states (one imaginary frequency), and to obtain free energies at 323 K and 1 atm. Connections between the key transition–state structures and the corresponding reactants and products were confirmed using intrinsic reaction coordinate calculations. Using the Gaussian16 software package at the b3lyp/6–311++G* level of theory, the parameters such as E, Ecorr, G, ΔG, reaction coordinate energy of different reactant in the three paths were calculated by eq (S3) and eq (S4), where the subscript Re and Pr represent the reactant and product, respectively. E_{corr} is the thermal correction to free energy. *E* and *G* are the calculated total energy and Gibbs free energy, respectively. The results obtained are presented in Table S5, Table S6, Table S7. The cartesian coordinates for all optimized geometries at the B3LYP/6–311++G(d,p) level are shown in Table S8.

$$G = E + E_{\mathrm{corr}} \qquad \text{(S3)}$$

$\Delta G = G(\mathrm{Pr}) - G(\mathrm{Re})$ (S4)

Sample calculation:

$G(\mathrm{III}^*) = E(\mathrm{III}^*) + E_{\mathrm{corr}}(\mathrm{III}^*) = -345.5750 + 0.0795$ Hartree $= -345.4955$ Hartree

$\Delta G(\mathrm{III}^*) = G(\mathrm{Pr}) - G(\mathrm{Re}) = -345.4955 - (-345.6139)$ Hartree $= 0.1184$ Hartree

Table S5. The parameters of different reactant in path **A**.

Reactant	E (Hartree)	Ecorr (Hartre)	G (Hartree)	ΔG (Hartree)	ΔG (kcal/mol)	Reaction coordinate energy (kcal/mol)
III	–345.6934	0.0795	–345.6139	–	–	–
III^*	–345.5750	0.0795	–345.4955	0.1184	74.2896	74.2896
I	–346.9003	0.1009	–346.7994	–	–	–
II	–346.2607	0.0882	–346.1725	0.0592	37.1448	37.1448
O_2	–150.3093	–0.0152	–150.3245	–	–	–
IV	–496.6689	0.0934	–496.5755	–0.0785	–49.2512	–12.1064
$HOO^•$	–150.9589	–0.0082	–150.9670	–	–	–
III	–345.6934	0.0795	–345.6139	–0.0055	–3.3885	–15.4949

Table S6. The parameters of different reactant in path B.

Reactant	E (Hartree)	Ecorr (Hartre)	G (Hartree)	ΔG (Hartree)	ΔG (kcal/mol)	Reaction coordinate energy (kcal/mol)
I	–346.9003	0.1009	–346.7994	–	–	0
$HOO^•$	–150.9589	–0.0082	–150.9670	–	–	–
H_2O_2	–151.6034	0.0039	–151.5995	–	–	–
II	–346.2607	0.0882	–346.1725	–0.0055	–3.4507	–3.4507
O_2	–150.3093	–0.0152	–150.3245	–	–	–
IV	–496.6689	0.0934	–496.5755	–0.0785	–49.2312	–52.6819
H_2O_2	–151.6034	0.0039	–151.5995	–	–	–
III	–345.6934	0.0795	–345.6139	–0.0055	–3.4318	–56.1138

Table S7. The parameters of different reactant in path B.

Reactant	E (Hartree)	Ecorr (Hartre)	G (Hartree)	ΔG (Hartree)	ΔG (kcal/mol)	Reaction coordinate energy (kcal/mol)
II	–346.2607	0.0882	–346.1725	0	0	37.1448
O_2	–150.3093	–0.0152	–150.3245	–	–	–
IV	–496.6689	0.0934	–496.5755	–0.0785	–49.2312	–12.0865
I	–346.9003	0.1009	–346.7994	–	–	–
II	–346.2607	0.0882	–346.1725	–	–	–
V	–497.3116	0.1056	–497.2060	–0.0036	–2.2622	–14.3486
H_2O_2	–151.6034	0.0039	–151.5995	–	–	–
III	–345.6934	0.0795	–345.6139	–0.0074	–4.6204	–18.9690

Table S8. Cartesian coordinates for all optimized geometries at the B3LYP/6–311++G(d,p) level

I ($C_6H_5CH_2OH$)			
C	–2.30884400	–0.31589400	–0.03911100
C	–1.37157100	–1.34882200	0.03521900
C	–0.00481100	–1.06125000	0.08754700
C	0.43771200	0.26556600	0.06142000
C	–0.50569500	1.29748600	–0.02281100
C	–1.87115600	1.01143300	–0.06777700
H	–3.37103700	–0.54185100	–0.08013900
H	–1.70354200	–2.38376000	0.05113900
H	0.72623100	–1.86091100	0.13748400
H	–0.17038500	2.33249200	–0.05656100
H	–2.59148900	1.82261800	–0.13410100
C	1.90574600	0.60581200	0.16432900
H	2.14581300	0.87871000	1.20470300
H	2.12536700	1.47996600	–0.46665600
O	2.69095800	–0.51615600	–0.23867600
H	3.62309700	–0.32401100	–0.05936000

II (C6H5CH$^{\bullet}$OH)			
C	2.27935100	–0.30604600	–0.00027700
C	1.34082100	–1.34874000	0.00002200
C	–0.02185800	–1.08199000	0.00025800
C	–0.49344400	0.26255800	0.00005800
C	0.47283600	1.31004300	0.00024000
C	1.82962100	1.02441400	–0.00002100
H	3.34320200	–0.52477500	–0.00051800
H	1.68192900	–2.38108400	0.00007500
H	–0.74272700	–1.89258500	0.00064000
H	0.13391000	2.34368600	0.00070000
H	2.54903500	1.83938400	–0.00002800
C	–1.86510200	0.57252700	–0.00026700
H	–2.23458500	1.59343000	–0.00024800
O	–2.7876940	–0.43806400	–0.00009600
H	–3.68256500	–0.07013600	0.00007100

III (C6H5CHO)			
C	–2.21997700	–0.24446000	–0.00007000
C	–1.33332200	–1.32955700	–0.00012500
C	0.04060500	–1.10676100	0.00020400
C	0.53496100	0.20744500	0.00028900
C	–0.35574000	1.29003300	–0.00002600
C	–1.73273700	1.06558200	–0.00008500
H	–3.29208300	–0.42222100	0.00031200
H	–1.71928900	–2.34514400	–0.00025900
H	0.74724800	–1.93106000	0.00012400
H	0.03493000	2.30555000	–0.00028600

H	–2.42279300	1.90454300	0.00004200
C	1.99092800	0.46670200	0.00001500
H	2.27317300	1.54182600	0.00025000
O	2.85381400	–0.39342400	–0.00017500

IV ($C6H5COO^{\bullet}OH$)			
C	–2.94703900	0.05401400	–0.27451200
C	–2.15159000	1.19397700	–0.42856100
C	–0.78401100	1.13642400	–0.16104500
C	–0.20828900	–0.06570300	0.26682700
C	–1.00455100	–1.20501300	0.42099200
C	–2.37282700	–1.14653300	0.15006400
H	–4.01237600	0.10265500	–0.48287000
H	–2.59819000	2.12835100	–0.75689800
H	–0.15943700	2.01648400	–0.27172100
H	–0.55494900	–2.13763700	0.75343800
H	–2.98792600	–2.03332200	0.27367100
C	1.26252600	–0.15690500	0.51777900
H	1.53578400	–1.01763200	1.14016500
O	1.79249700	1.02650400	0.97865700
H	2.76257000	0.92771900	0.97870900
O	1.90137300	–0.49544200	–0.82146500
O	3.21228100	–0.38708500	–0.73016300

V (C6H5COOHOH)			
C	3.00448100	0.12619400	–0.22149300
C	2.29740600	1.25917800	0.19202200
C	0.92368000	1.17718000	0.42073700
C	0.25015800	–0.03569600	0.23610000
C	0.95655600	–1.16763800	–0.18044400
C	2.33247400	–1.08480500	–0.40574300
H	4.07529600	0.18715600	–0.39666200
H	2.81510300	2.20325300	0.33780900
H	0.36912400	2.05654800	0.73816800
H	0.42776500	–2.10448700	–0.31509100
H	2.87980500	–1.96773900	–0.72467000
C	–1.23901700	–0.08729600	0.47092900
O	–1.81062200	0.60053300	–0.64846400
O	–1.68587300	–1.41102600	0.55221500
H	–2.63725200	–1.38033800	0.74150900
O	–3.24763300	0.69064800	–0.35629800
H	–3.60229200	0.36991400	–1.20458200

$HOO^{\bullet}$			
H	–0.89249600	–0.87385100	0.00000000
O	0.05578100	–0.61182600	0.00000000
O	0.05578100	0.72105800	0.00000000

H_2O_2			
H	1.02340800	–0.66478300	0.43801300
O	0.71884500	0.11830500	–0.05475400
O	–0.71884500	–0.11830400	–0.05475100
H	–1.02340600	0.66477800	0.43802500

O_2			
O	0.00000000	0.00000000	0.60760700
O	0.00000000	0.00000000	–0.60760700

S6 NMR spectra

Benzoic acid (1c)

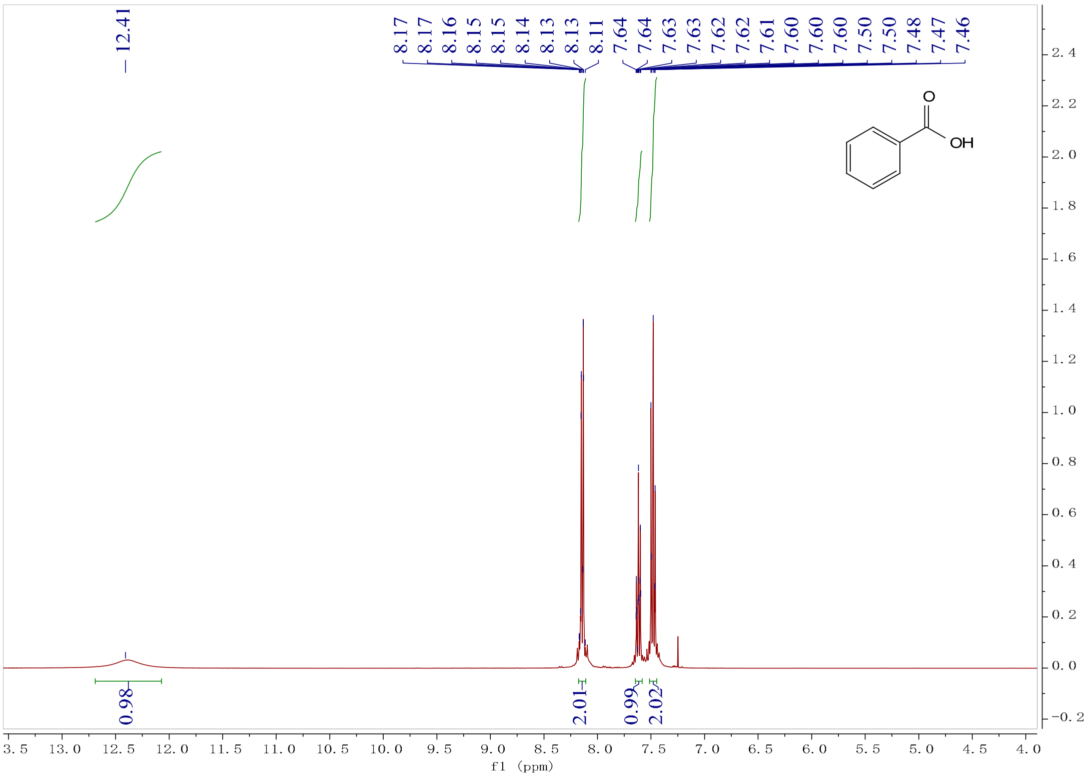

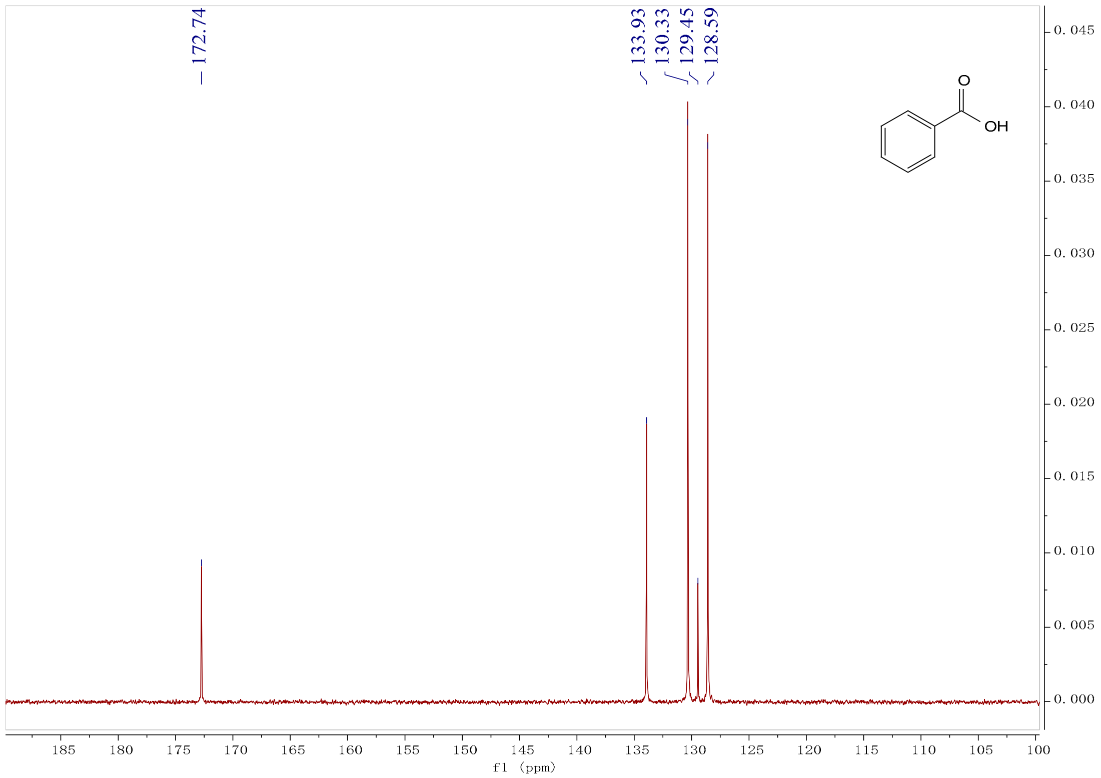

Figure S5. ^{1}H **(400 MHz) and** ^{13}C **{1H} (101 MHz) NMR spectra of 1c in** $CDCl_3$**.**

4–Methylbenzoic acid (2c)

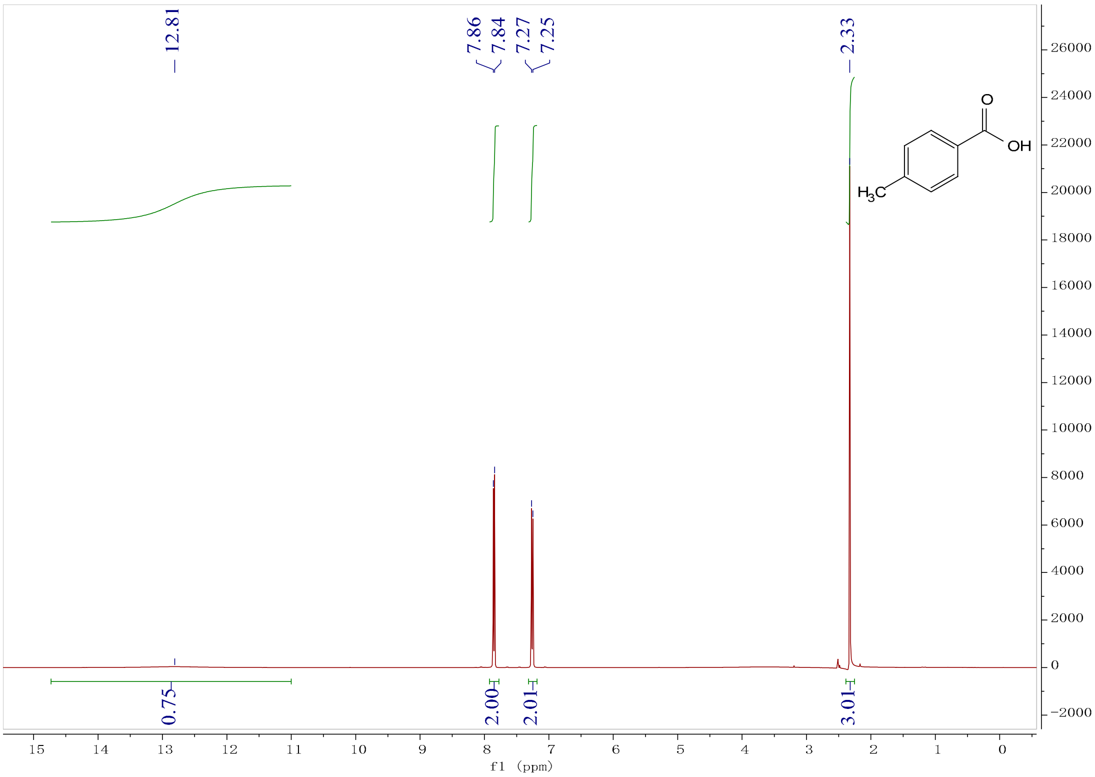

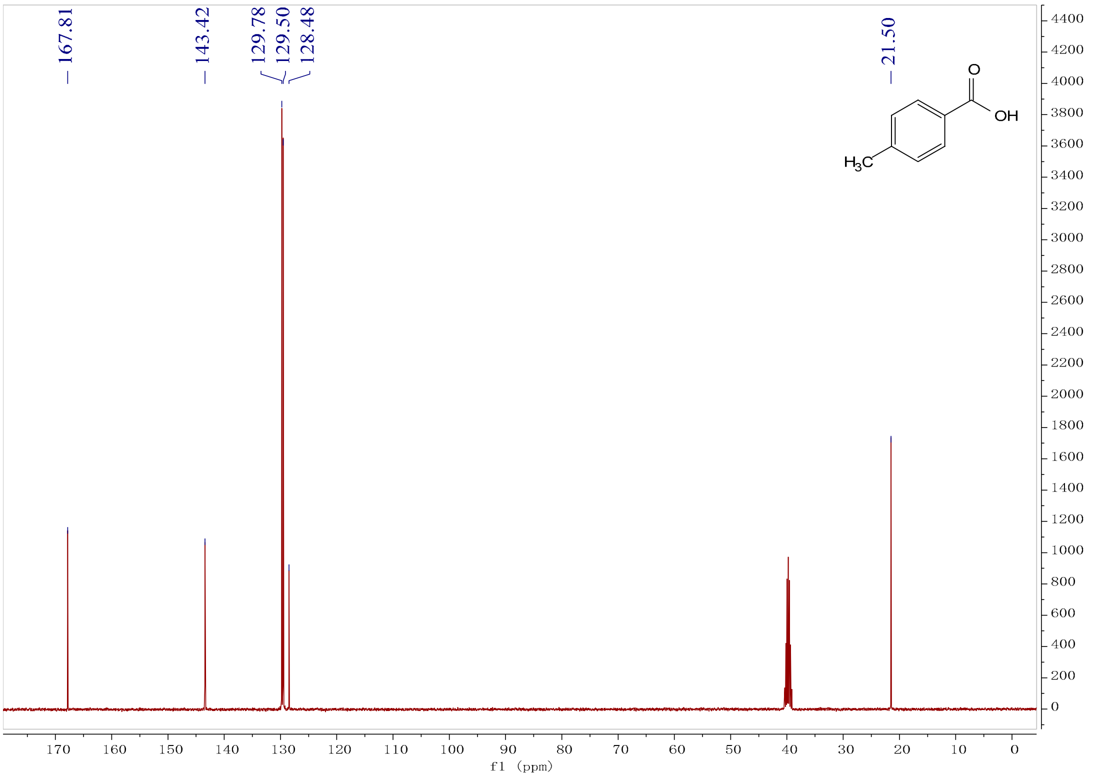

Figure S6. ^{1}**H (400 MHz) and** ^{13}**C {1H} (101 MHz) NMR spectra of 2c in DMSO–*d*$_6$.**

3–Methylbenzoic acid (3c)

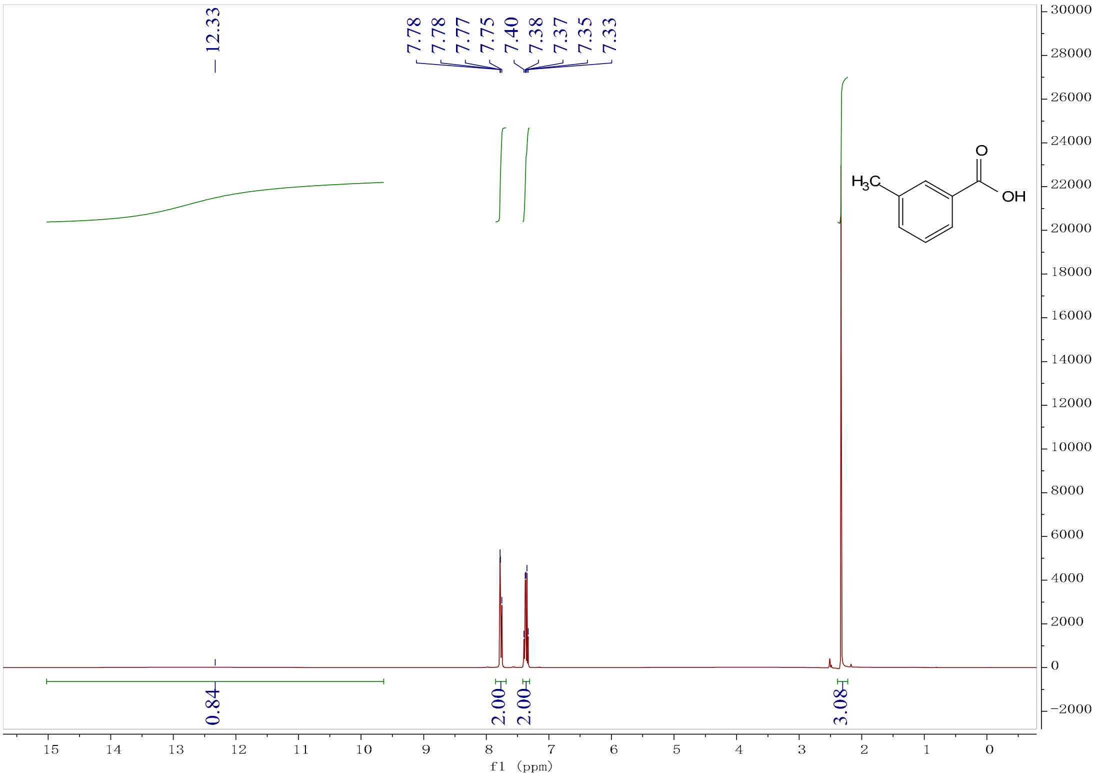

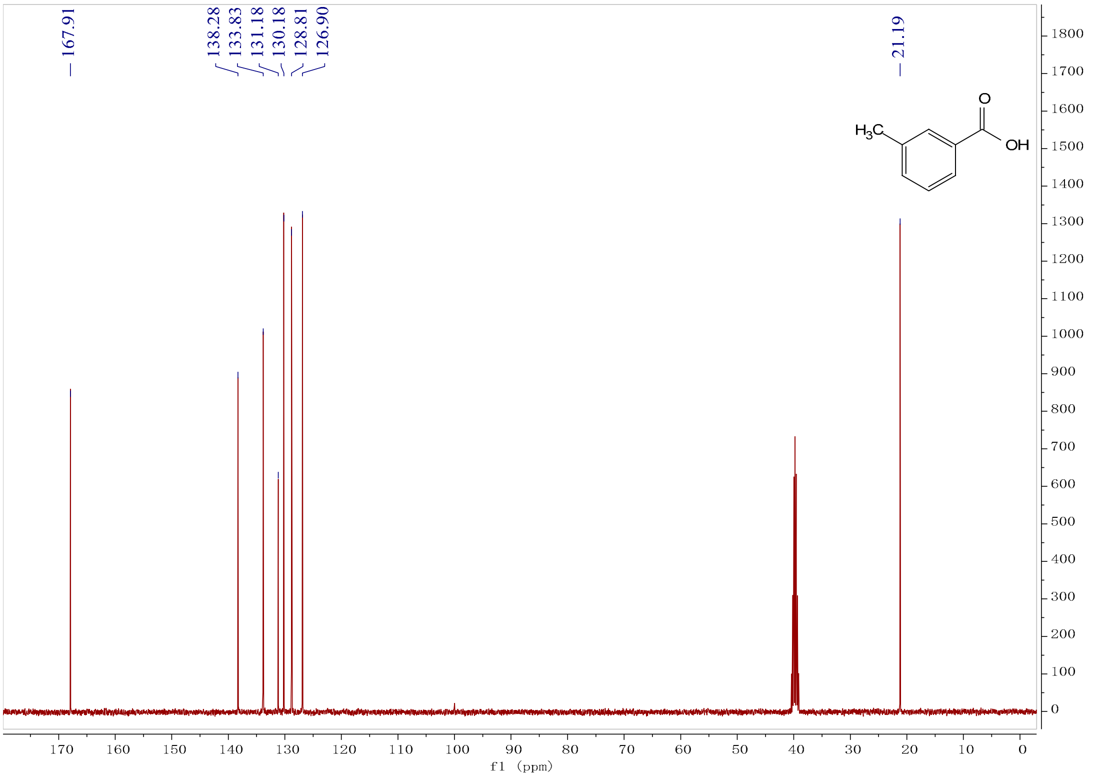

Figure S7. ^{1}H (400 MHz) and ^{13}C {1H} (101 MHz) NMR spectra of 3c in DMSO–*d_6*.

Biphenyl–4–carboxylic acid (4c)

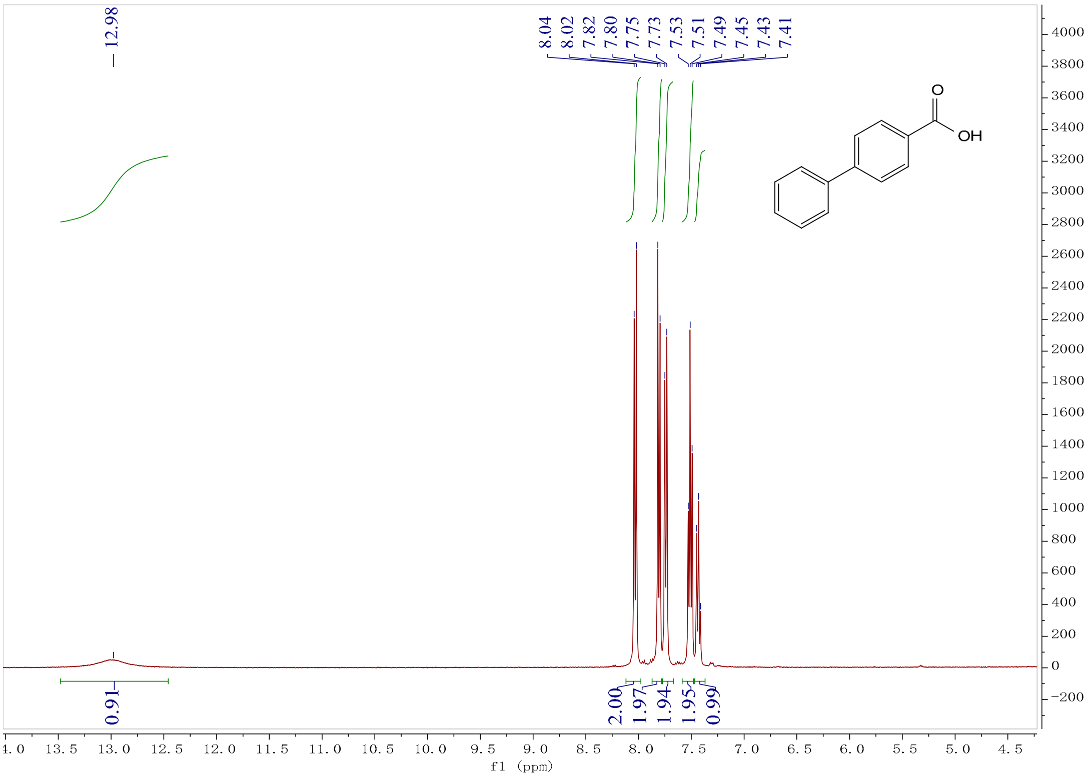

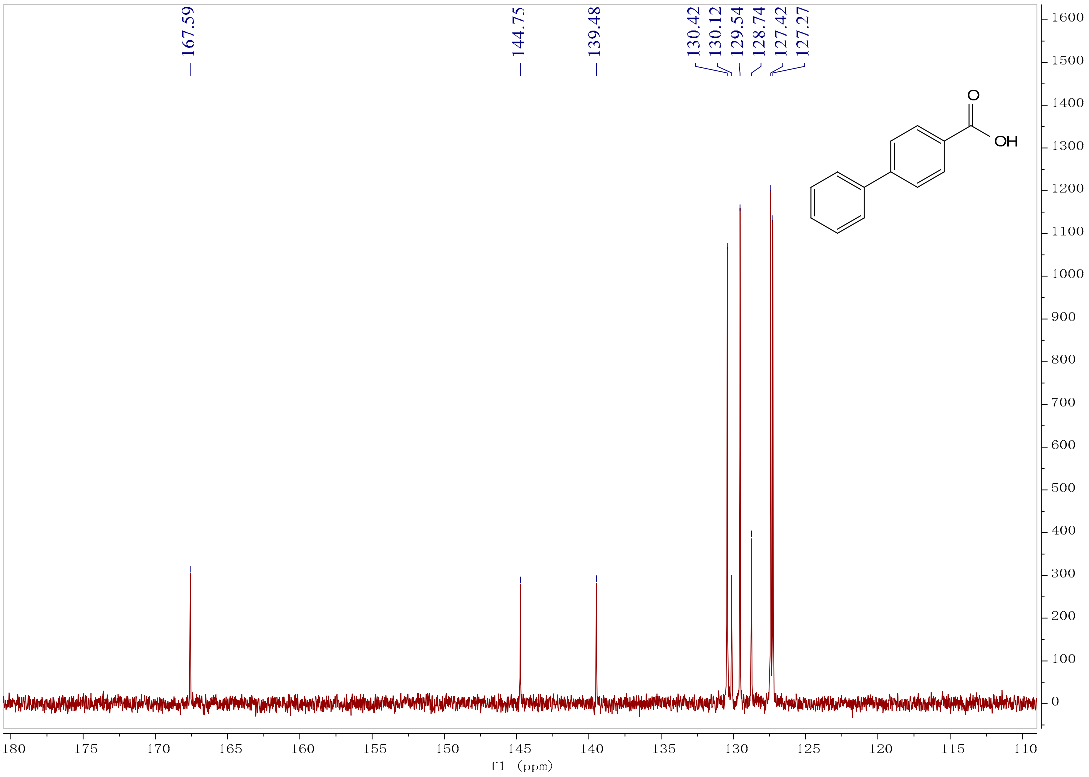

Figure S8. **^{1}H (400 MHz) and ^{13}C {1H} (101 MHz) NMR spectra of 4c in DMSO–*d*$_{6}$.**

4–tert–Butylbenzoic acid *(5c)*

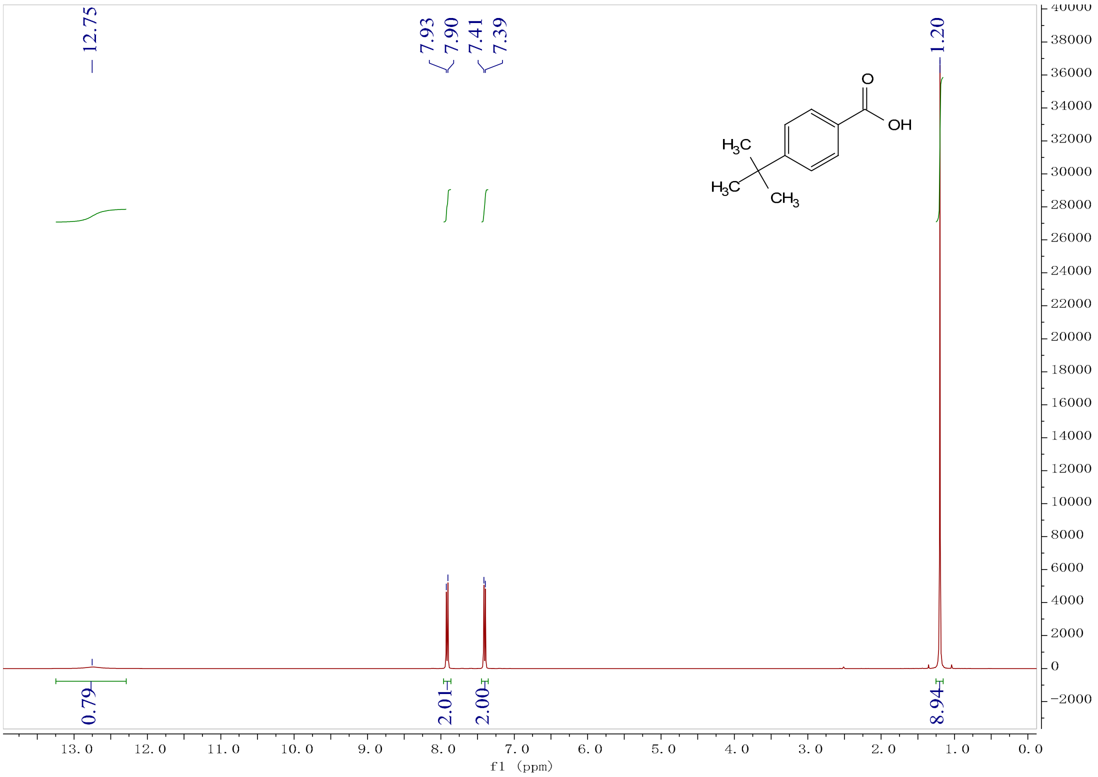

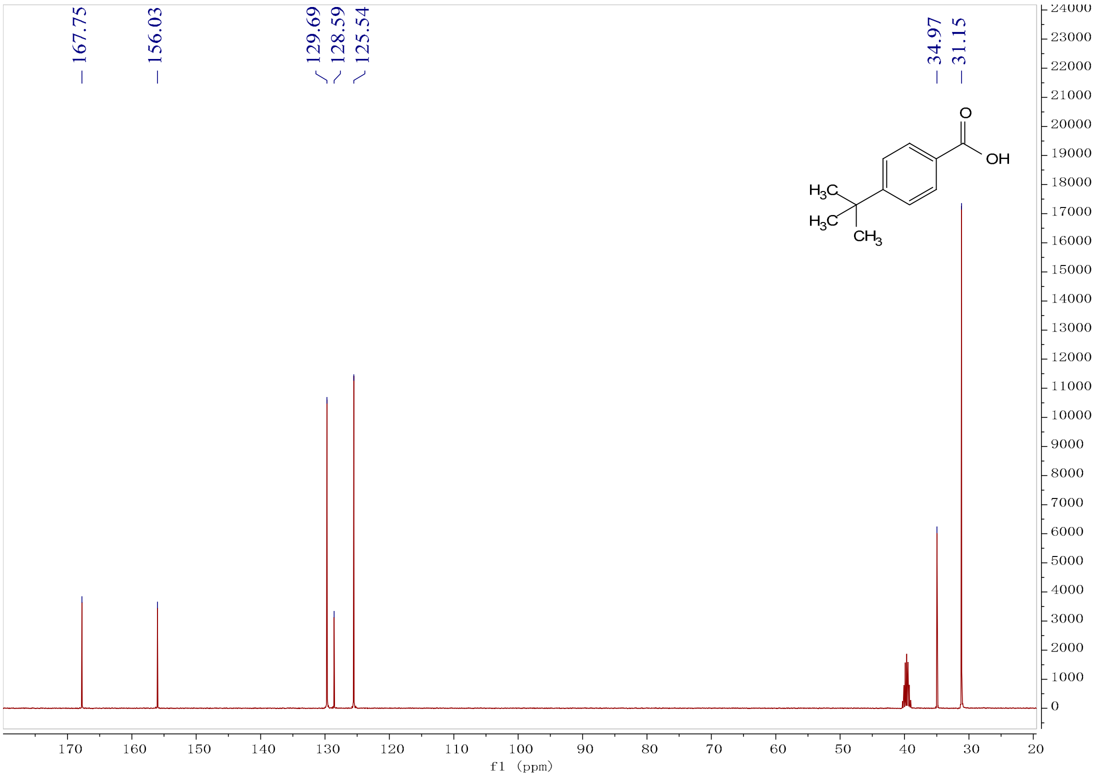

Figure S9. ^{1}**H (400 MHz) and** ^{13}**C {1H} (101 MHz) NMR spectra of 5c in DMSO–*d*$_6$.**

4–Methoxybenzoic acid (6c)

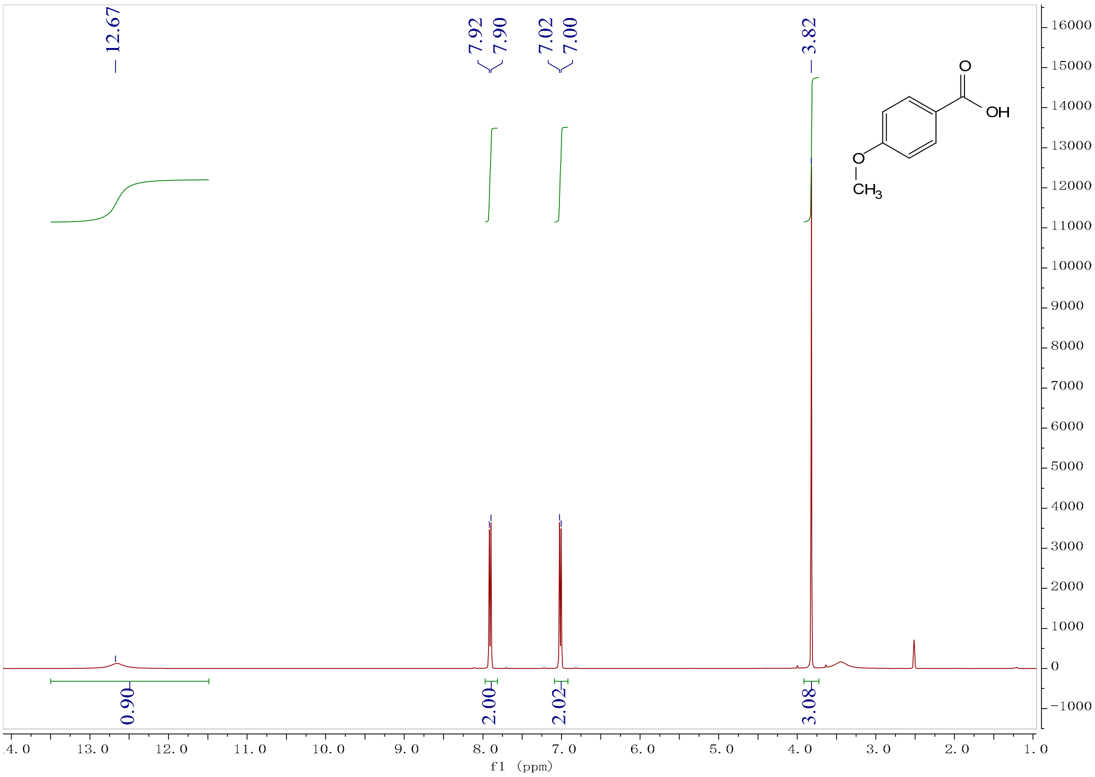

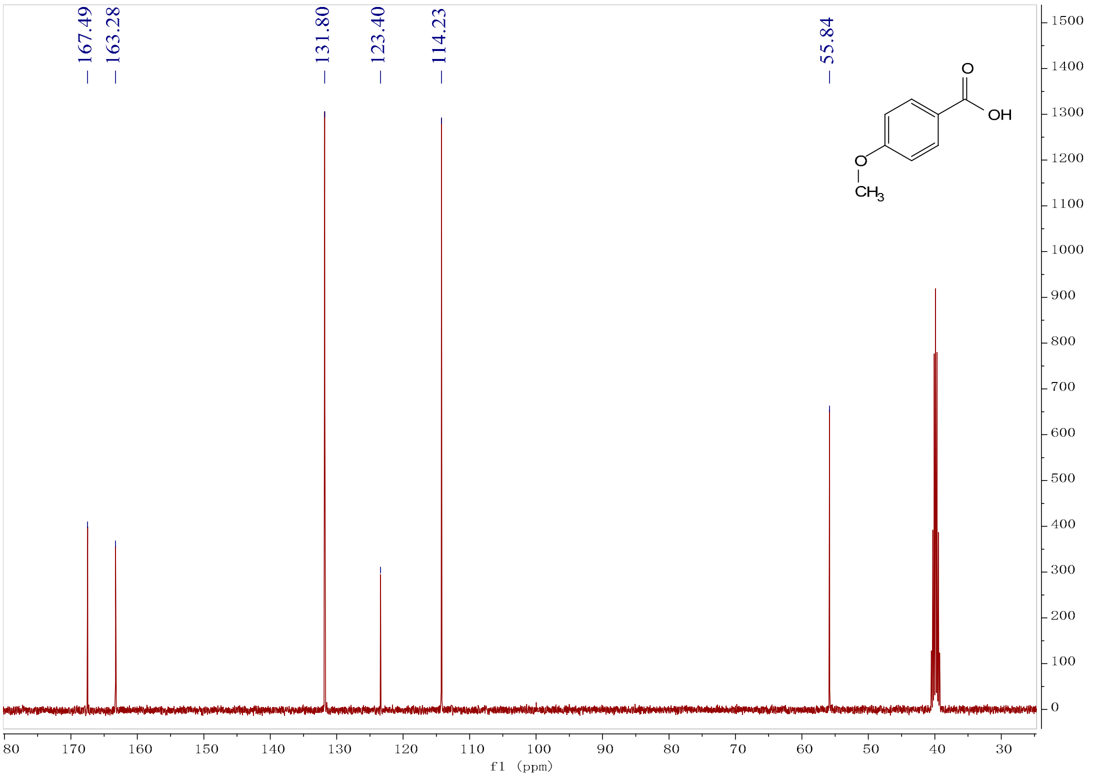

Figure S10. ^{1}**H (400 MHz) and** ^{13}**C {1H} (101 MHz) NMR spectra of 6c in DMSO–*d*$_6$.**

4–Phenoxybenzoic acid (7c)

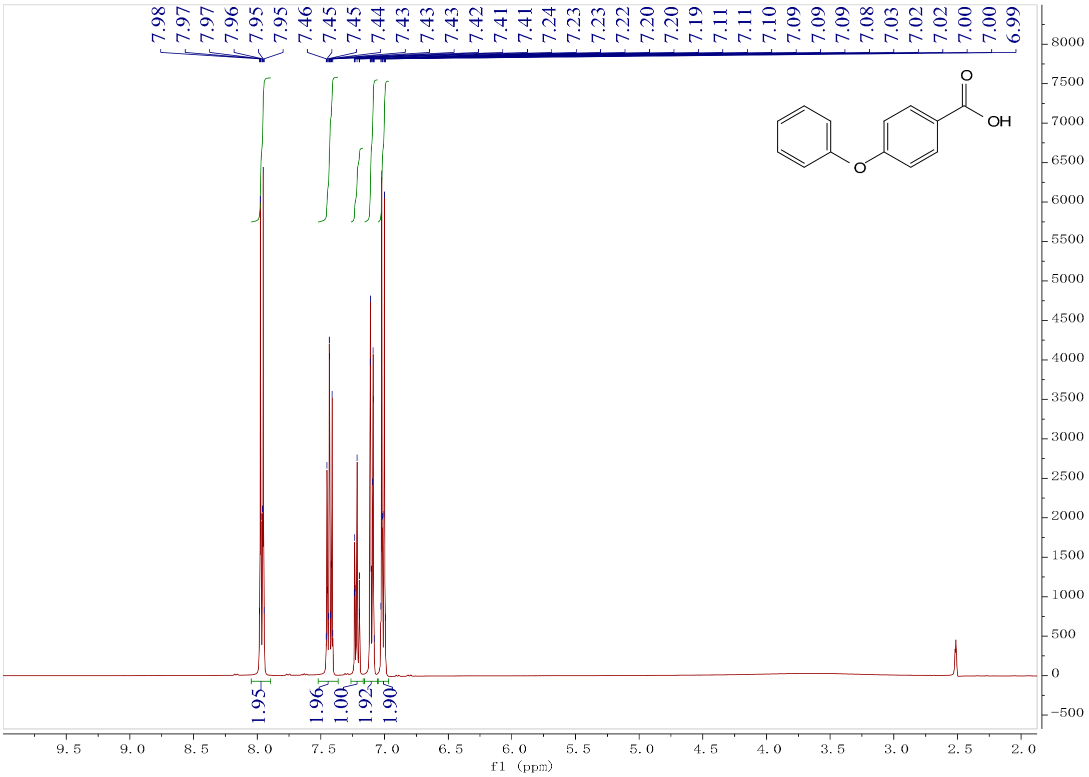

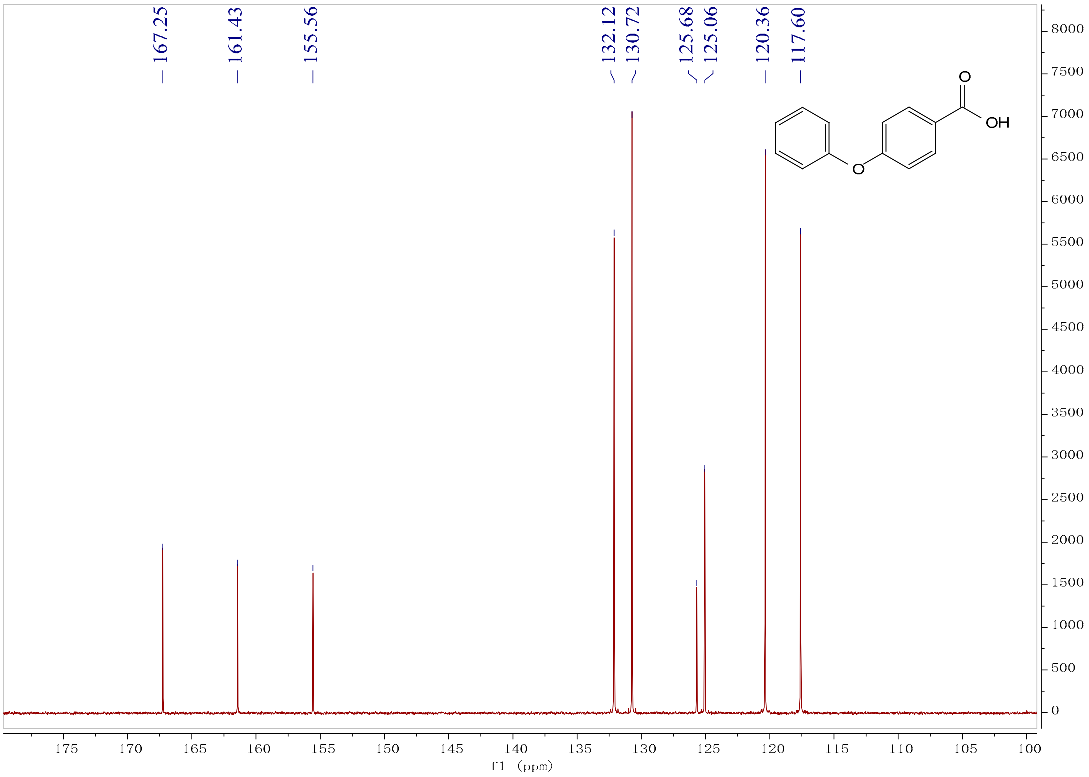

Figure S11. ^{1}H (400 MHz) and ^{13}C {1H} (101 MHz) NMR spectra of 7c in DMSO–d_6.

4–Acetyloxybenzoic acid (8c)

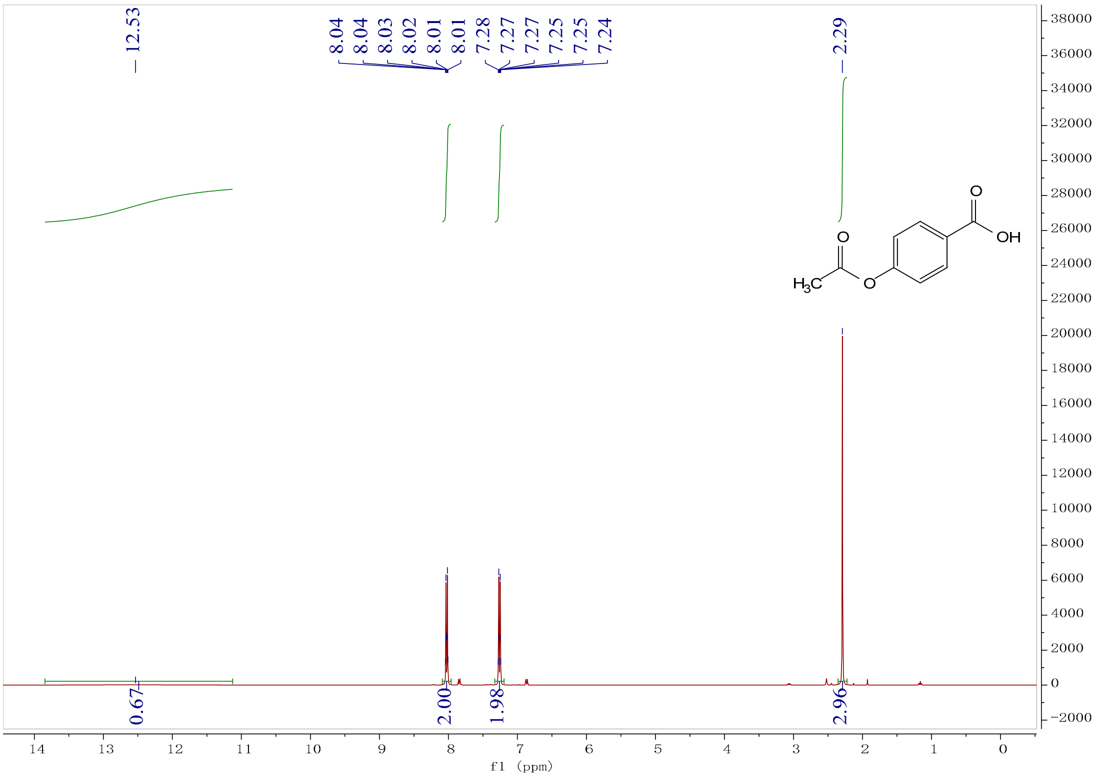

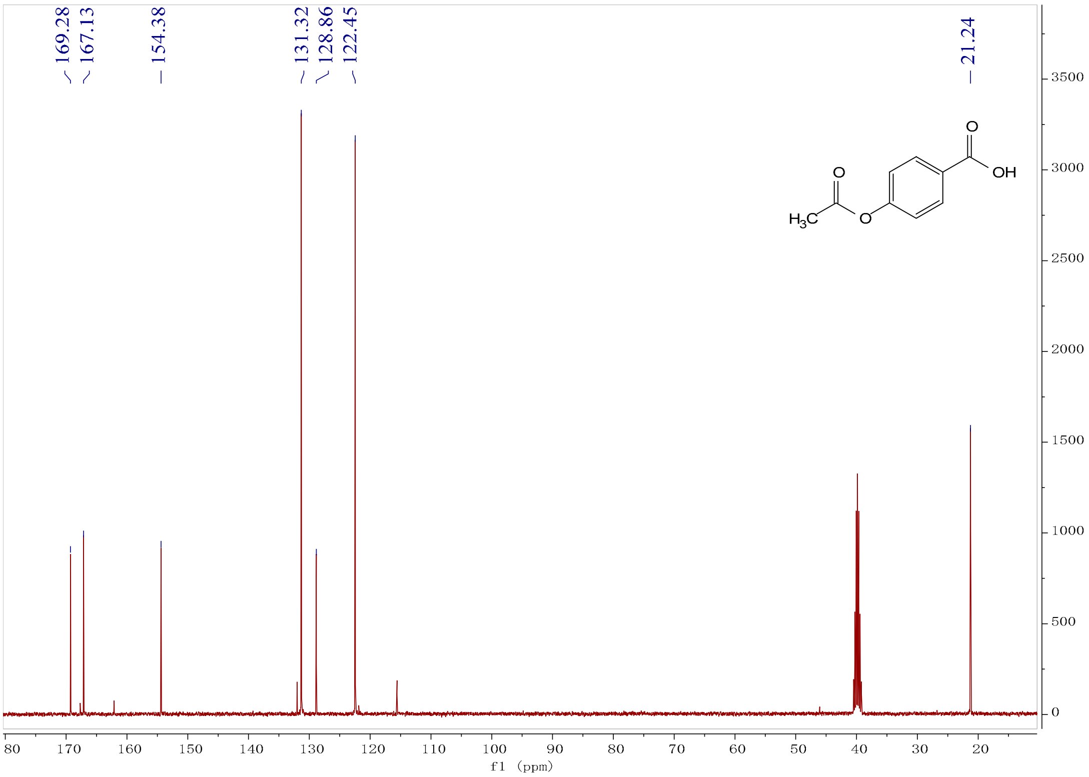

Figure S12. ^{1}**H (400 MHz) and** ^{13}**C {1H} (101 MHz) NMR spectra of 8c in DMSO–*d*$_6$.**

4–Acetamido benzoic acid (9c)

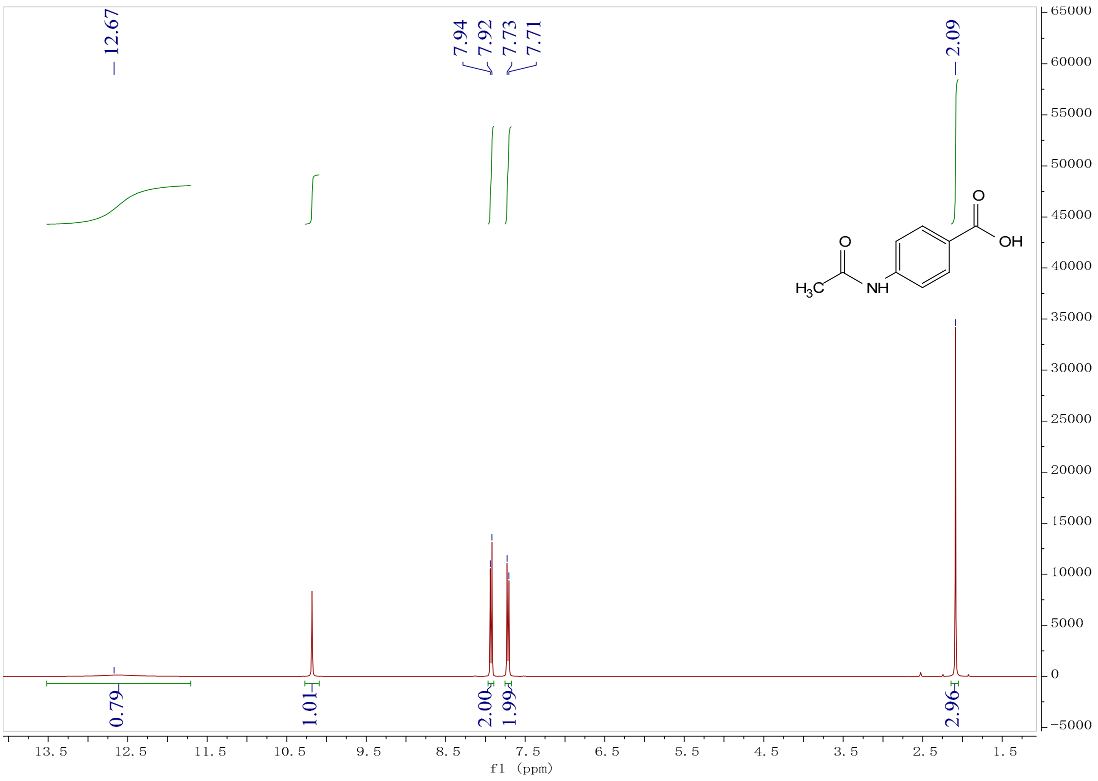

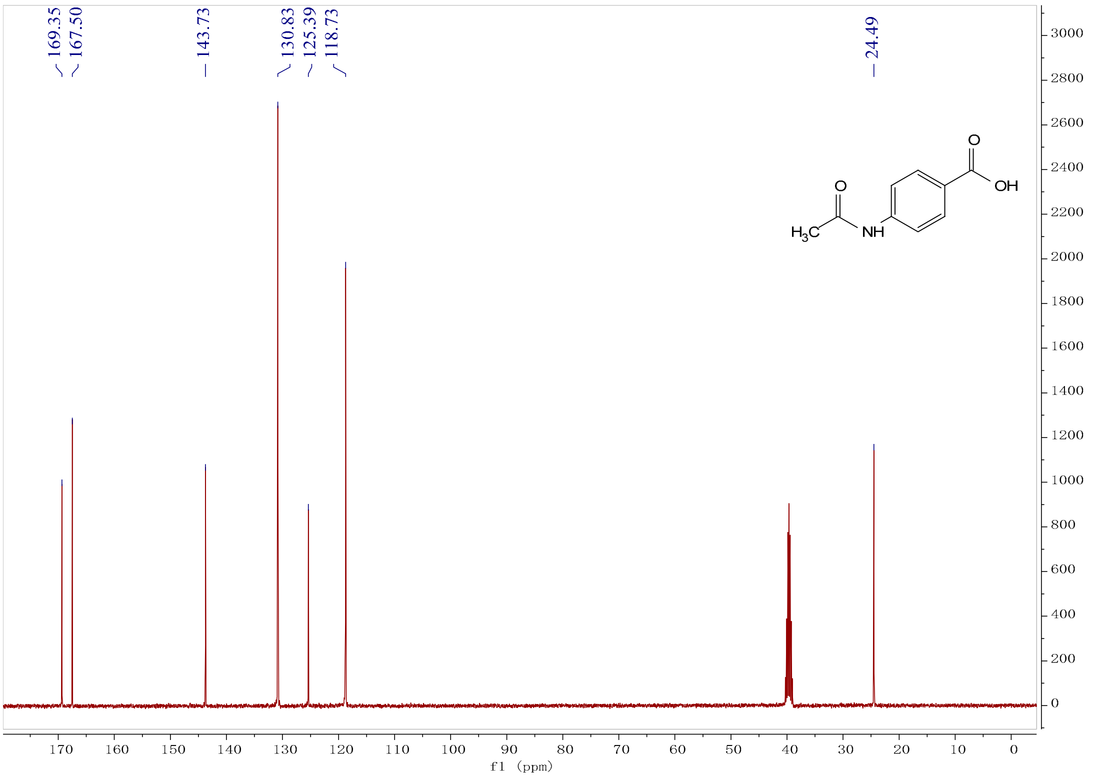

Figure S13. **^{1}H (400 MHz) and ^{13}C {1H} (101 MHz) NMR spectra of 9c in DMSO–*d*$_6$.**

4–Fluorobenzoic acid (10c)

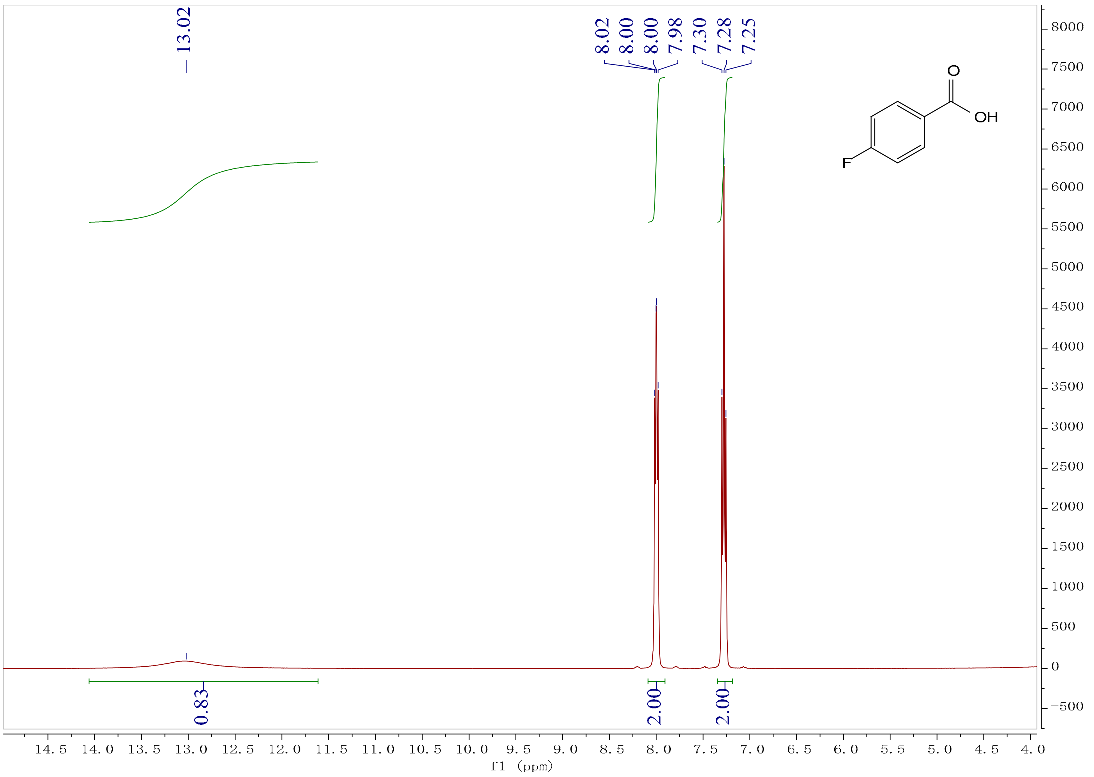

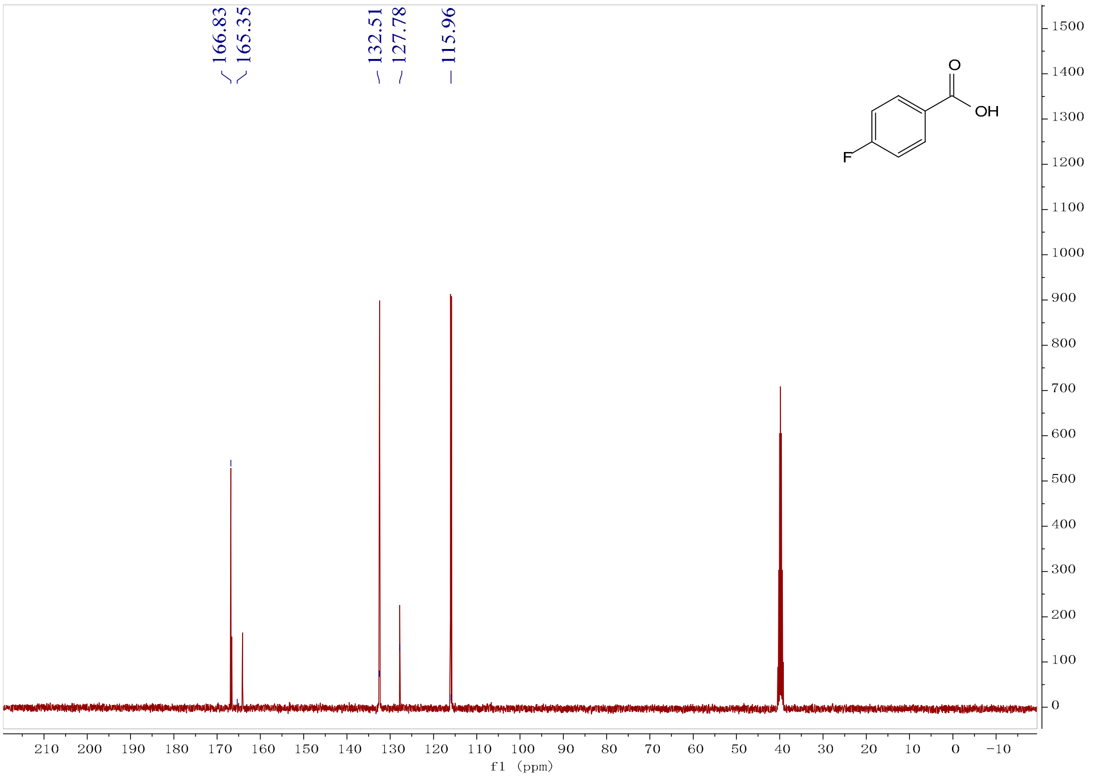

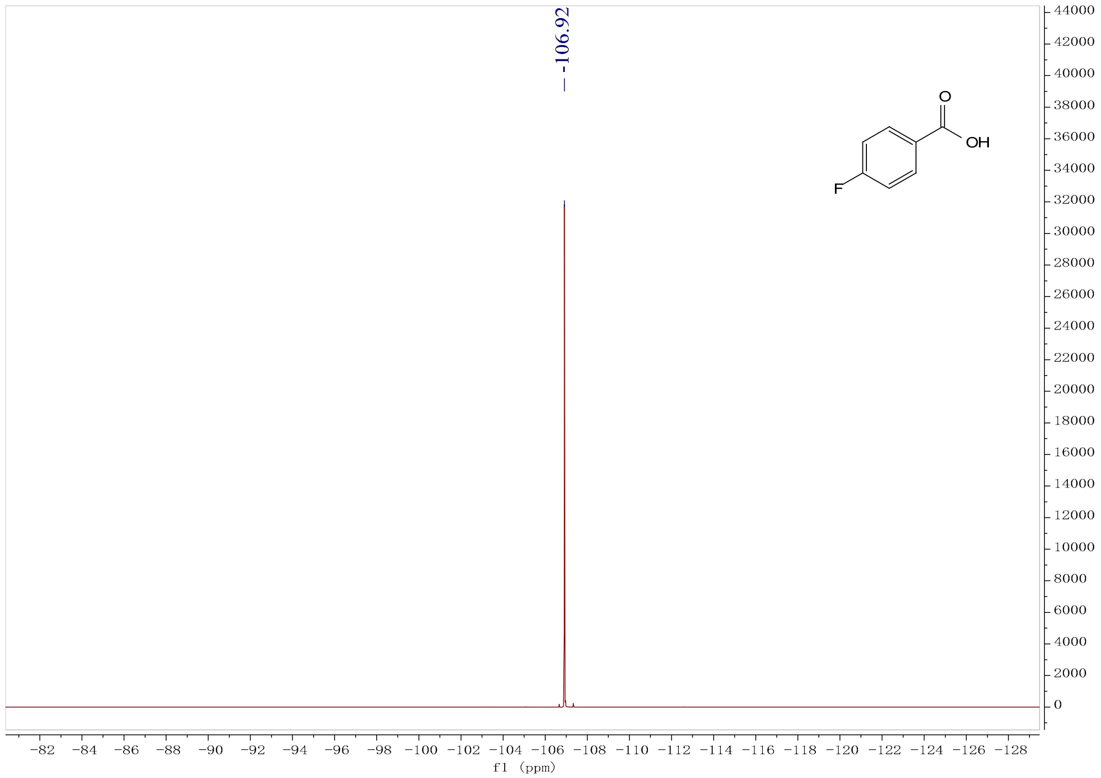

Figure S14. ^{1}H **(400 MHz),**^{13}C **{1H} (101 MHz) and** ^{19}F **{1H} (376 MHz) NMR spectra of 10c in DMSO–*d*$_6$.**

4–Chlorobenzoic acid (11c)

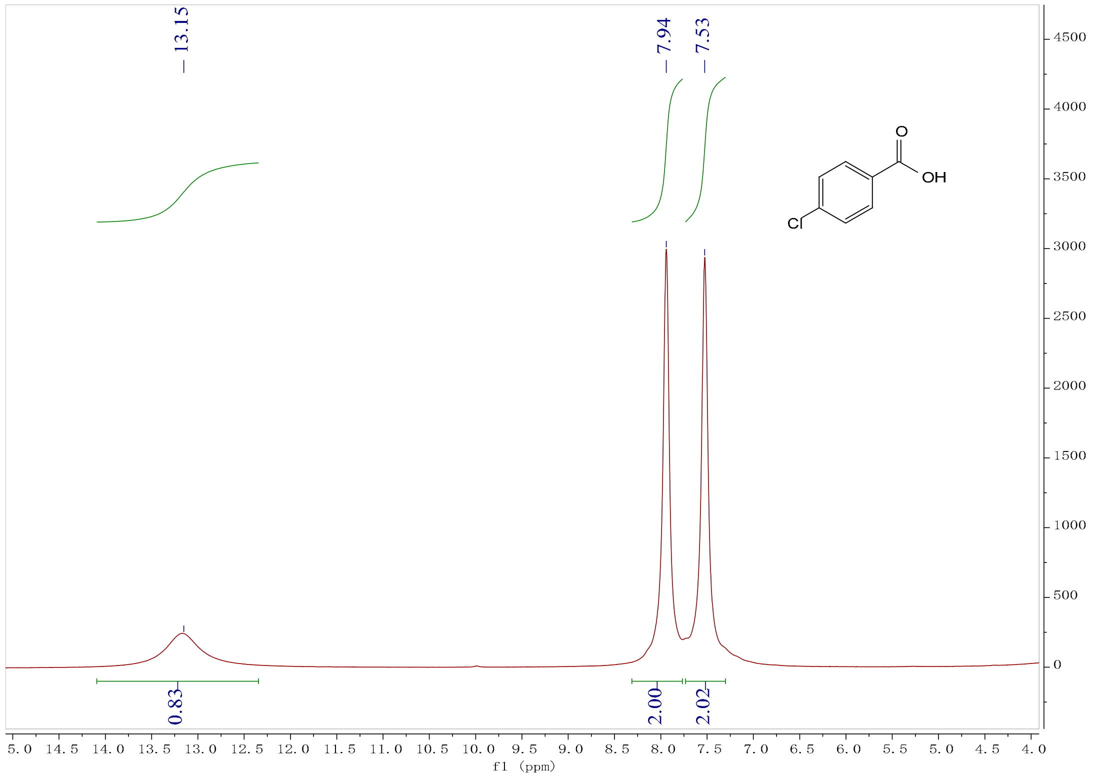

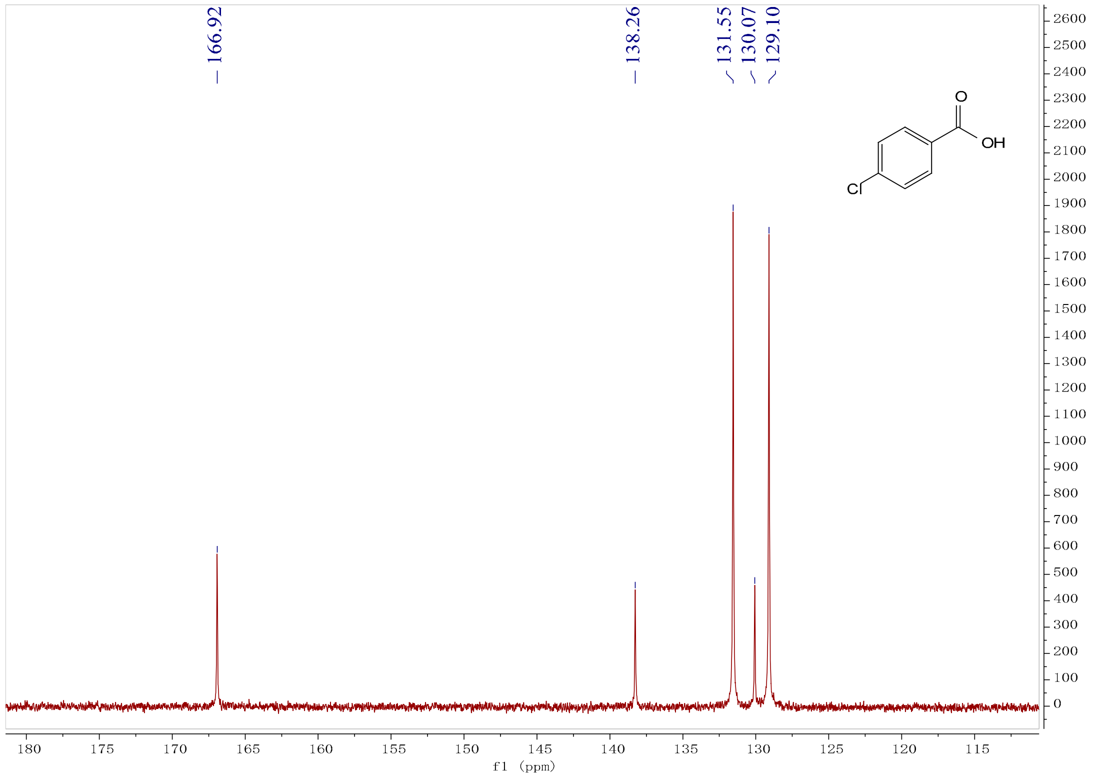

Figure S15. ^{1}H (400 MHz) and ^{13}C {1H} (101 MHz) NMR spectra of 11c in DMSO–*d*$_6$.

4–Bromobenzoic acid (12c)

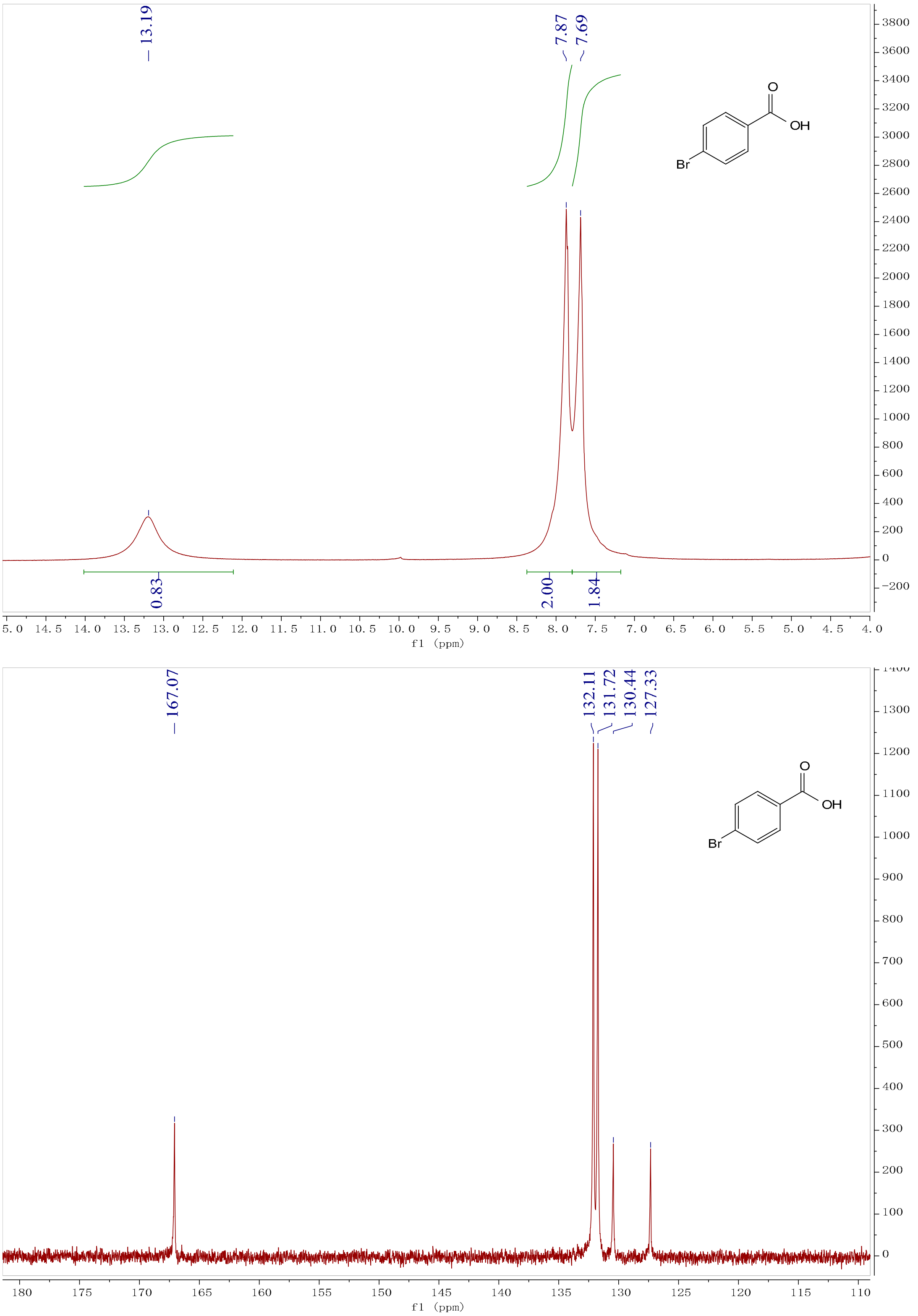

Figure S16. **^{1}H (400 MHz) and ^{13}C {1H} (101 MHz) NMR spectra of 12c in DMSO–*d_6*.**

2,4–Dichlorobenzoic acid (13c)

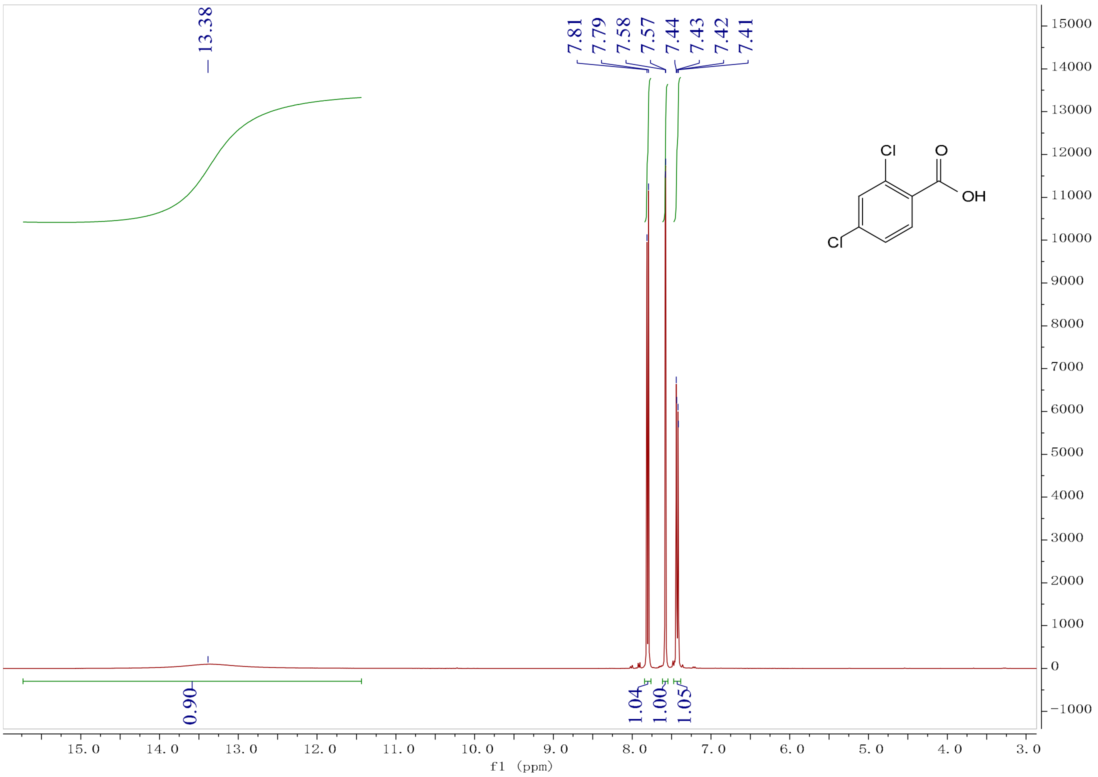

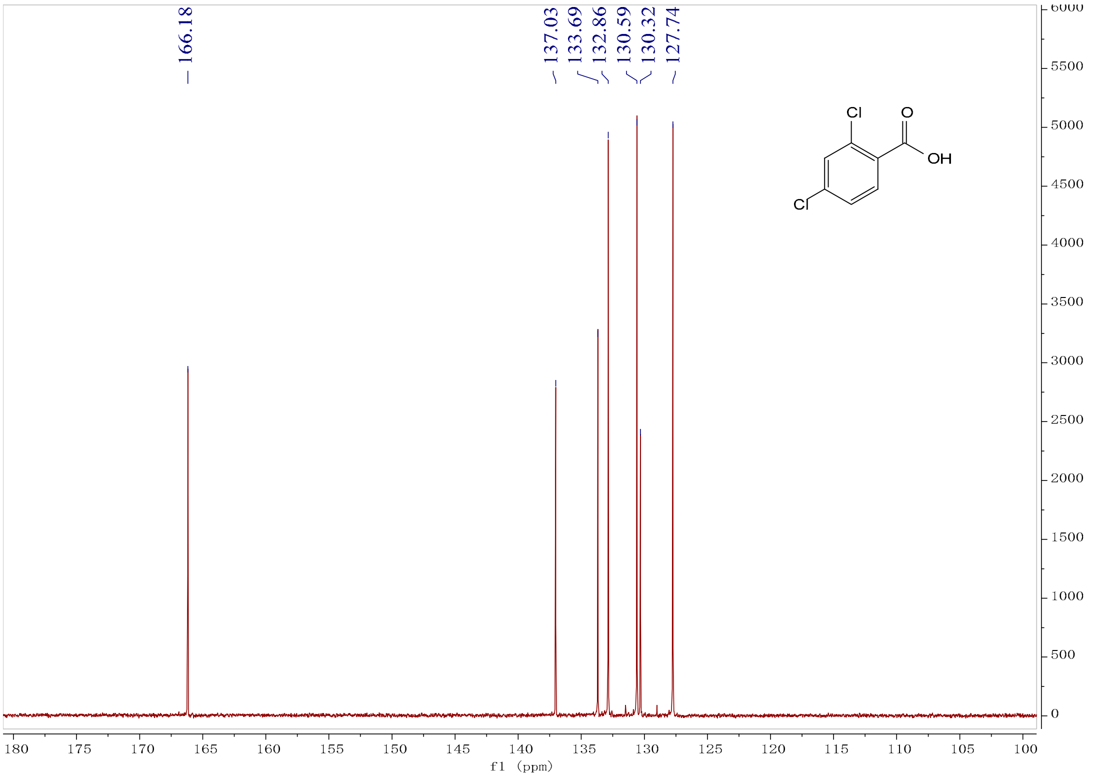

Figure S17. ^{1}H (400 MHz) and ^{13}C {1H} (101 MHz) NMR spectra of 13c in DMSO–*d_6*.

3,4–Dichlorobenzoic acid (14c)

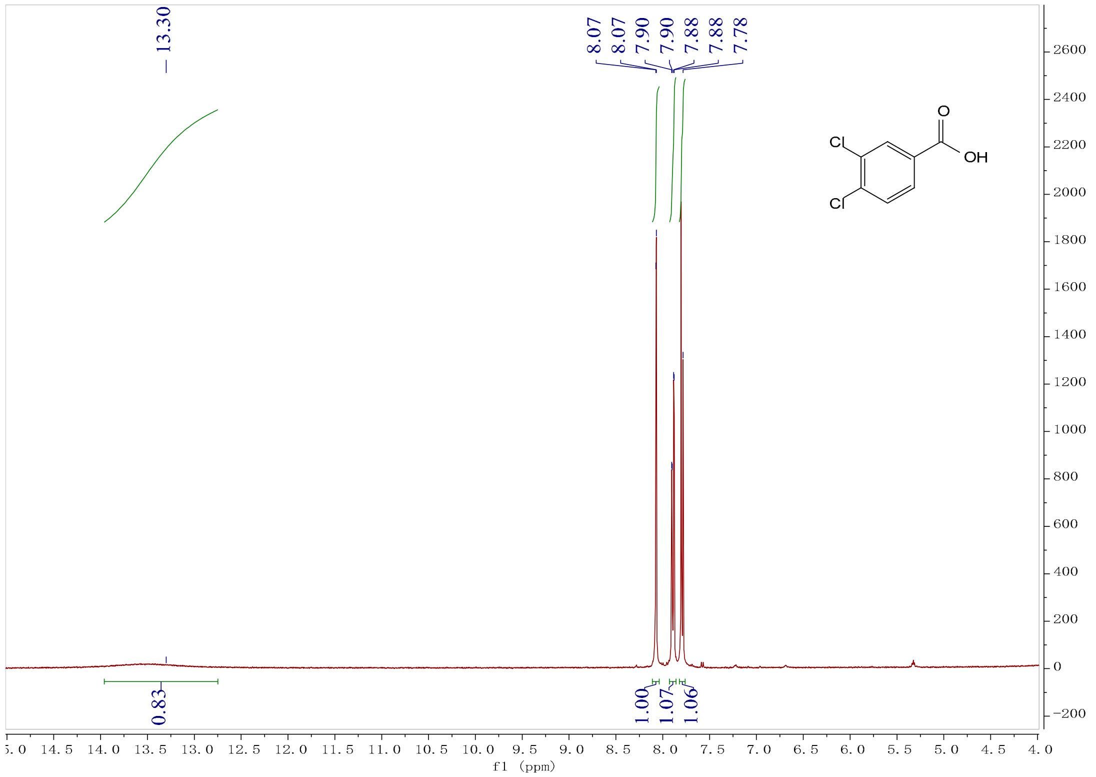

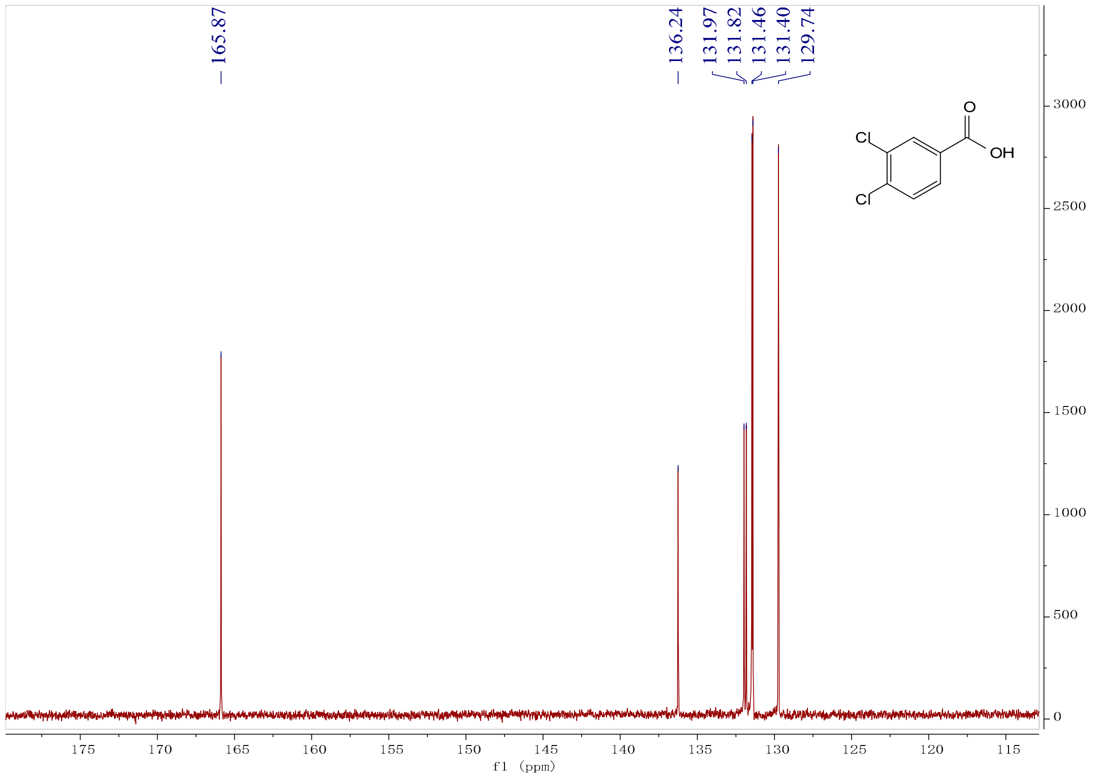

Figure S18. ^{1}H (400 MHz) and ^{13}C {1H} (101 MHz) NMR spectra of 14c in DMSO–*d*$_6$.

2–Chloro–4–fluorobenzoic acid (15c)

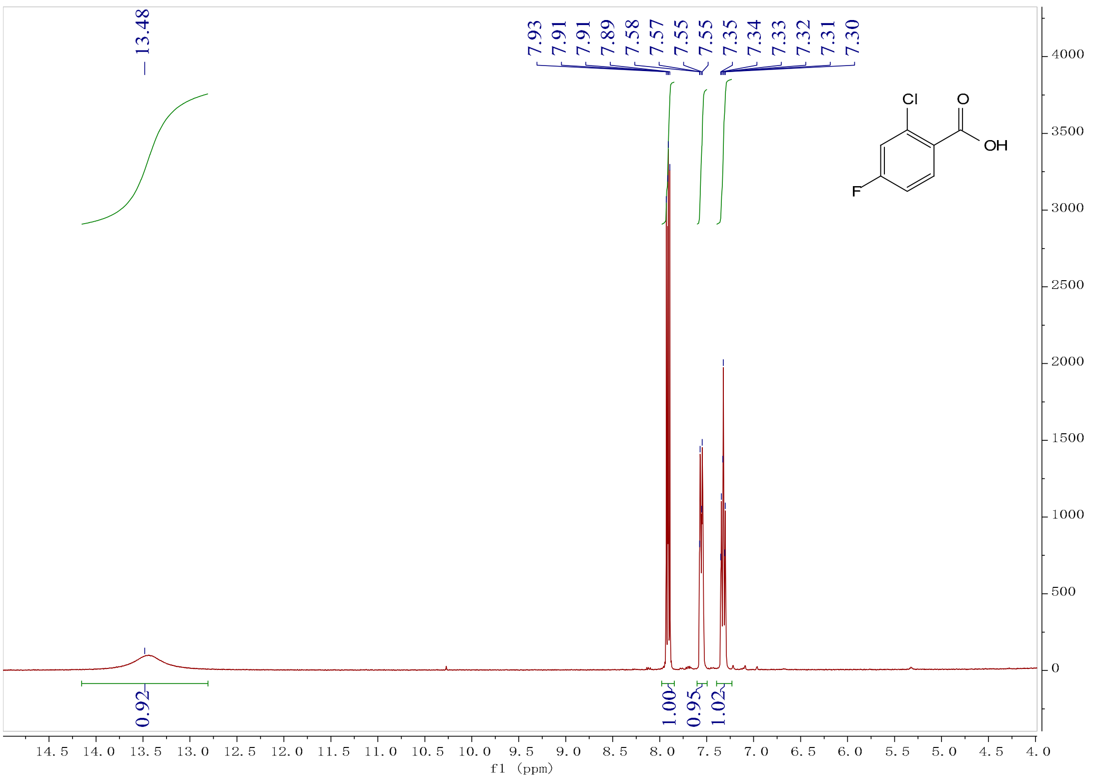

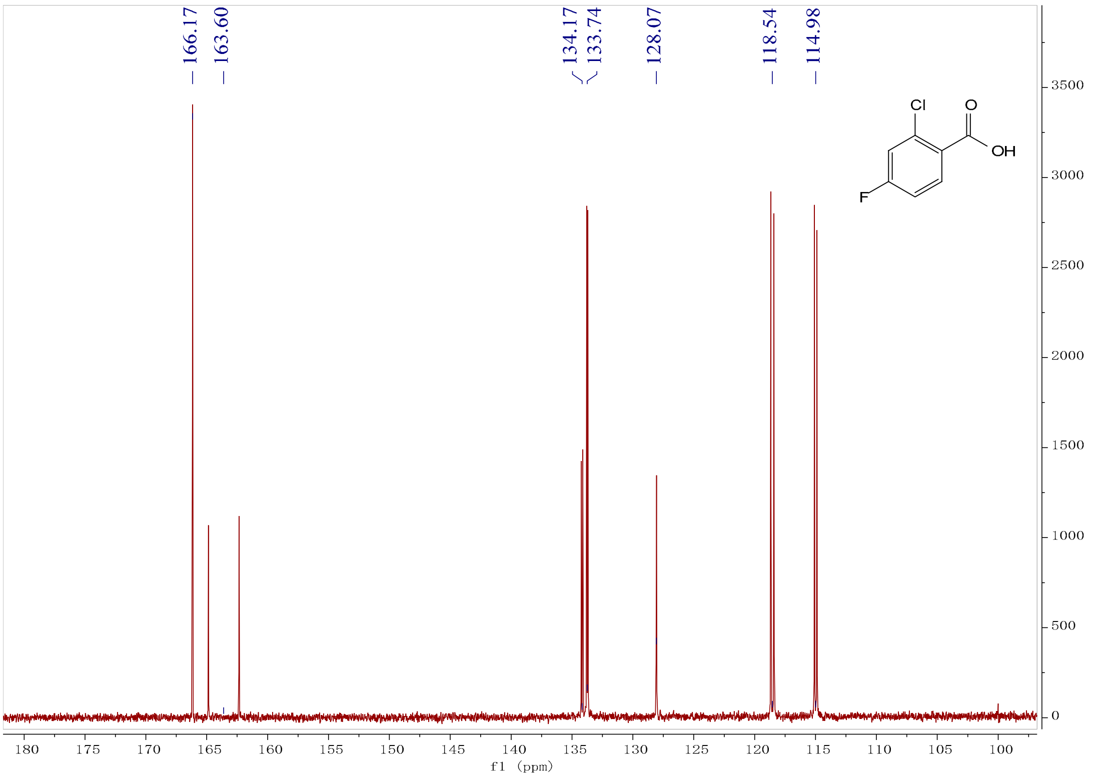

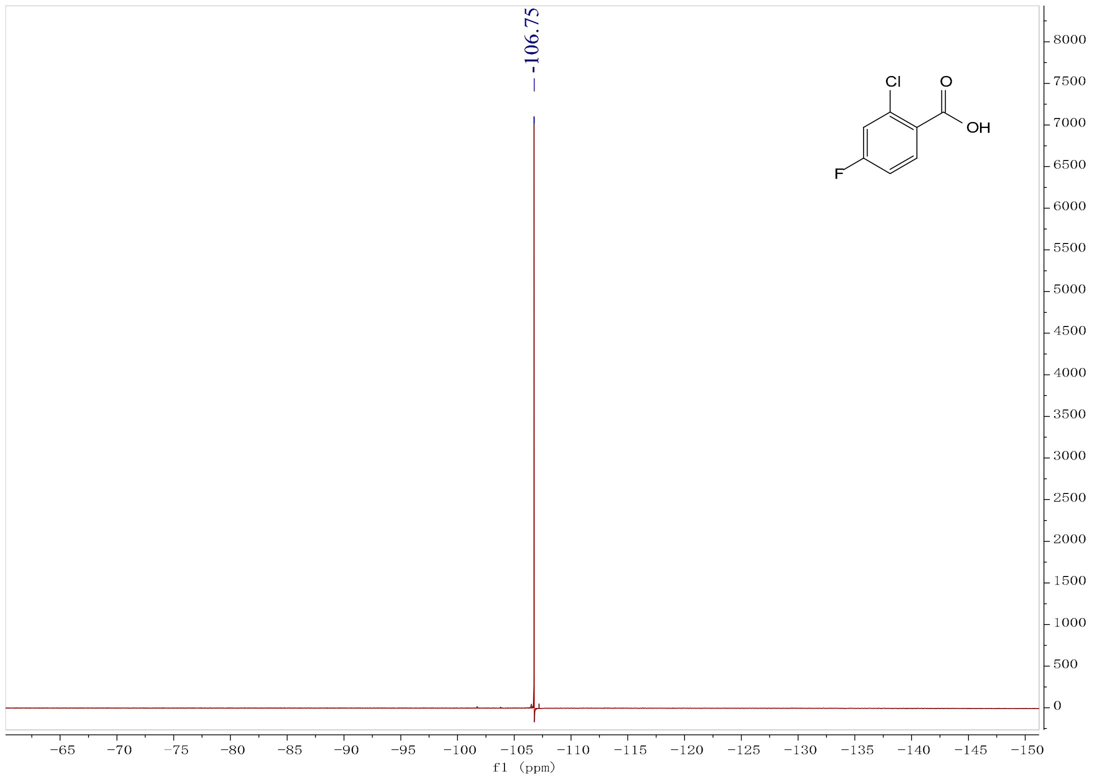

Figure S19. ^{1}**H (400 MHz),**^{13}**C {1H} (101 MHz) and** ^{19}**F {1H} (376 MHz) NMR spectra of 15c in DMSO–**d_6**.**

4–Cyanobenzoic acid (16c)

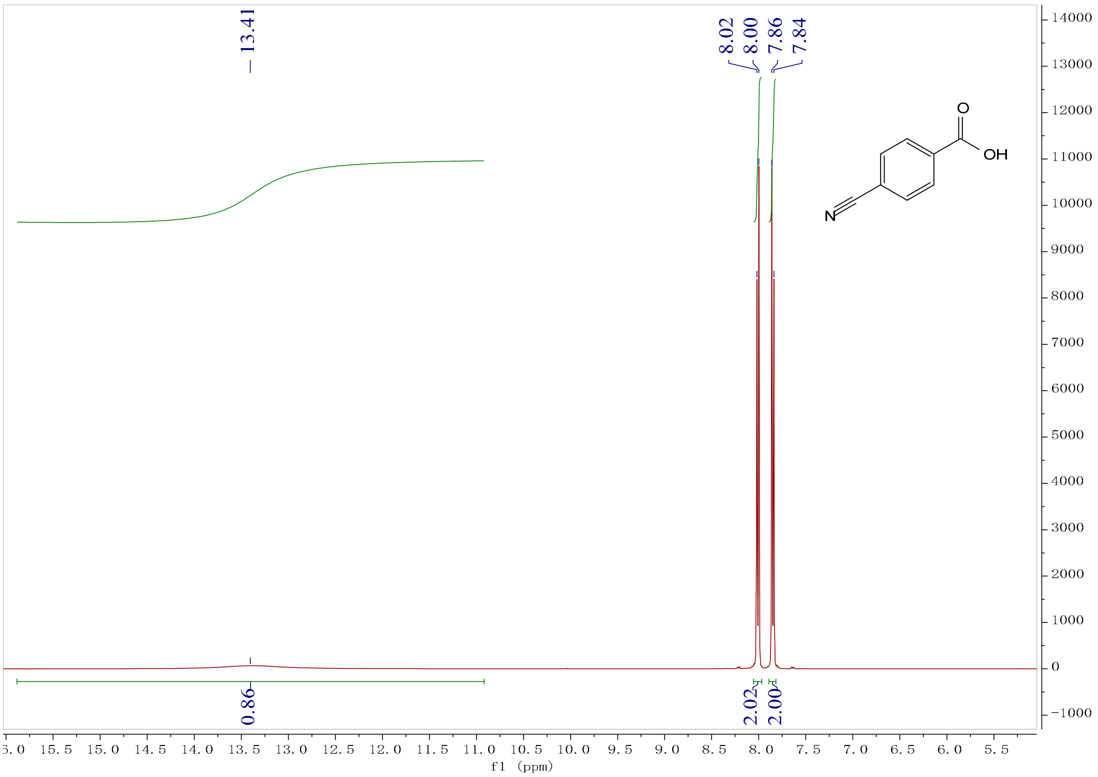

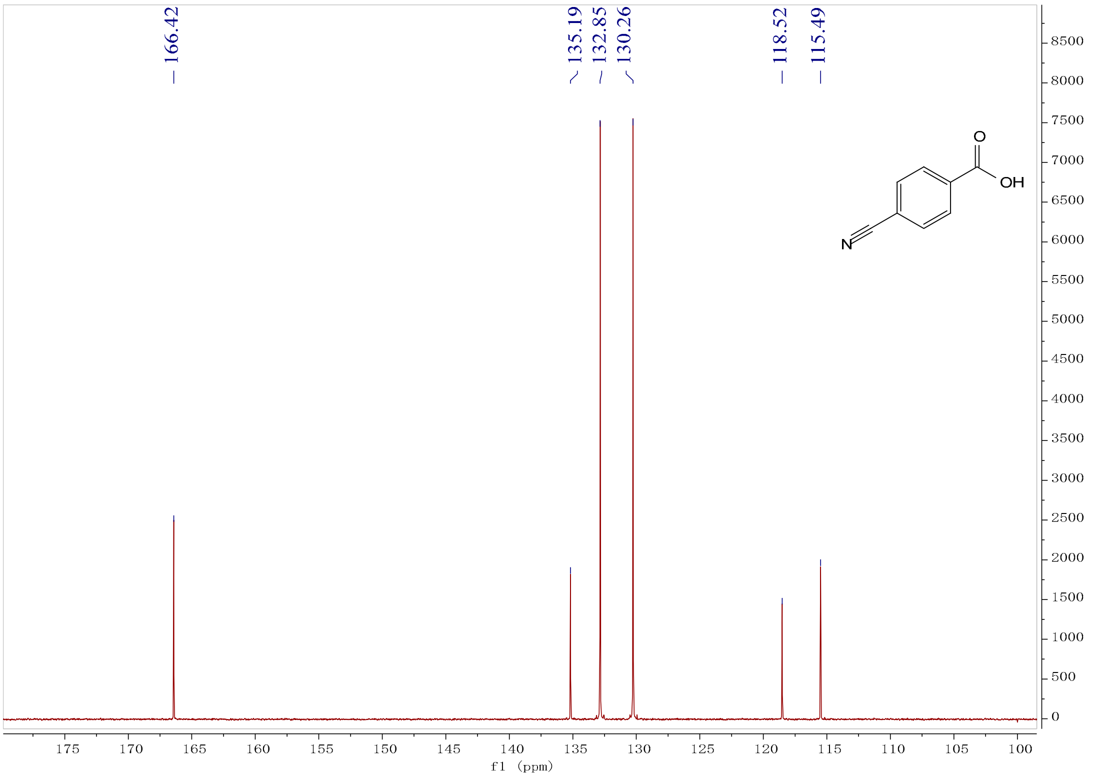

Figure S20. ^{1}**H (400 MHz) and** ^{13}**C {1H} (101 MHz) NMR spectra of 16c in DMSO–*d*$_6$.**

4–*Acetylbenzoic acid (17c)*

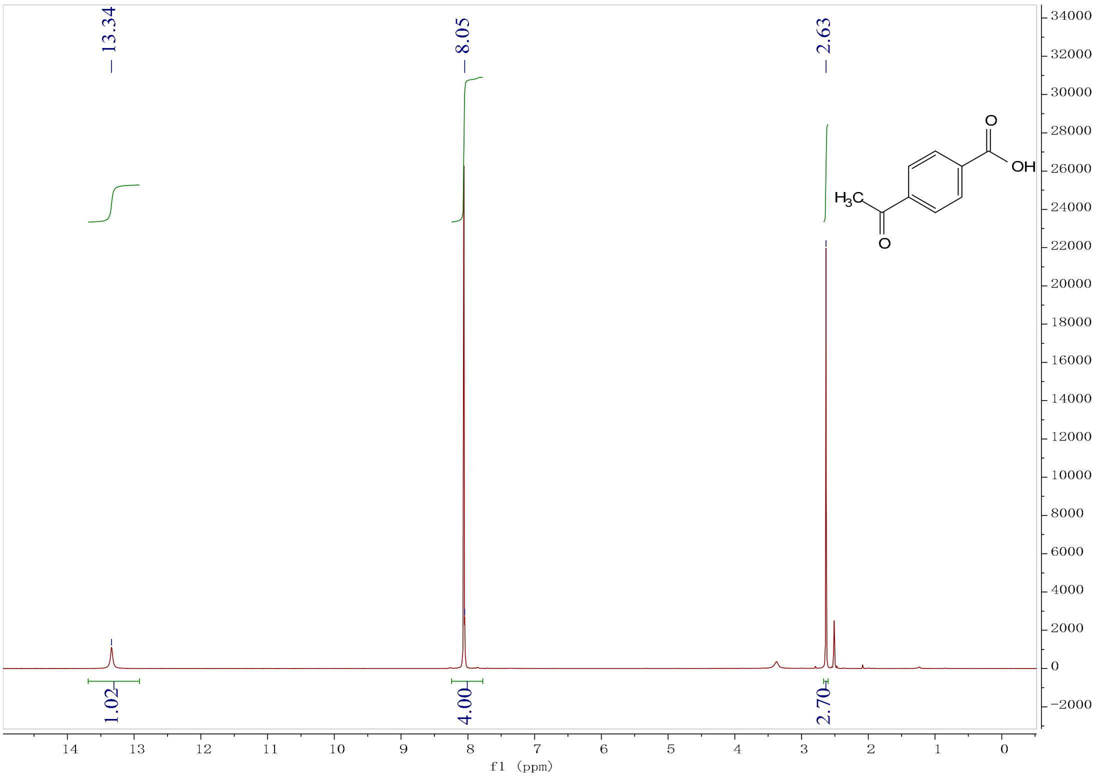

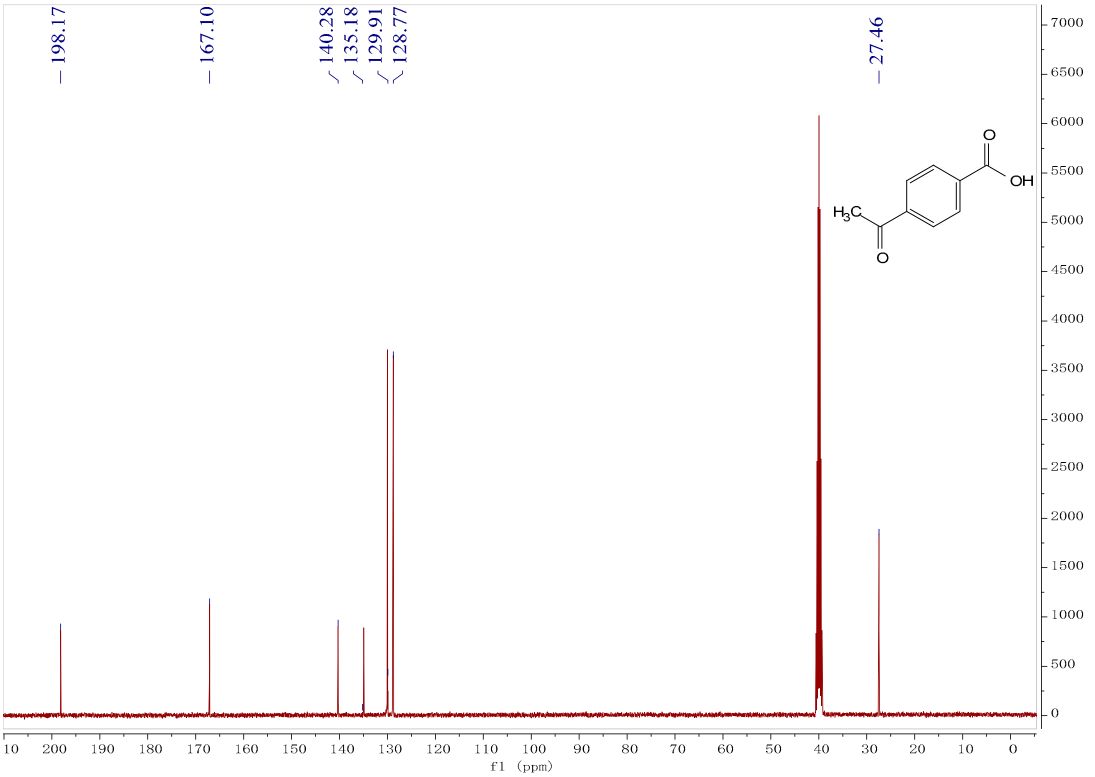

Figure S21. **^{1}H (400 MHz) and ^{13}C {1H} (101 MHz) NMR spectra of 17c in DMSO–*d_6*.**

4–(Trifluoromethyl)benzoic acid (18c)

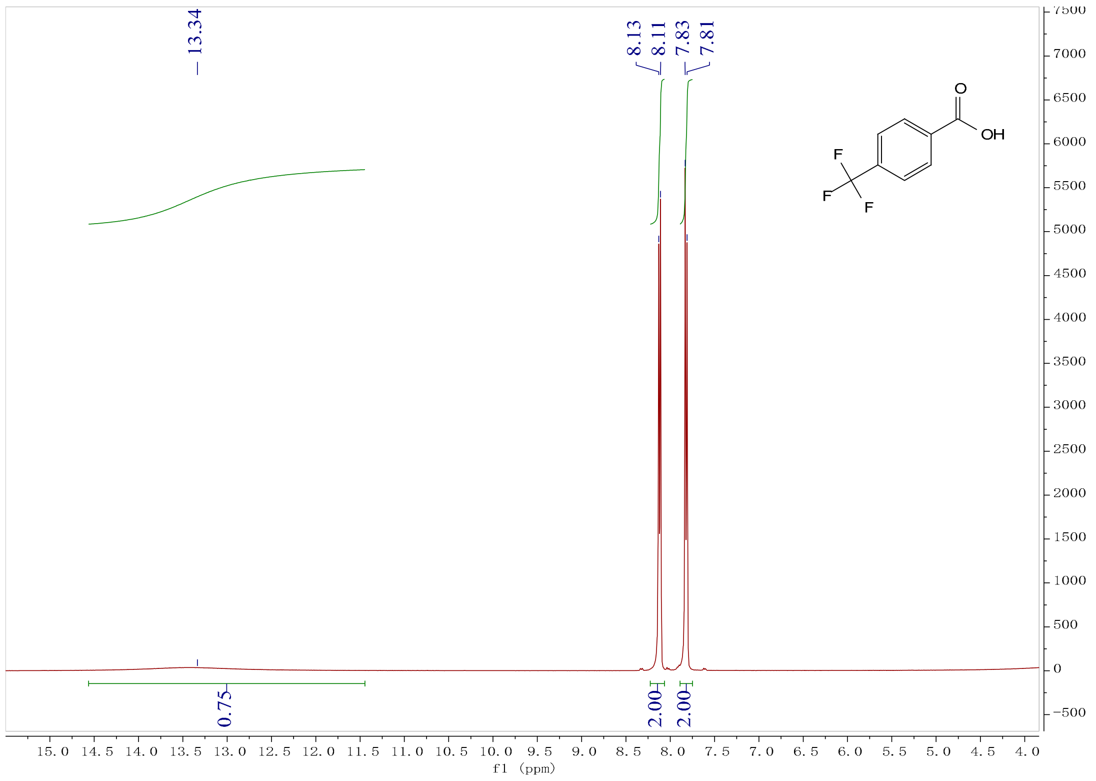

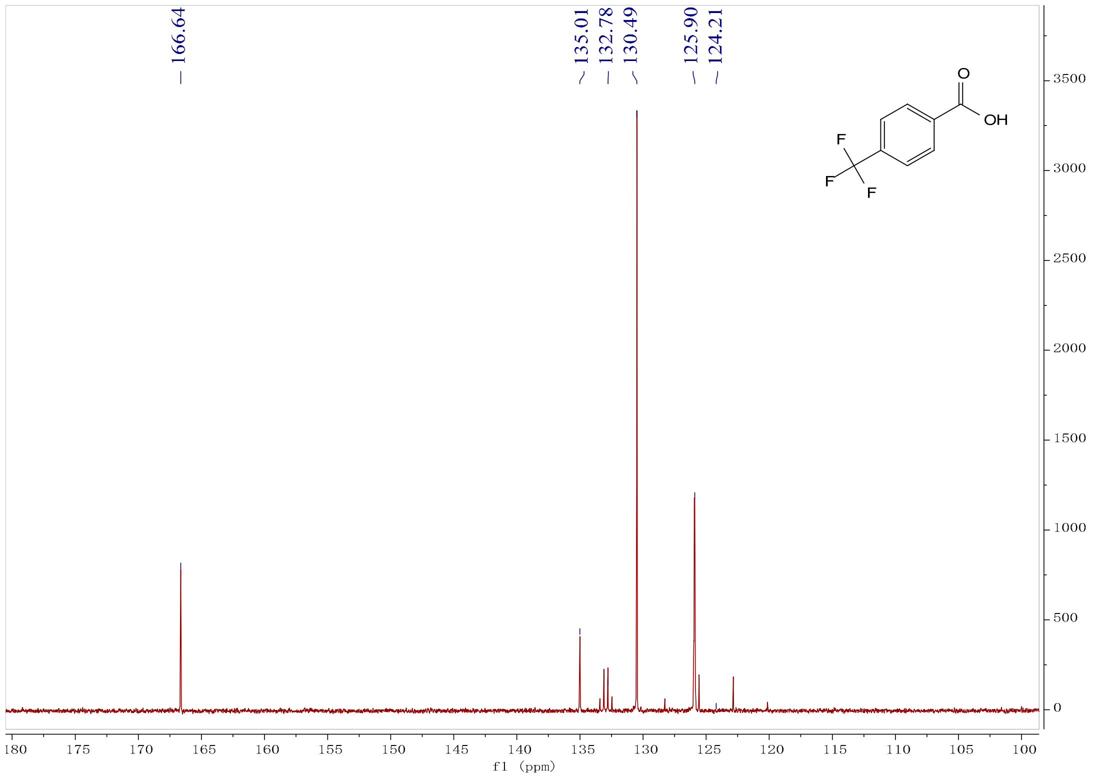

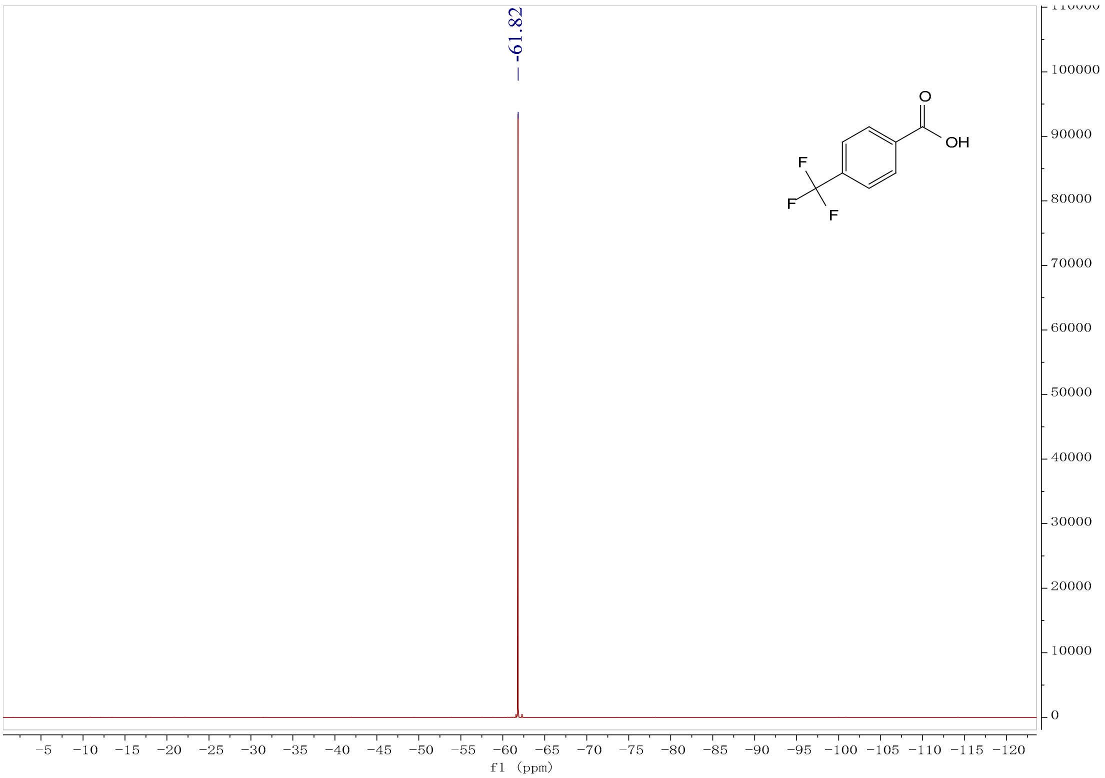

Figure S22. **^{1}H (400 MHz),^{13}C {1H} (101 MHz) and ^{19}F {1H} (376 MHz) NMR spectra of 18c in DMSO–d_6.**

4–(Trifluoromethoxy)benzoic acid (19c)

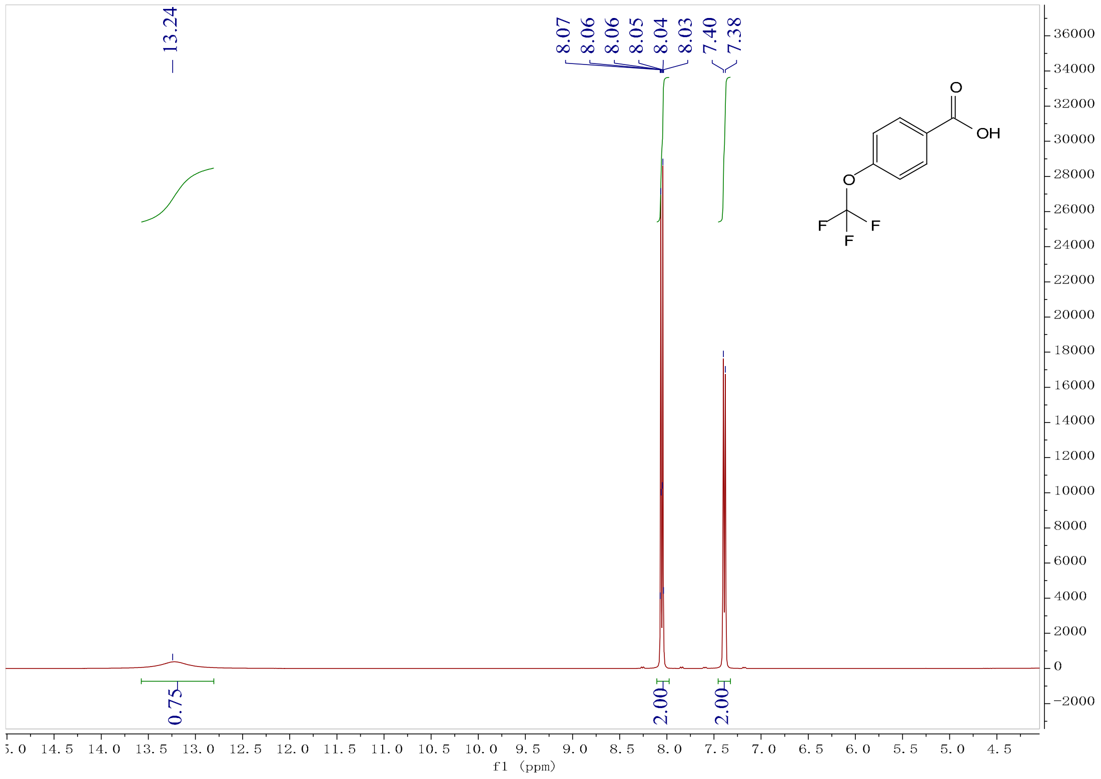

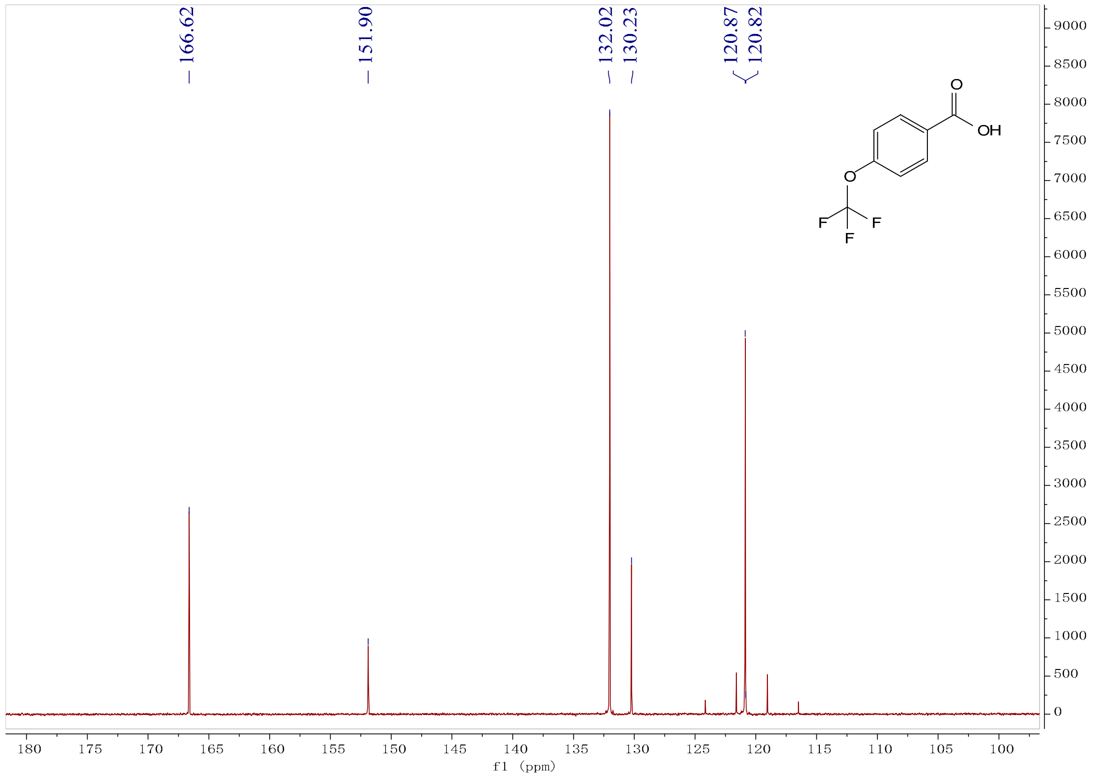

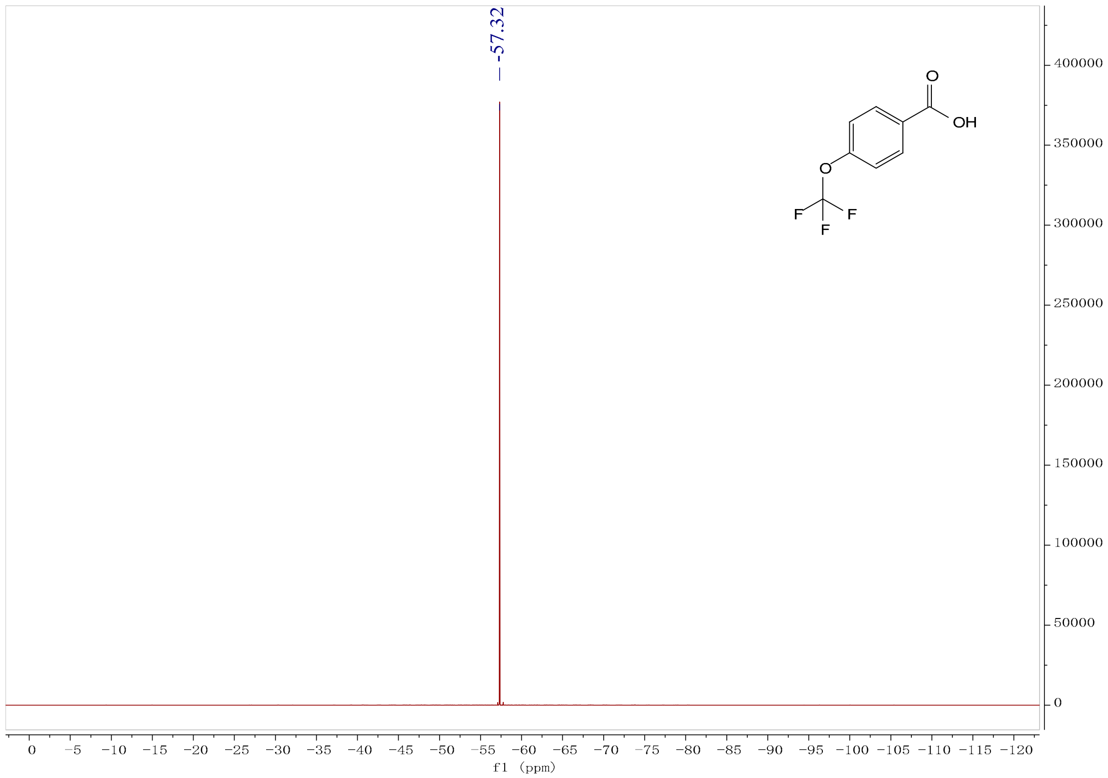

Figure S23. **^{1}H (400 MHz),^{13}C {1H} (101 MHz) and ^{19}F {1H} (376 MHz) NMR spectra of 19c in DMSO–d_6.**

4–Nitrobenzoic acid (20c)

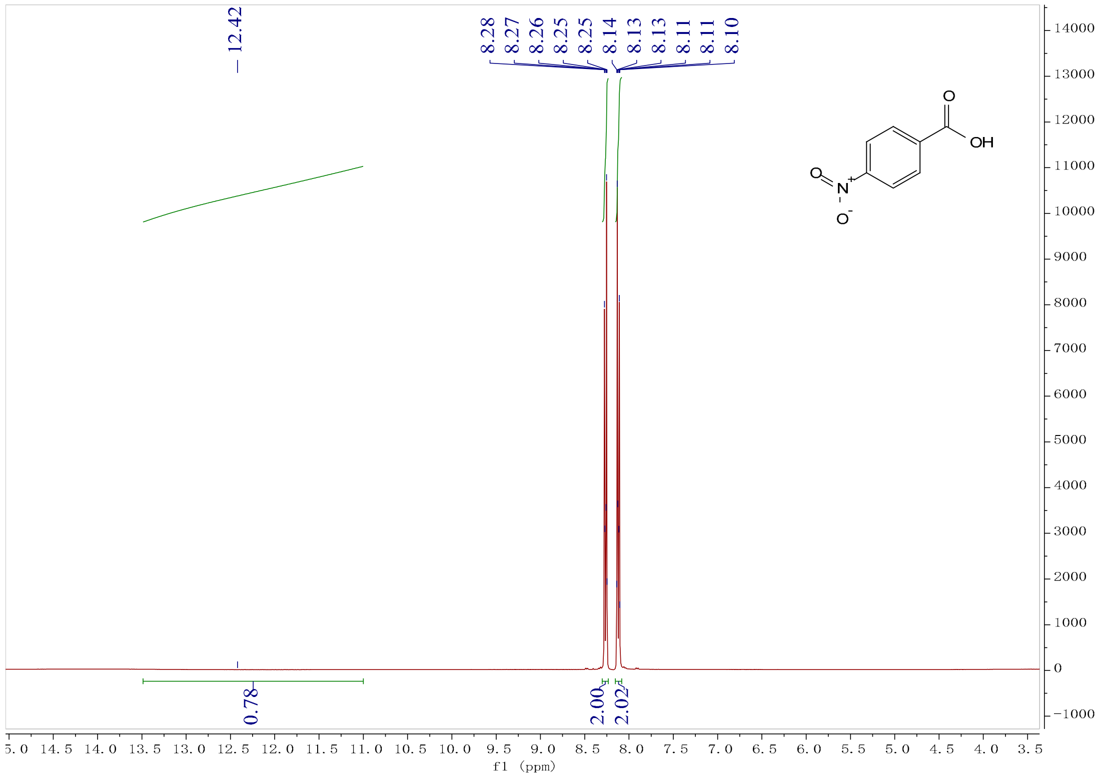

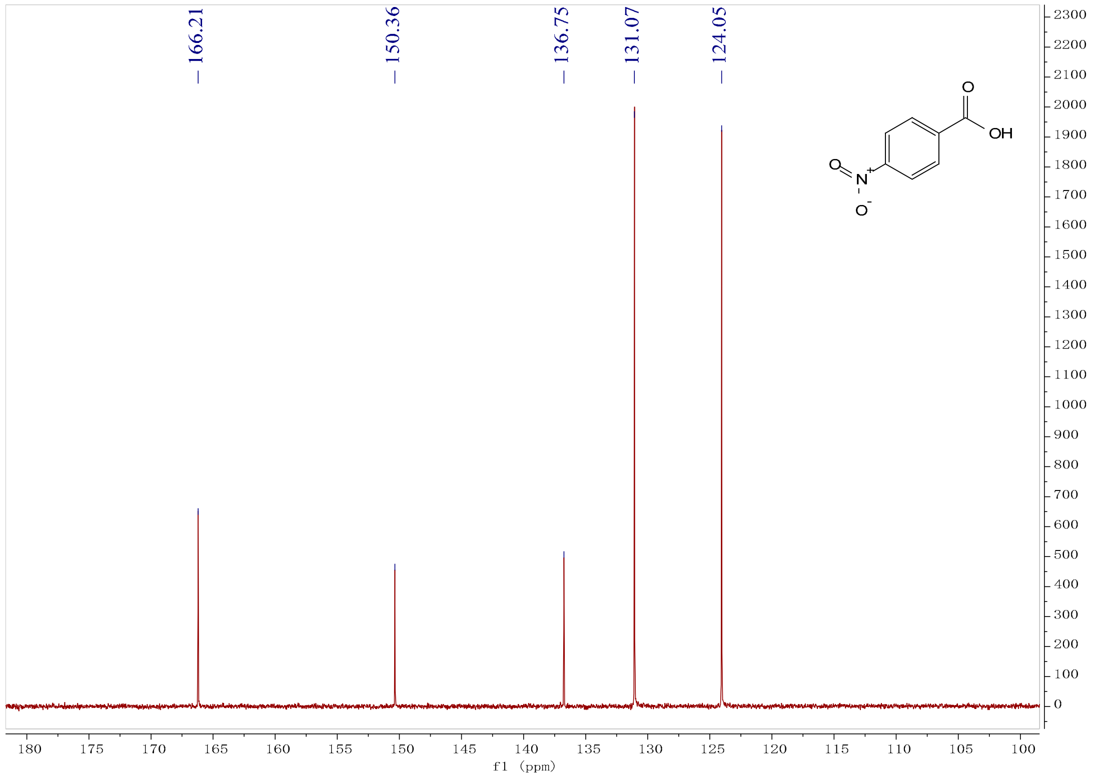

Figure S24. **^{1}H (400 MHz) and ^{13}C {1H} (101 MHz) NMR spectra of 20c in DMSO–*d*$_6$.**

Terephthalic acid (21c)

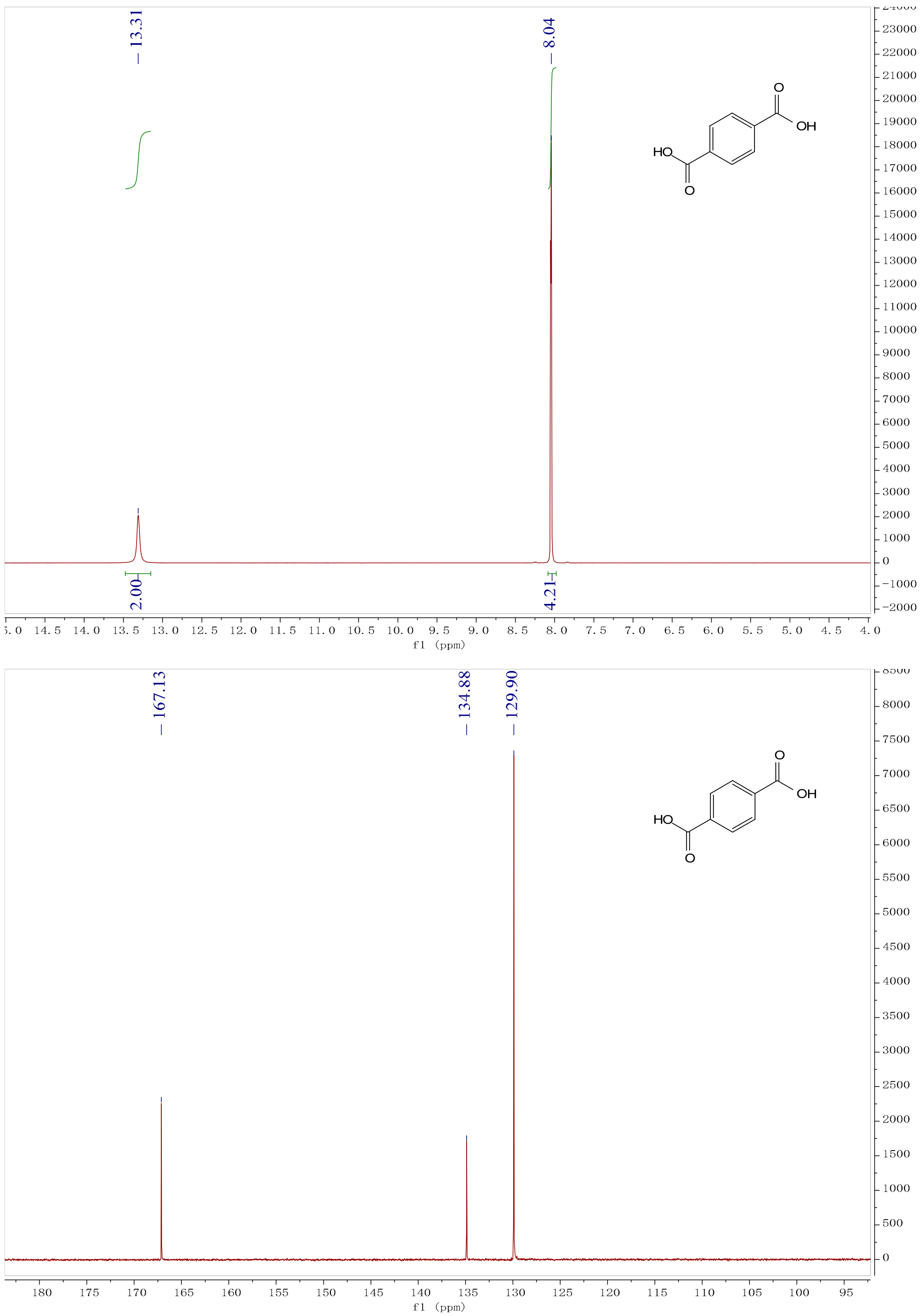

Figure S25. ^{1}H (400 MHz) and ^{13}C {1H} (101 MHz) NMR spectra of 21c in DMSO–d_6.

2–Thiophenic acid (22c)

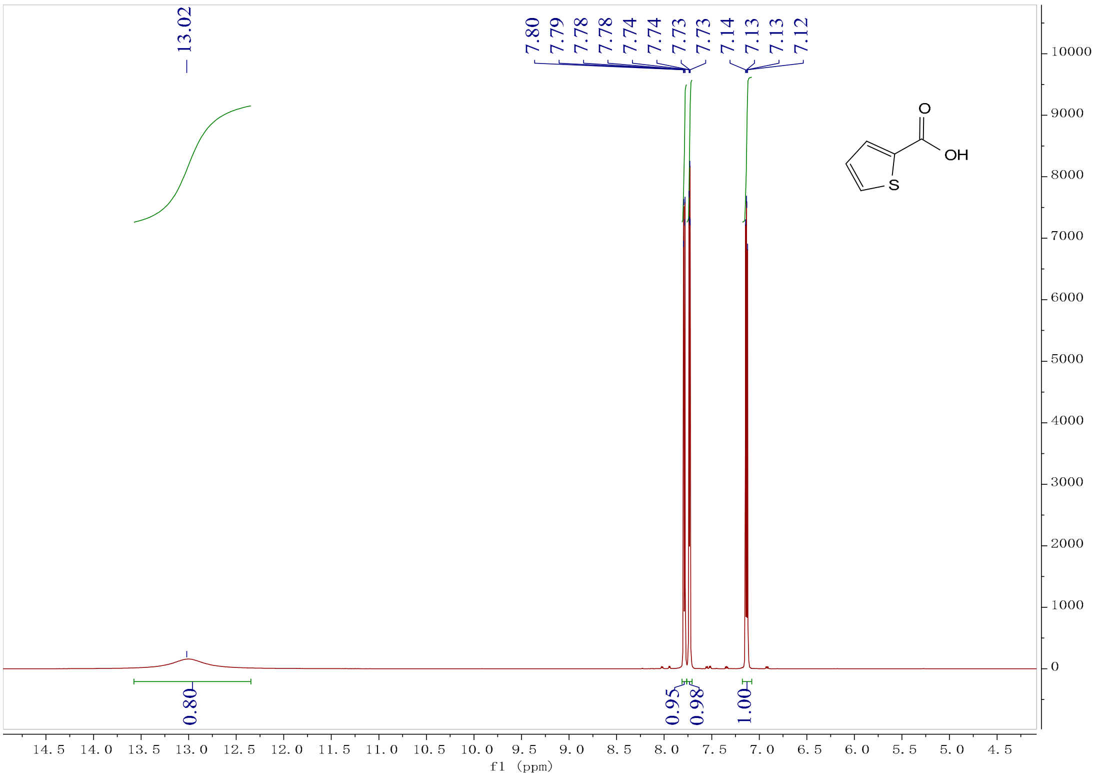

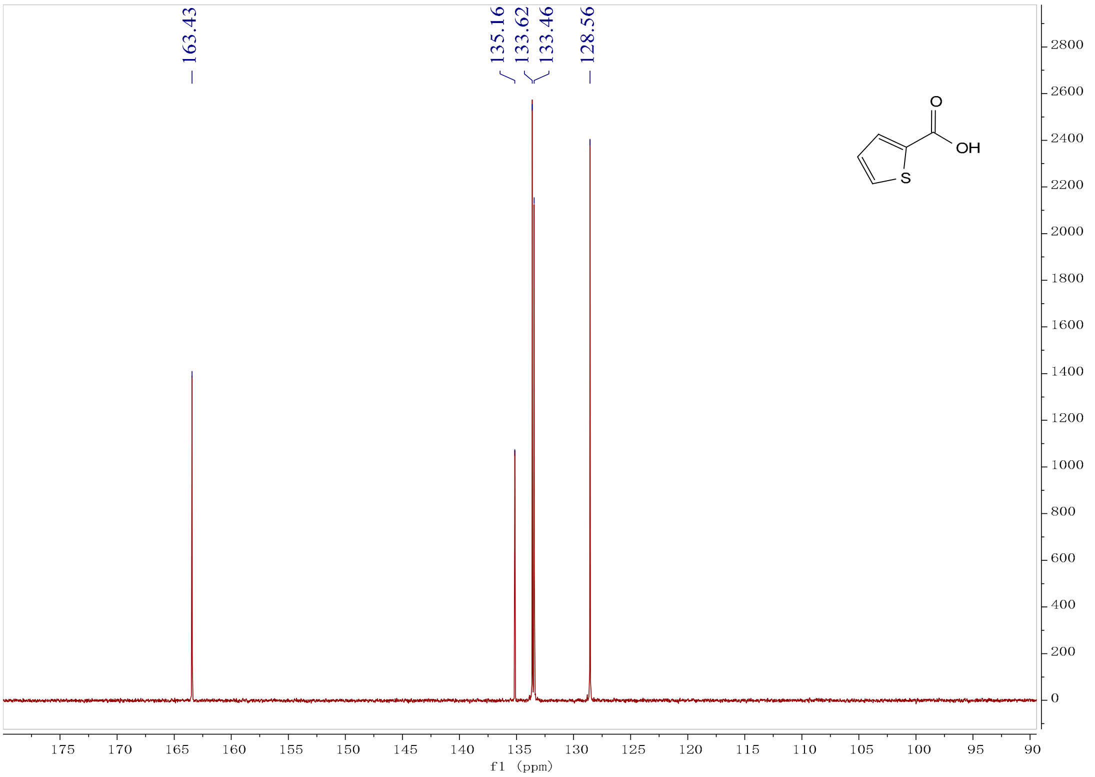

Figure S26. ^{1}H (400 MHz) and ^{13}C {1H} (101 MHz) NMR Spectra of 22c in DMSO–d_6.

4–Bromo–2–thiophenecarboxylic acid (23c)

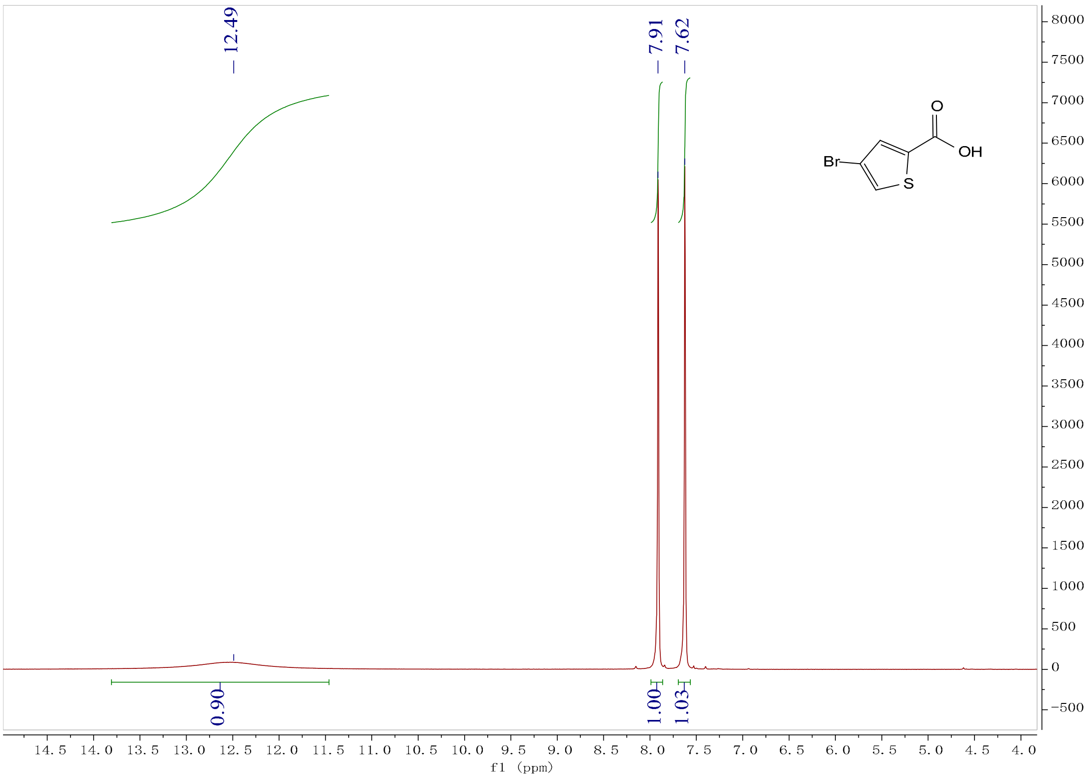

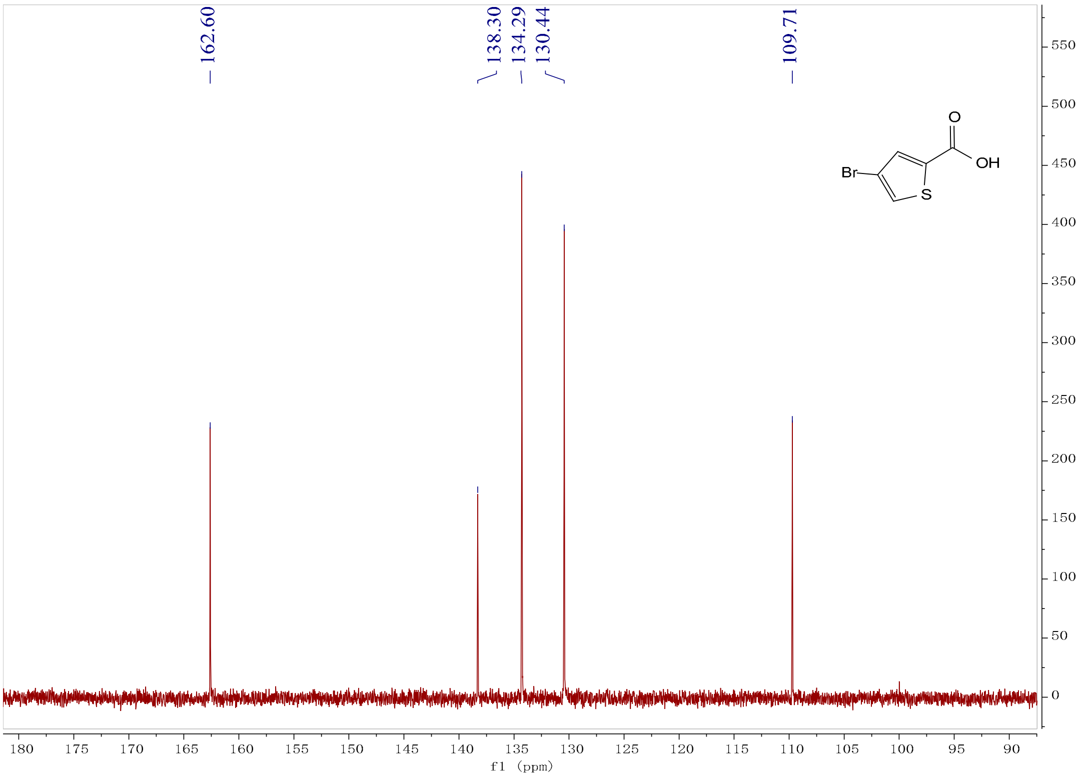

Figure S27. ^{1}H (400 MHz) and ^{13}C {1H} (101 MHz) NMR Spectra of 23c in DMSO–*d*$_6$.

1–Methyl–1H–pyrazole–5–carboxylic acid (24c)

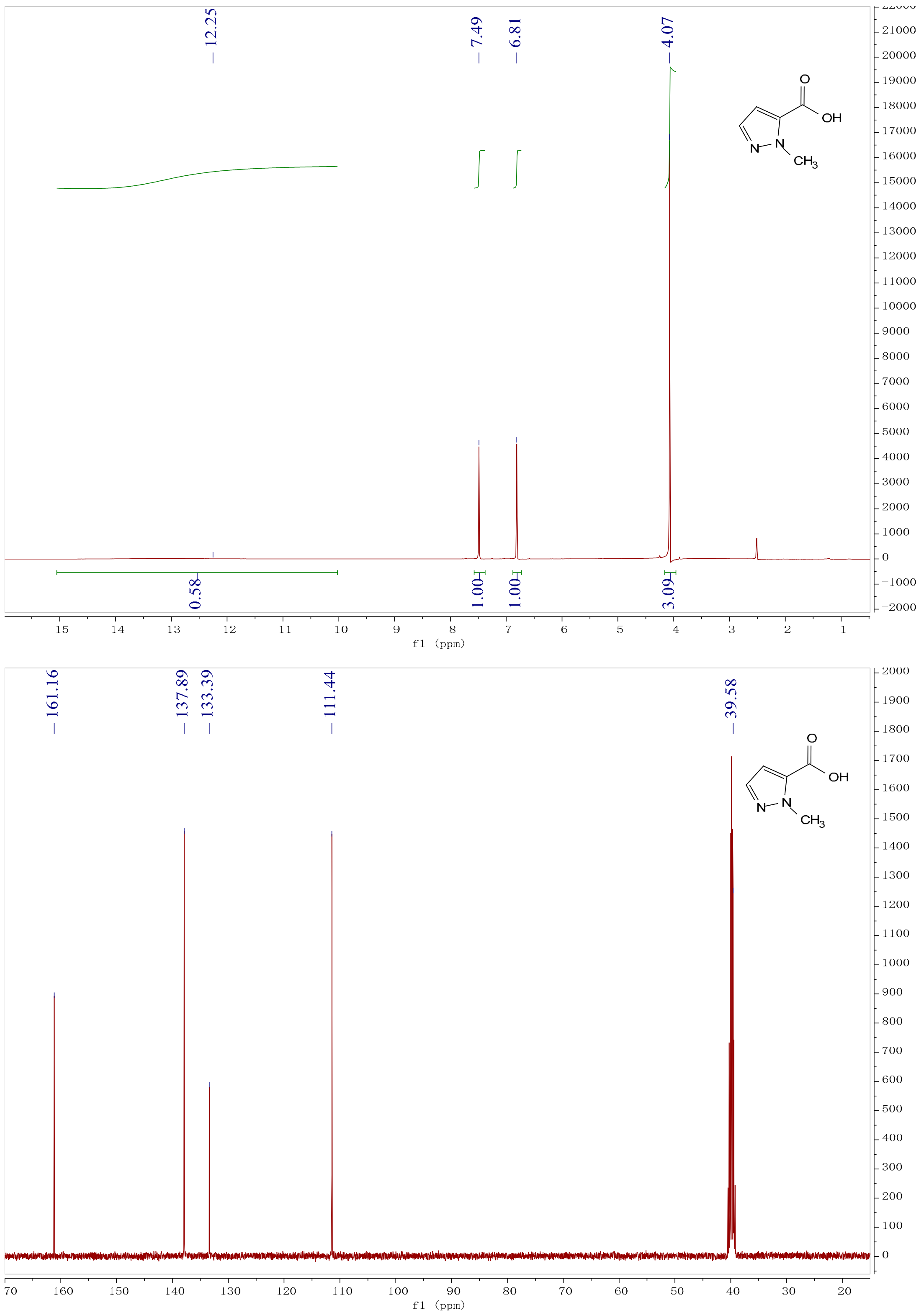

Figure S28. ^{1}H **(400 MHz) and** ^{13}C **{1H} (101 MHz) NMR spectra of 24c in DMSO–*d*$_6$.**

Nicotinic Acid (25c)

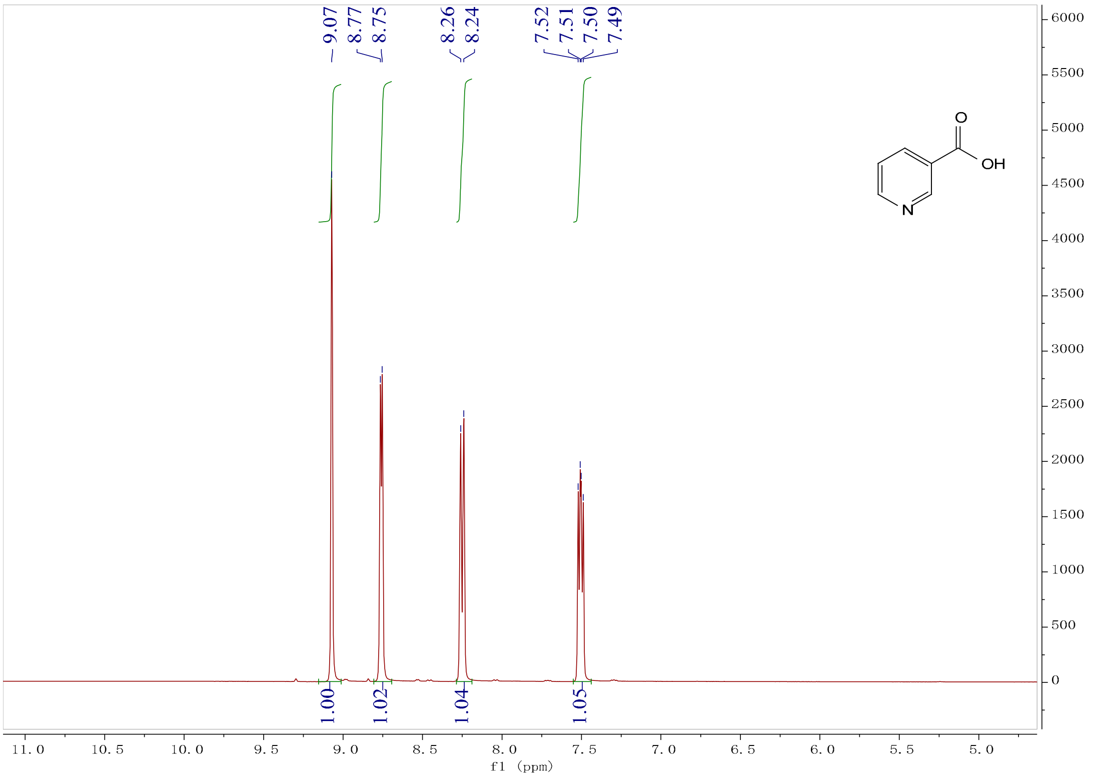

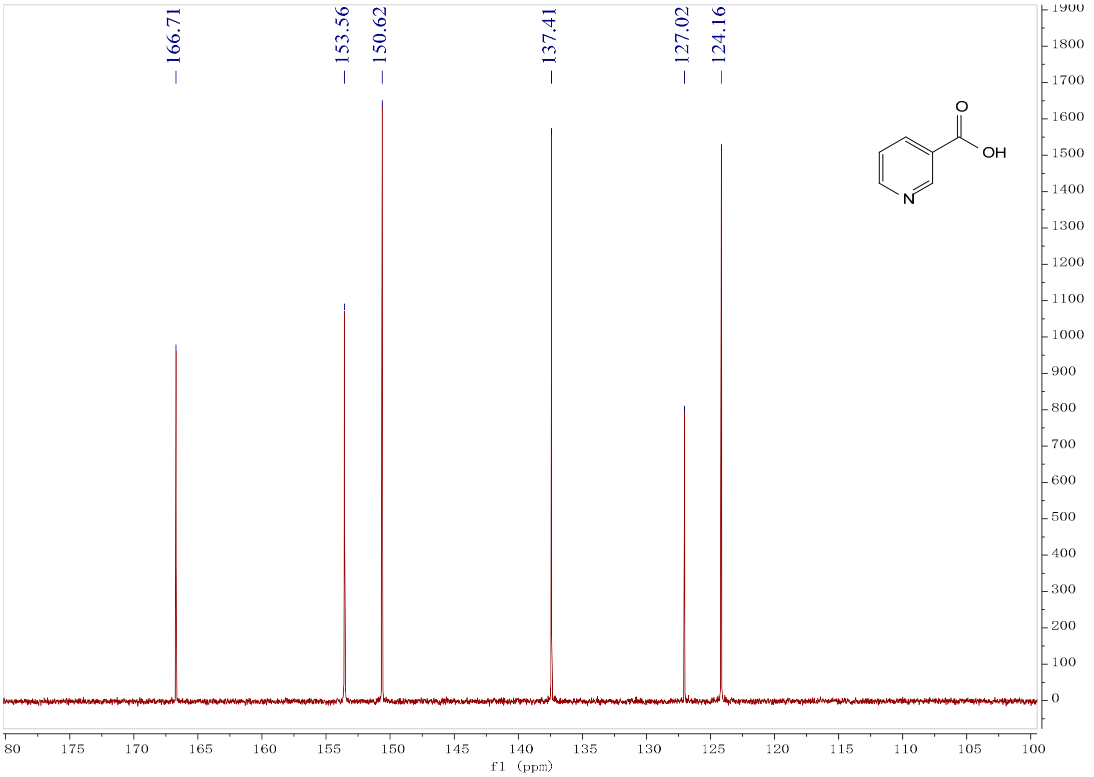

Figure S29. ^{1}H (400 MHz) and ^{13}C {1H} (101 MHz) NMR spectra of 25c in DMSO–*d*$_6$.

4–Pyridinecarboxylic acid (26c)

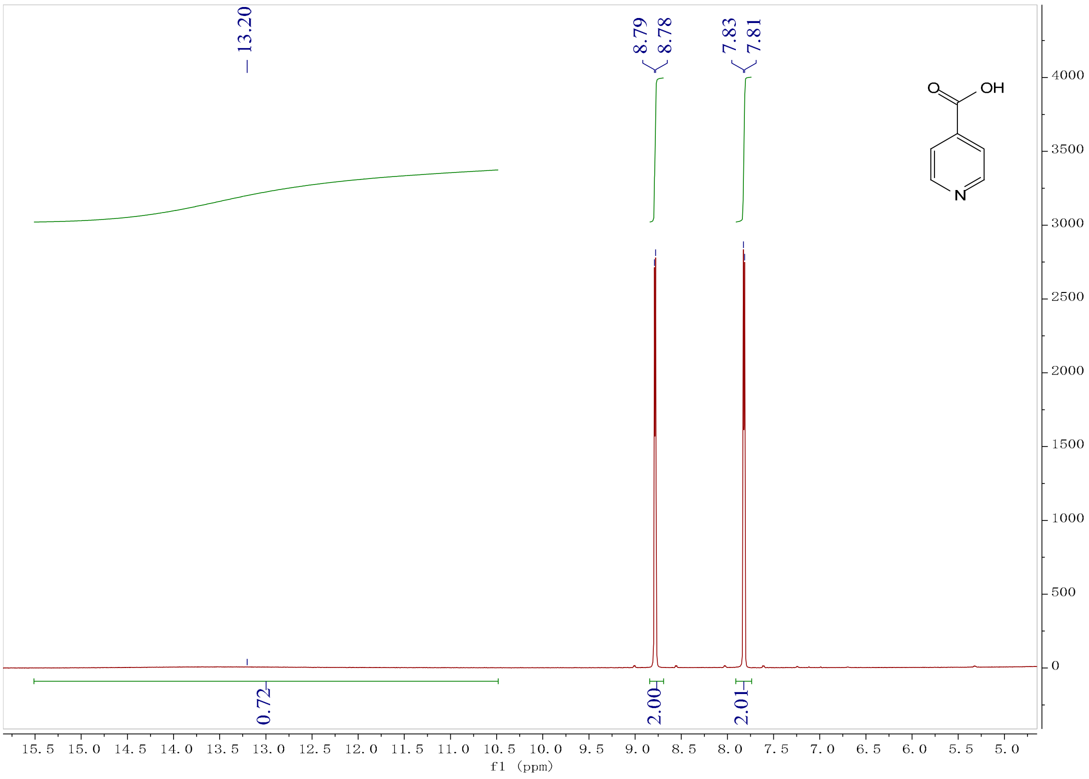

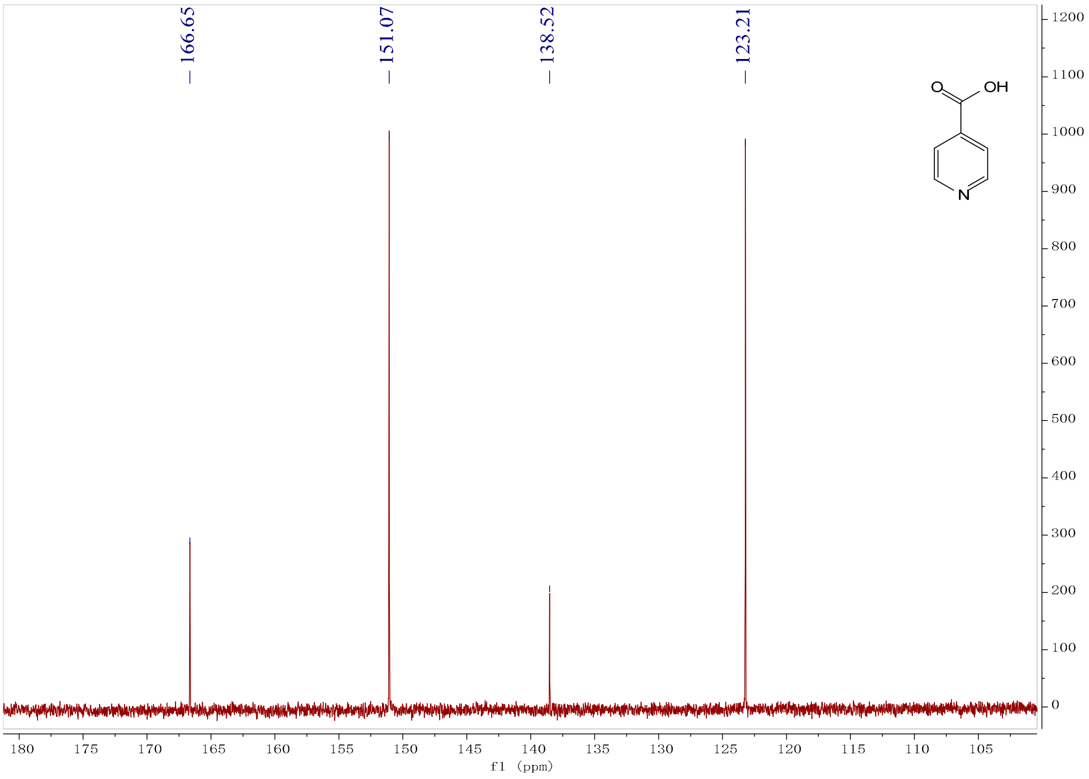

Figure S30. ^{1}H (400 MHz) and ^{13}C {1H} (101 MHz) NMR spectra of 26c in DMSO–d_6.

Quinoline–3–carboxylic acid (27c)

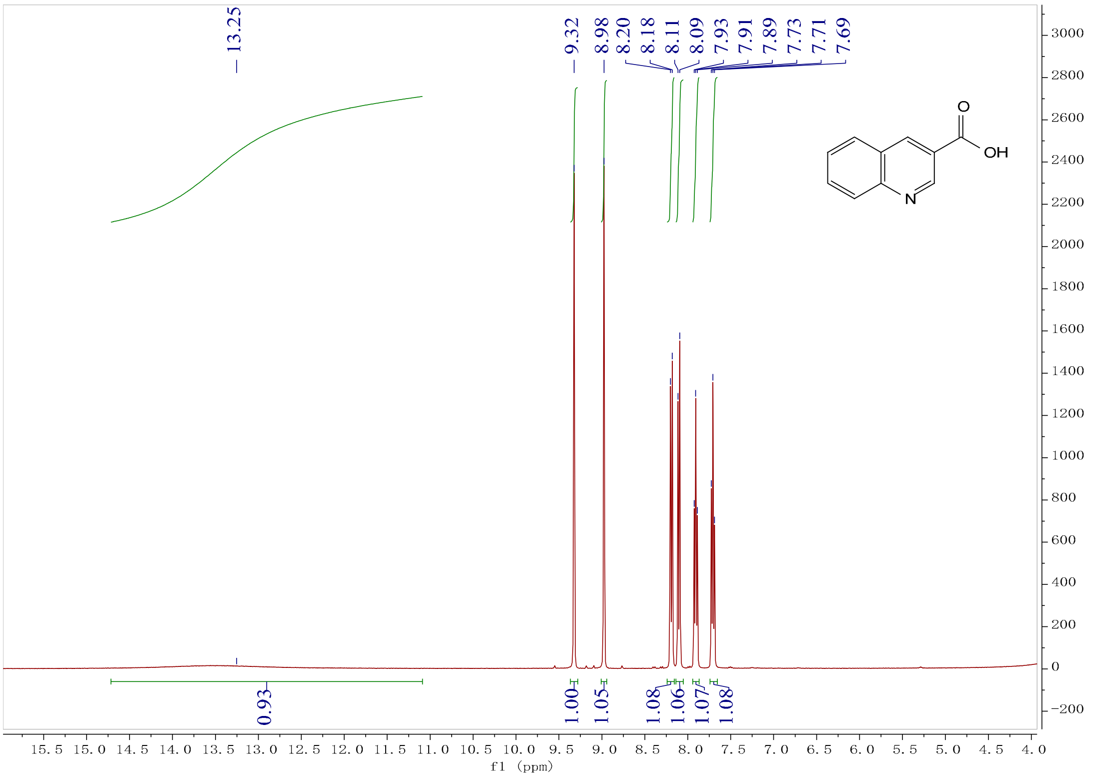

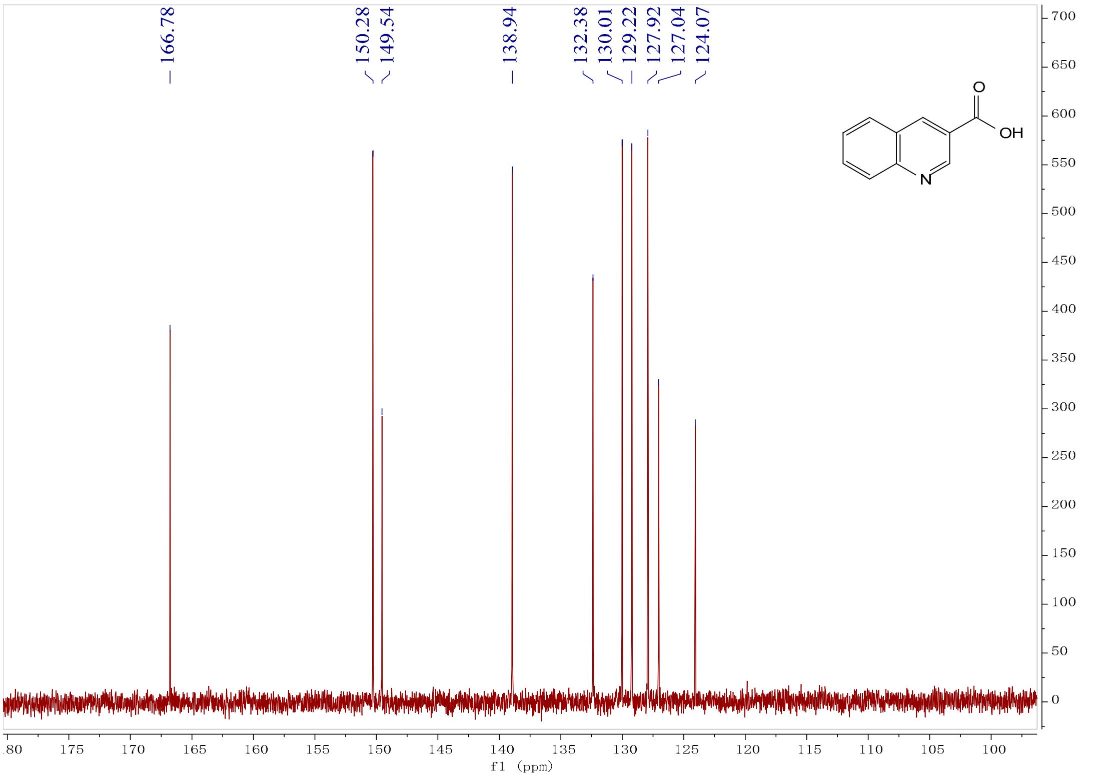

Figure S31. **^{1}H (400 MHz) and ^{13}C {1H} (101 MHz) NMR spectra of 27c in DMSO–*d_6*.**

2–Phenylquinoline–4–carboxylic acid (28c)

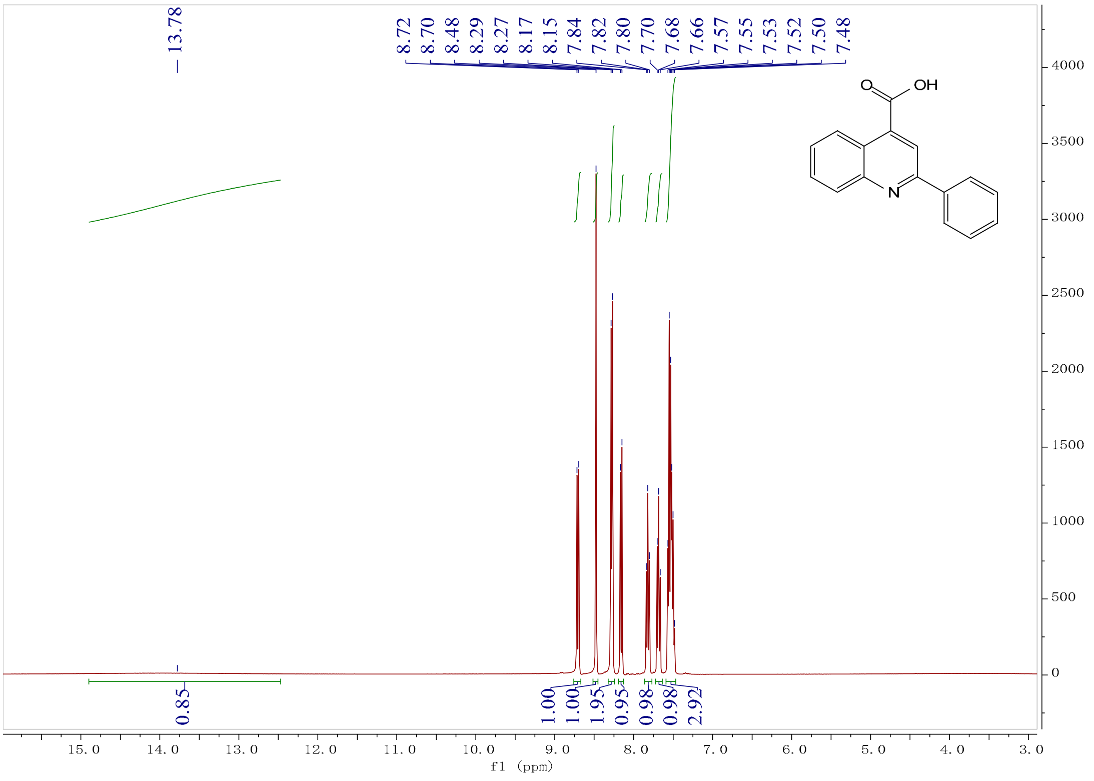

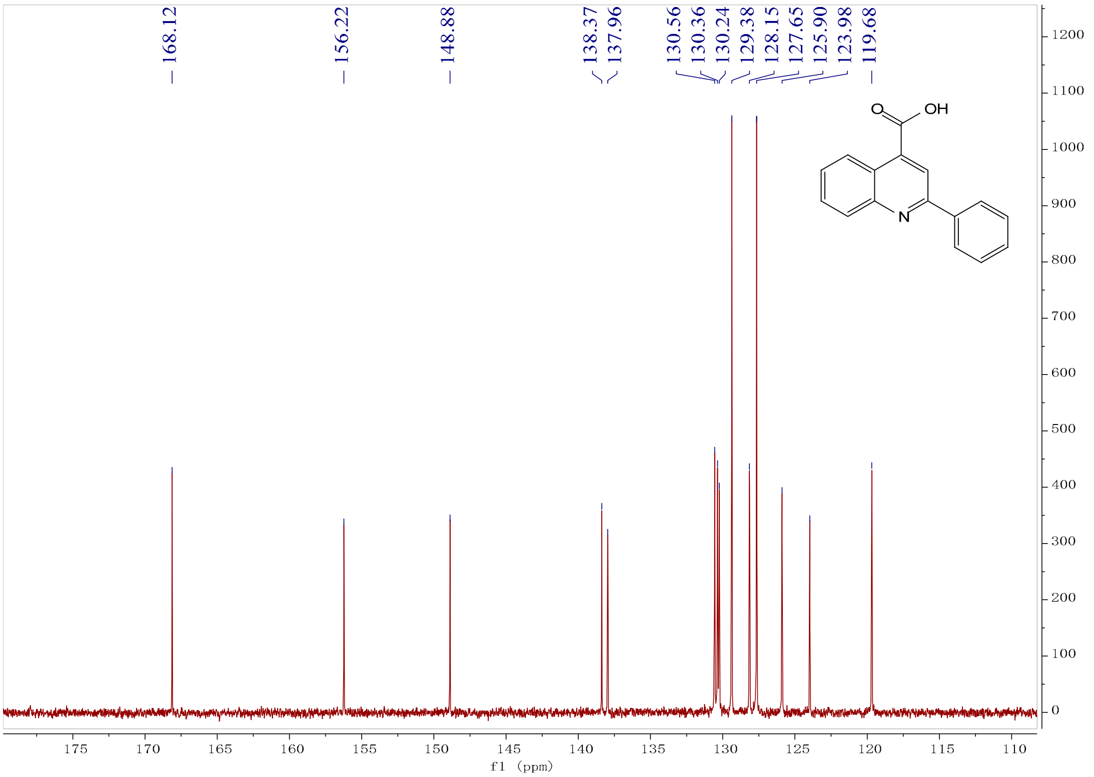

Figure S32. ^{1}H (400 MHz) and ^{13}C {1H} (101 MHz) NMR spectra of 28c in DMSO–*d*$_6$.

Acetophenone (29c)

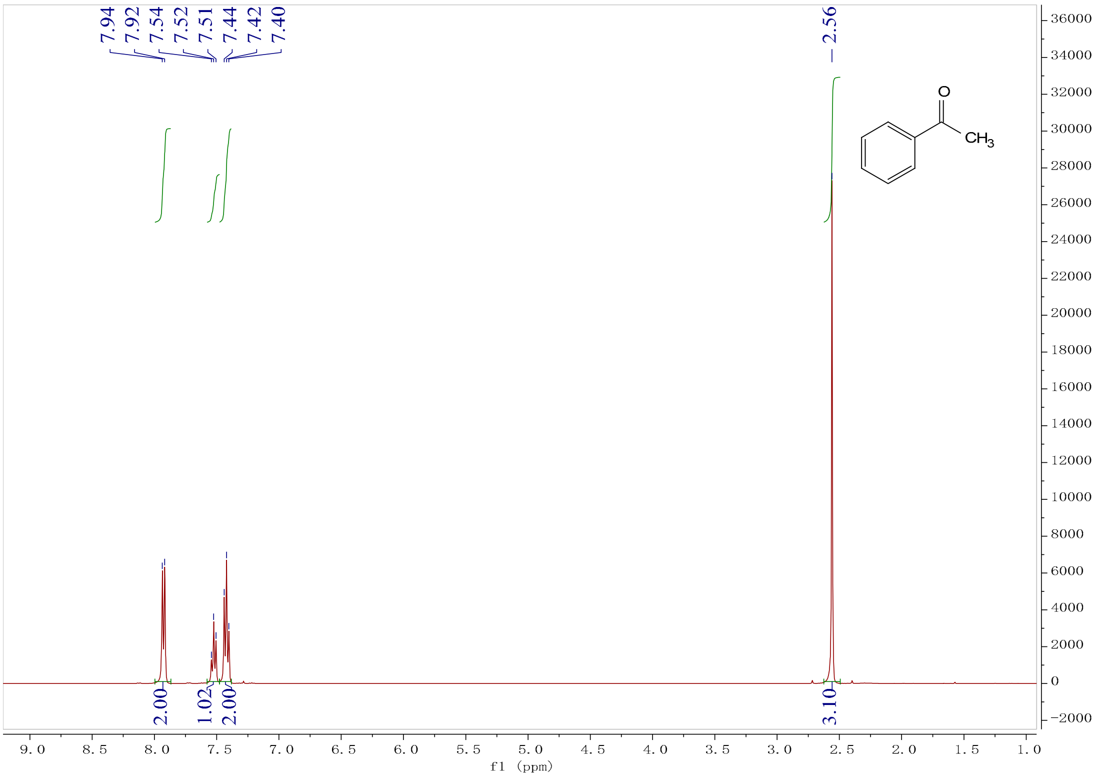

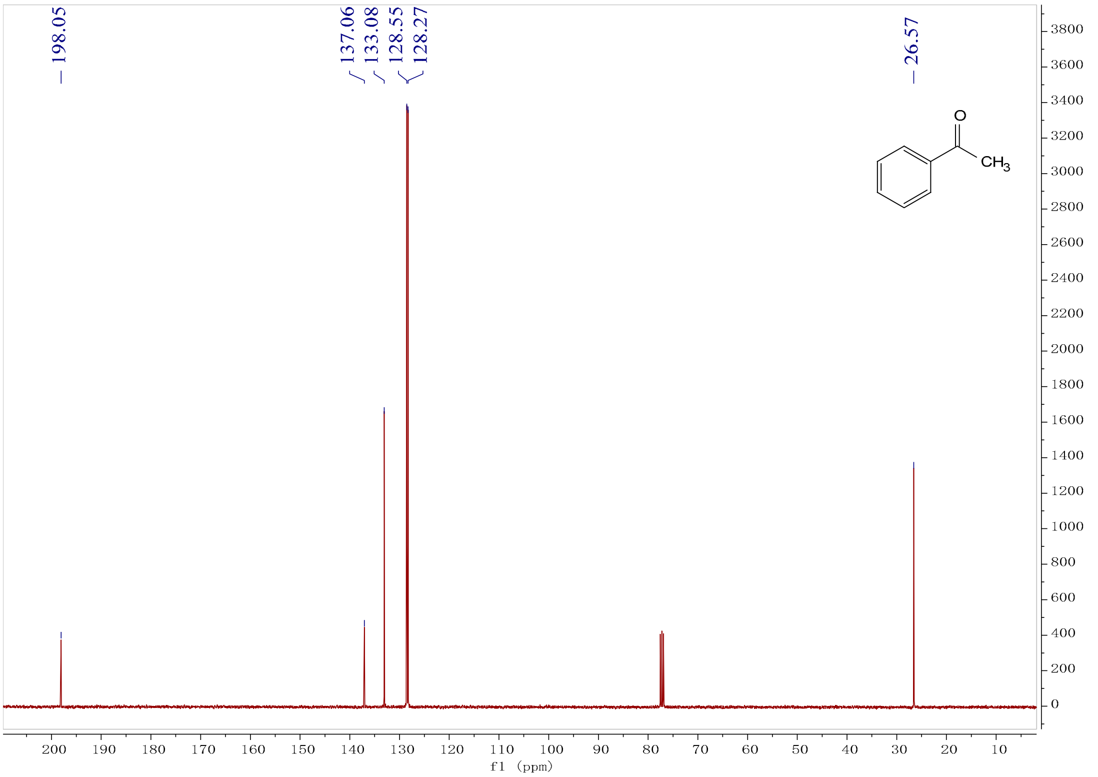

Figure S33. ^{1}H (400 MHz) and ^{13}C {1H} (101 MHz) NMR spectra of 29c in $CDCl_3$.

4′–Methylacetophenone (30c)

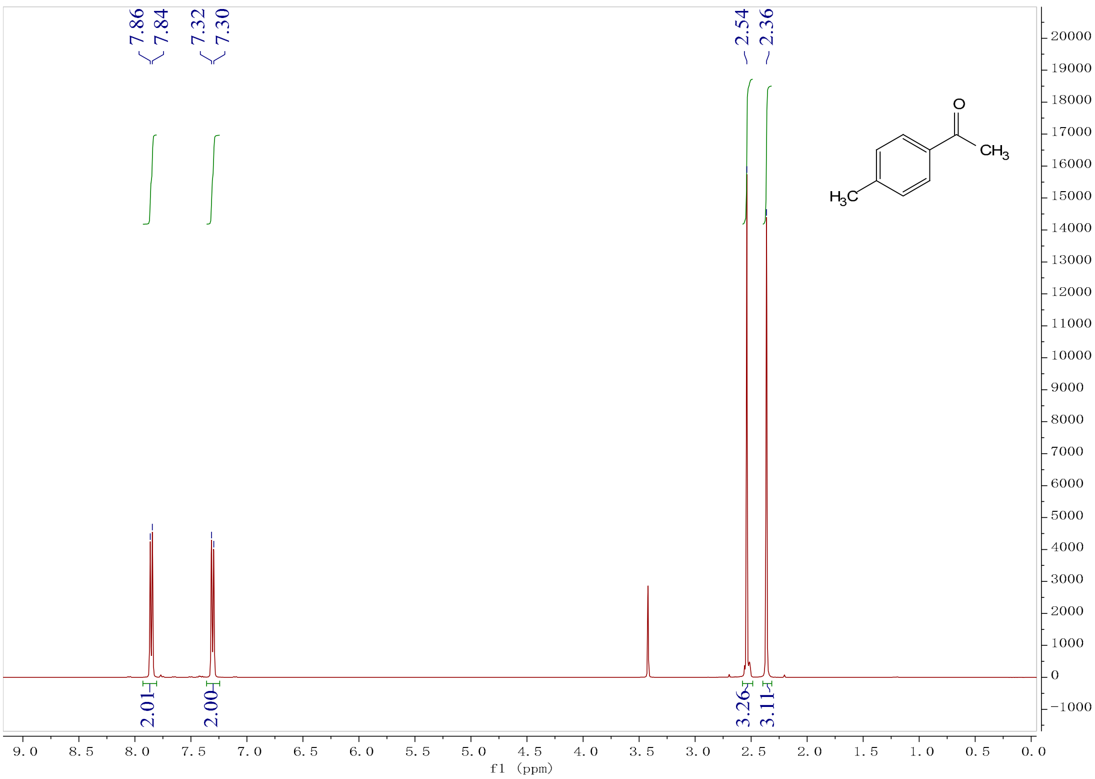

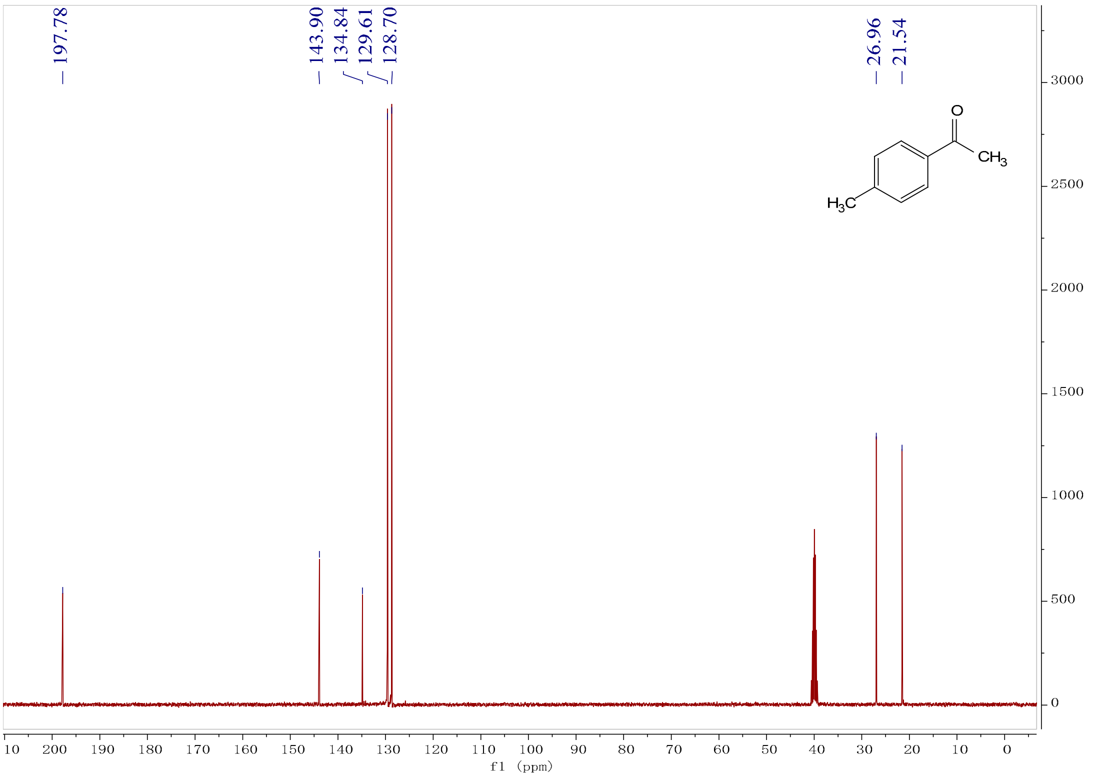

Figure S34. ^{1}H (400 MHz) and ^{13}C {1H} (101 MHz) NMR spectra of 30c in DMSO–d_6.

4′–Methoxyacetophenone (31c)

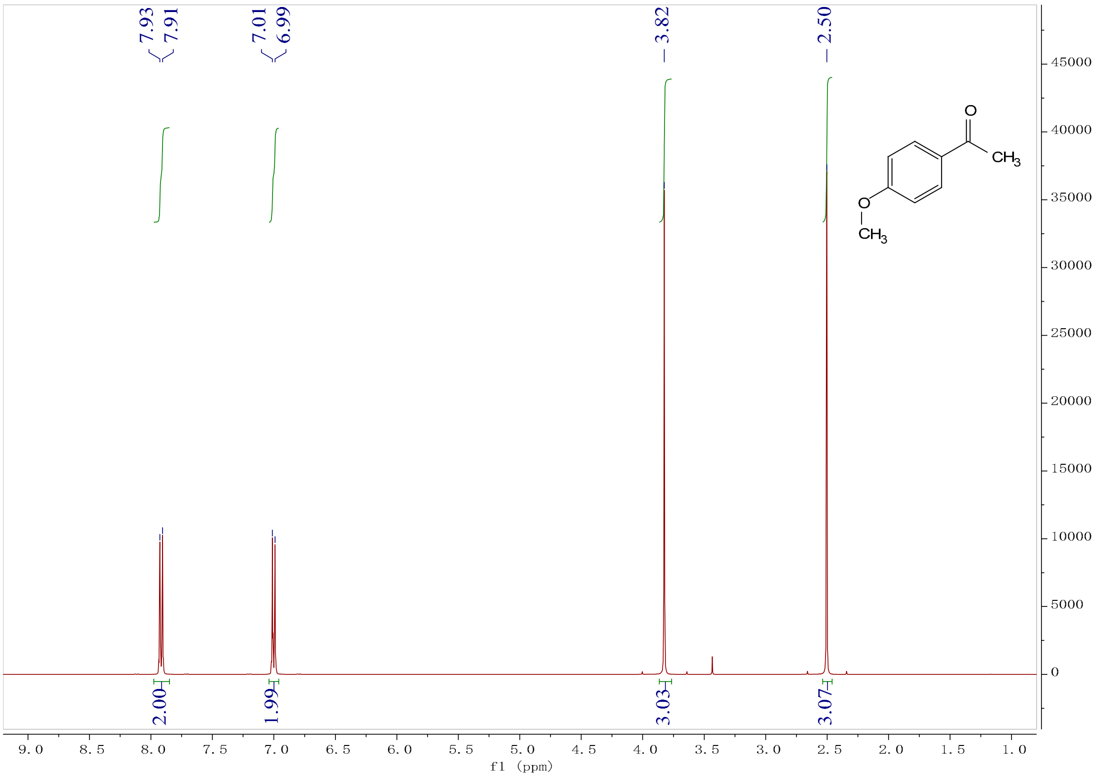

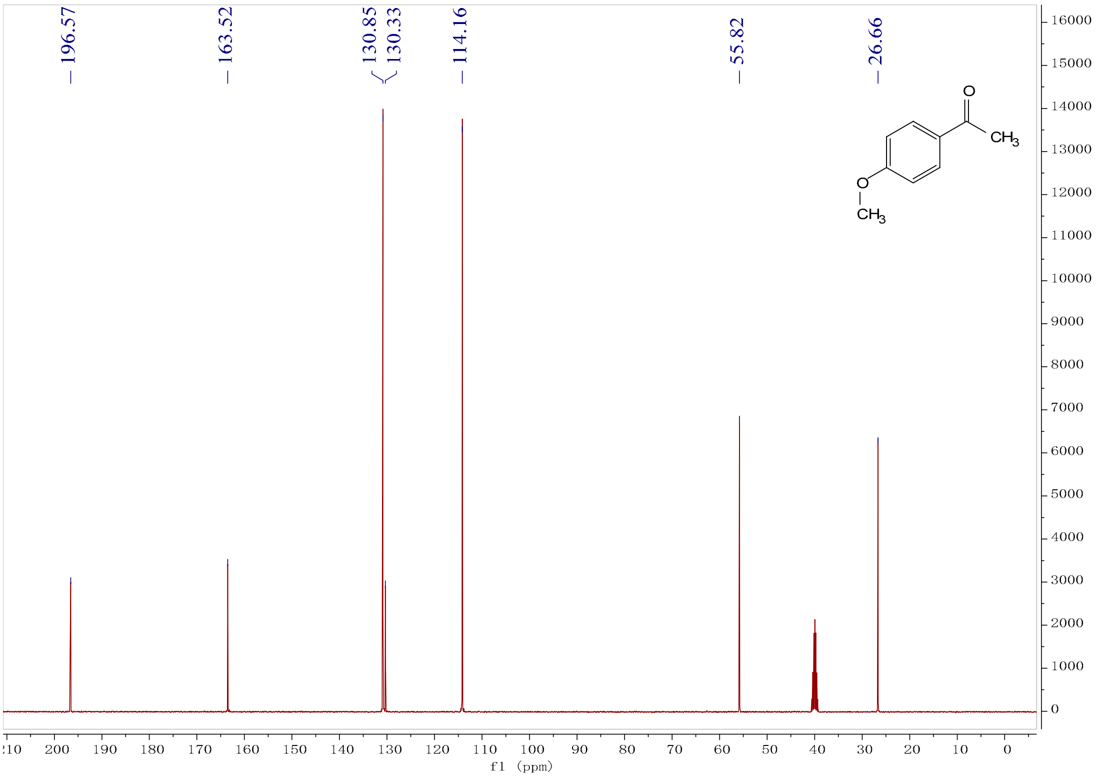

Figure S35. ^{1}H (400 MHz) and ^{13}C {1H} (101 MHz) NMR spectra of 31c in DMSO–d_6.

4′–Chloroacetophenone (32c)

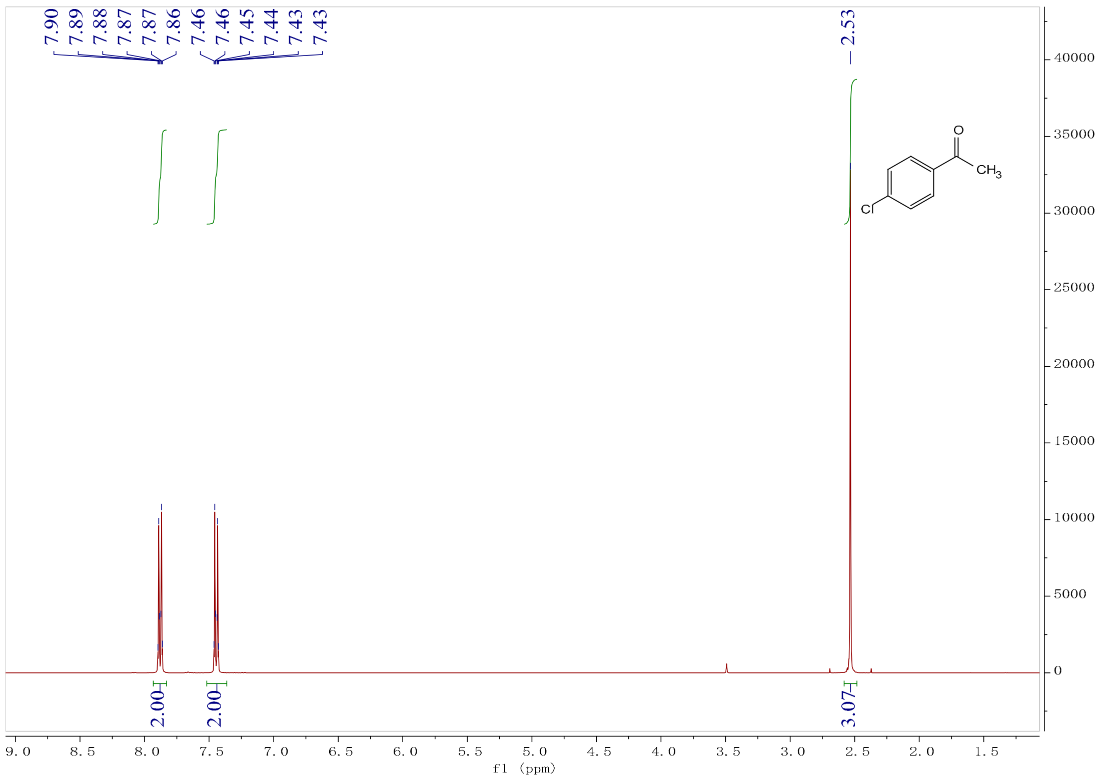

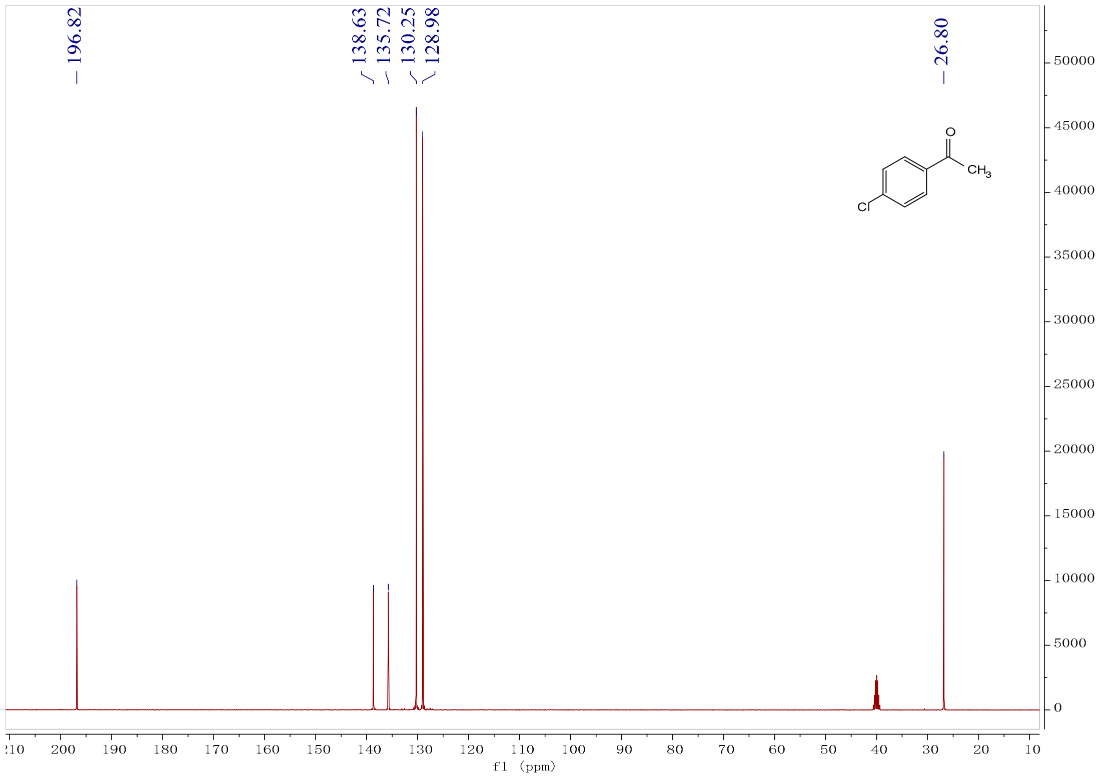

Figure S36. ^{1}**H (400 MHz) and** ^{13}**C {1H} (101 MHz) NMR spectra of 32c in DMSO–*d*$_6$.**

4′–Trifluoromethylacetophenone (33c)

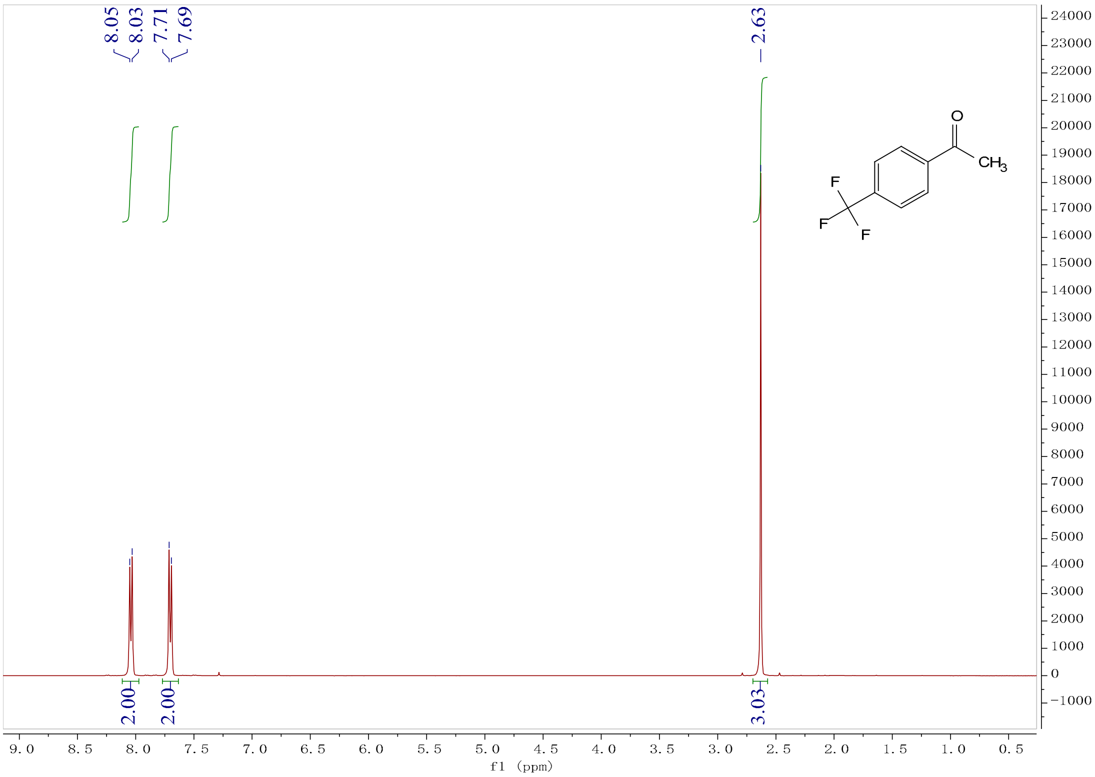

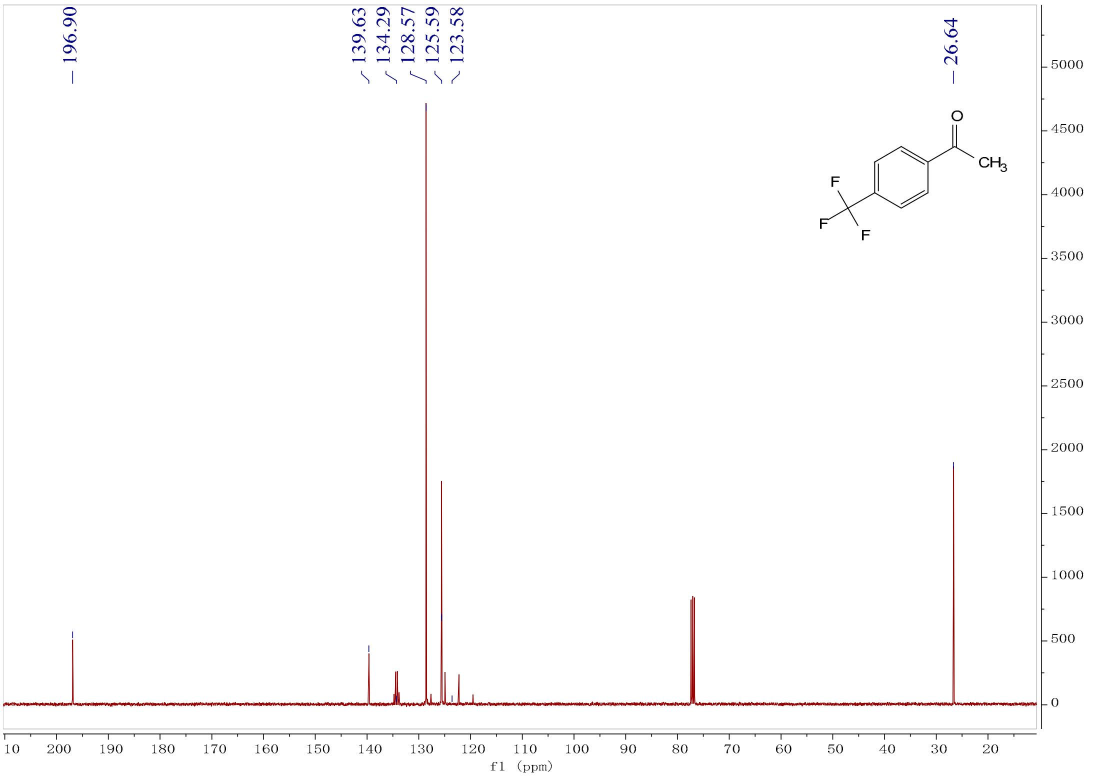

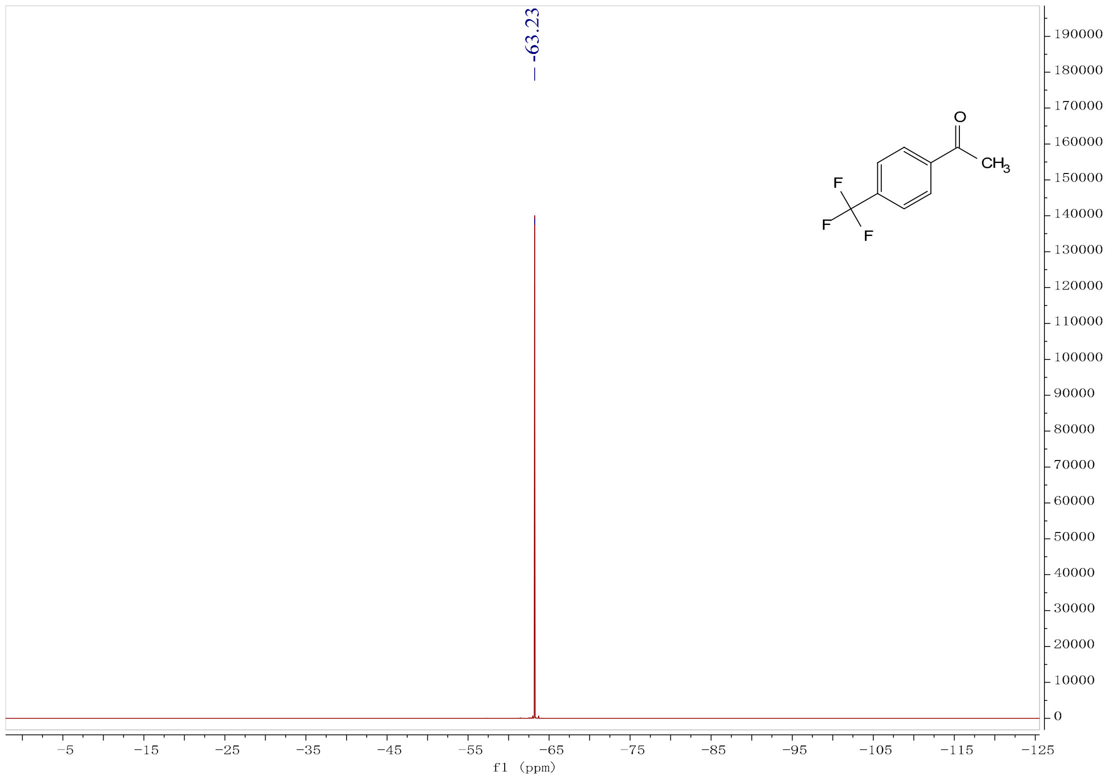

Figure S37. ^{1}H **(400 MHz),**^{13}C **{1H} (101 MHz) and** ^{19}F **{1H} (376 MHz) NMR spectra of 33c in** $CDCl_3$**.**

3,4–Dihydronaphthalen–1(2H)–one (34c)

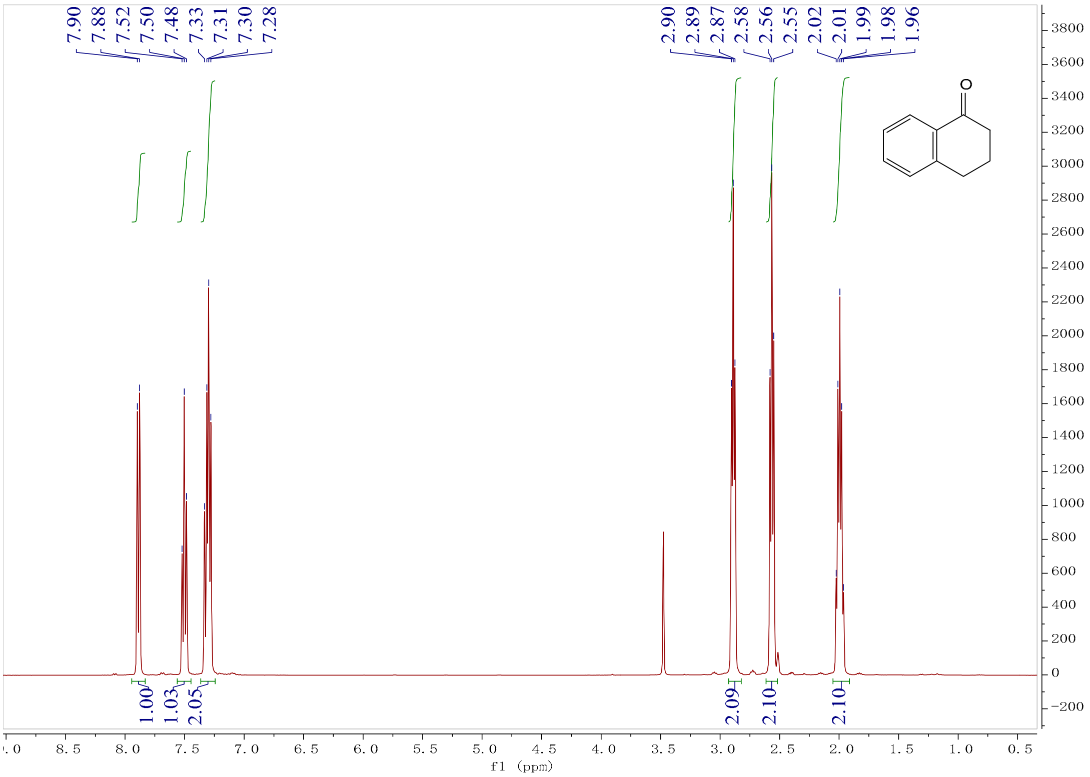

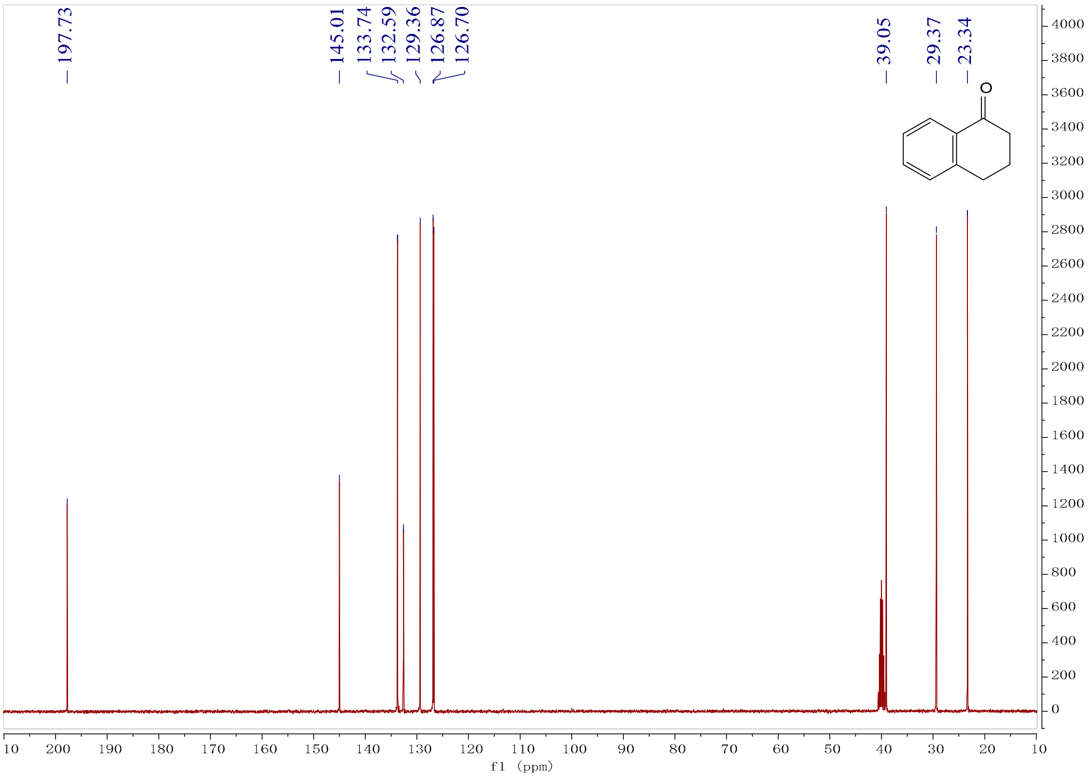

Figure S38. ^{1}H (400 MHz) and ^{13}C {1H} (101 MHz) NMR spectra of 34c in DMSO–*d*$_6$.

Benzophenone (35c)

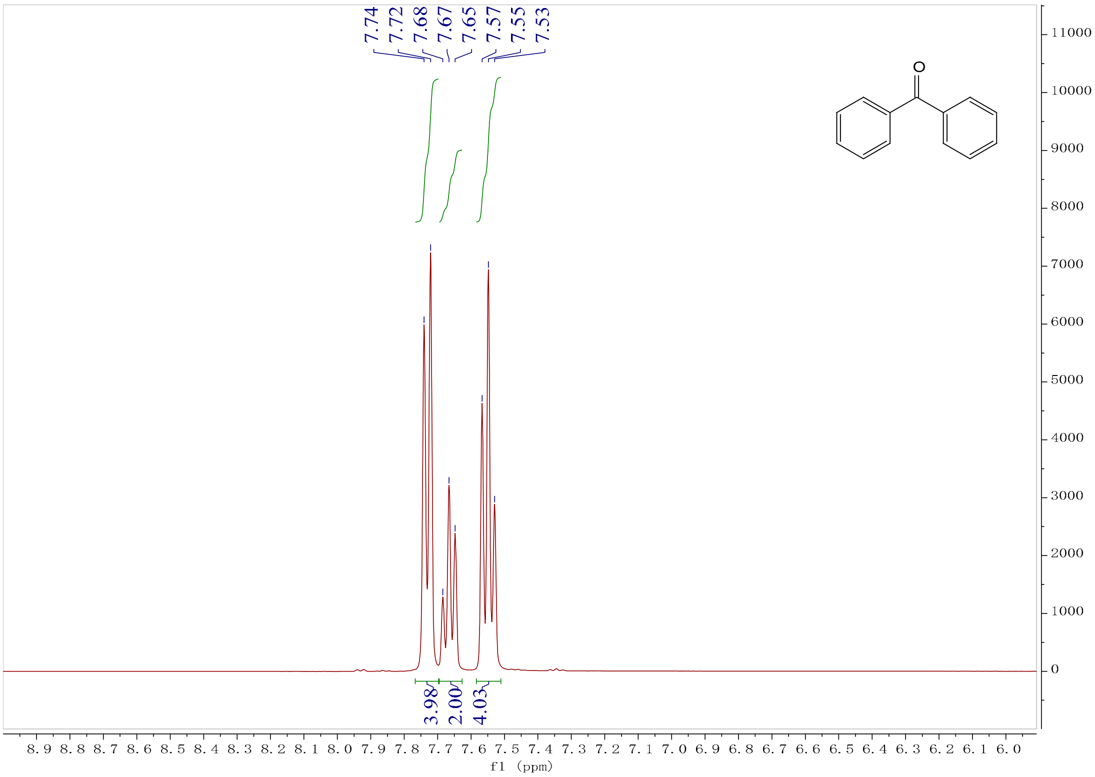

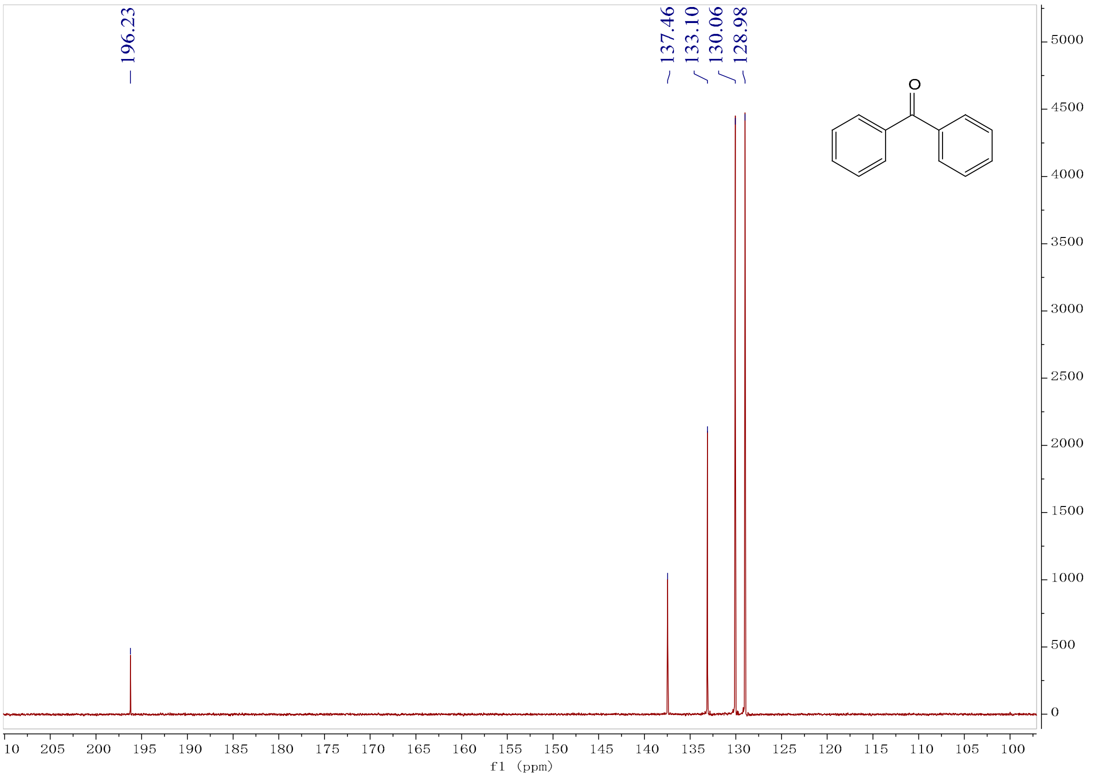

Figure S39. ^{1}H (400 MHz) and ^{13}C {1H} (101 MHz) NMR spectra of 35c in $CDCl_3$.

Cyclohexyl(phenyl)methanone (36c)

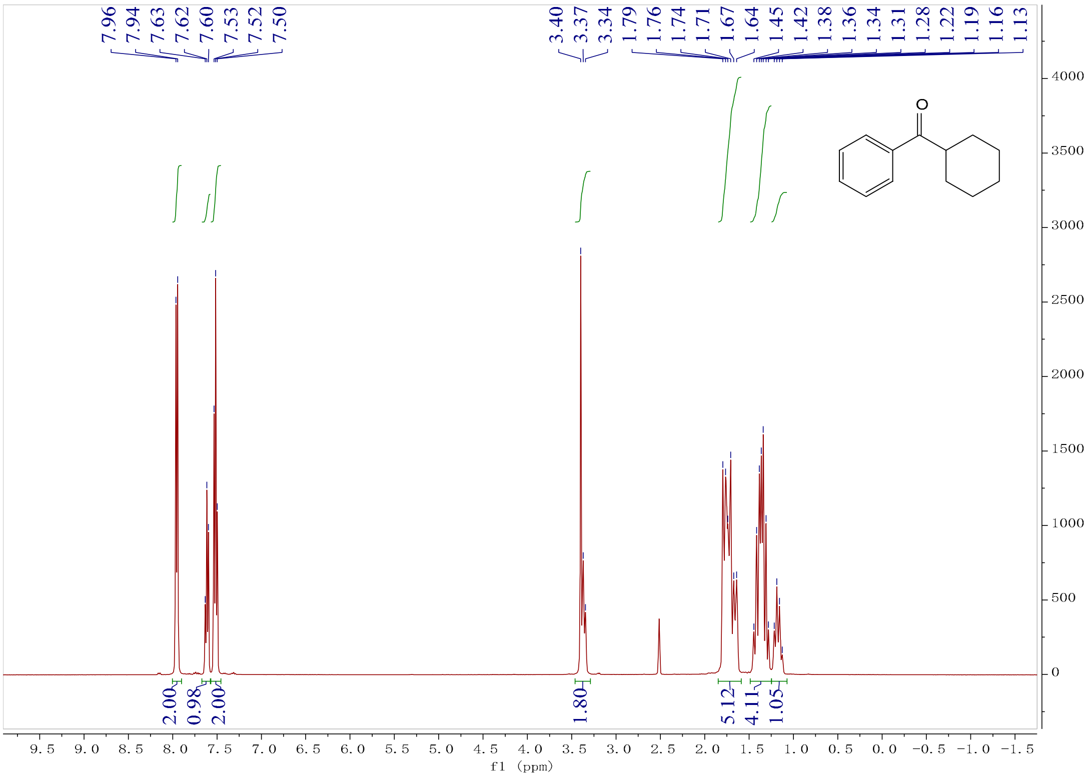

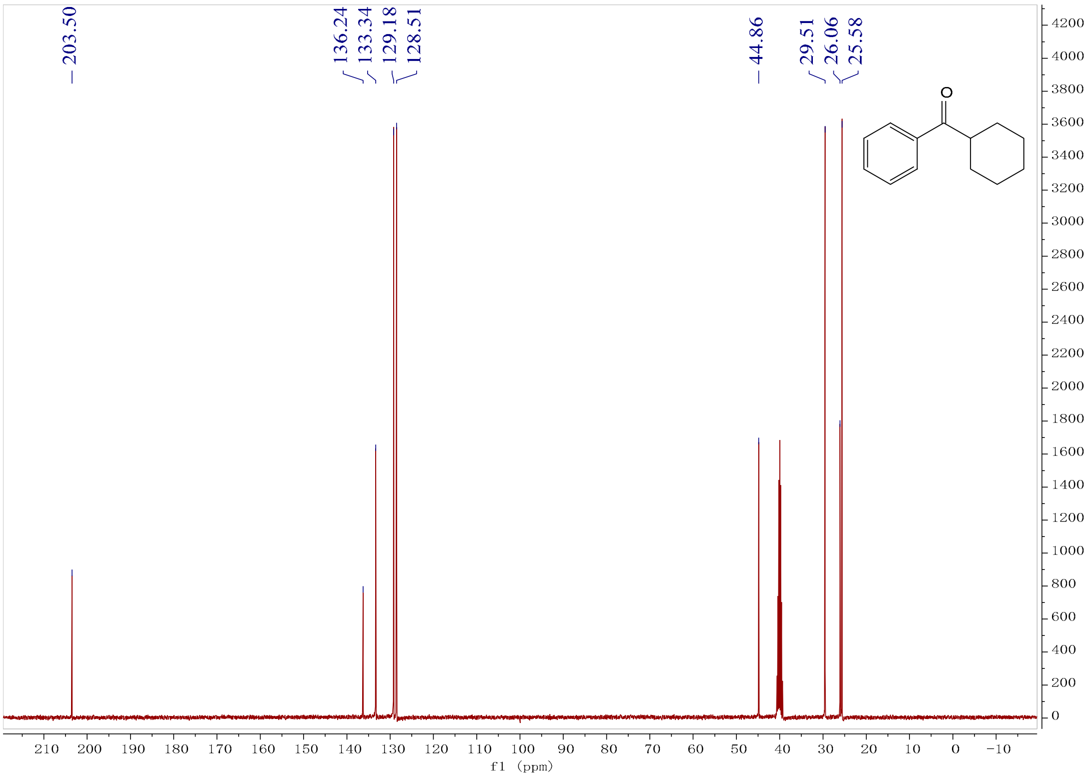

Figure S40. ^{1}H (400 MHz) and ^{13}C {1H} (101 MHz) NMR spectra of 36c in DMSO–d_6.

2–Acetylthiophene (37c)

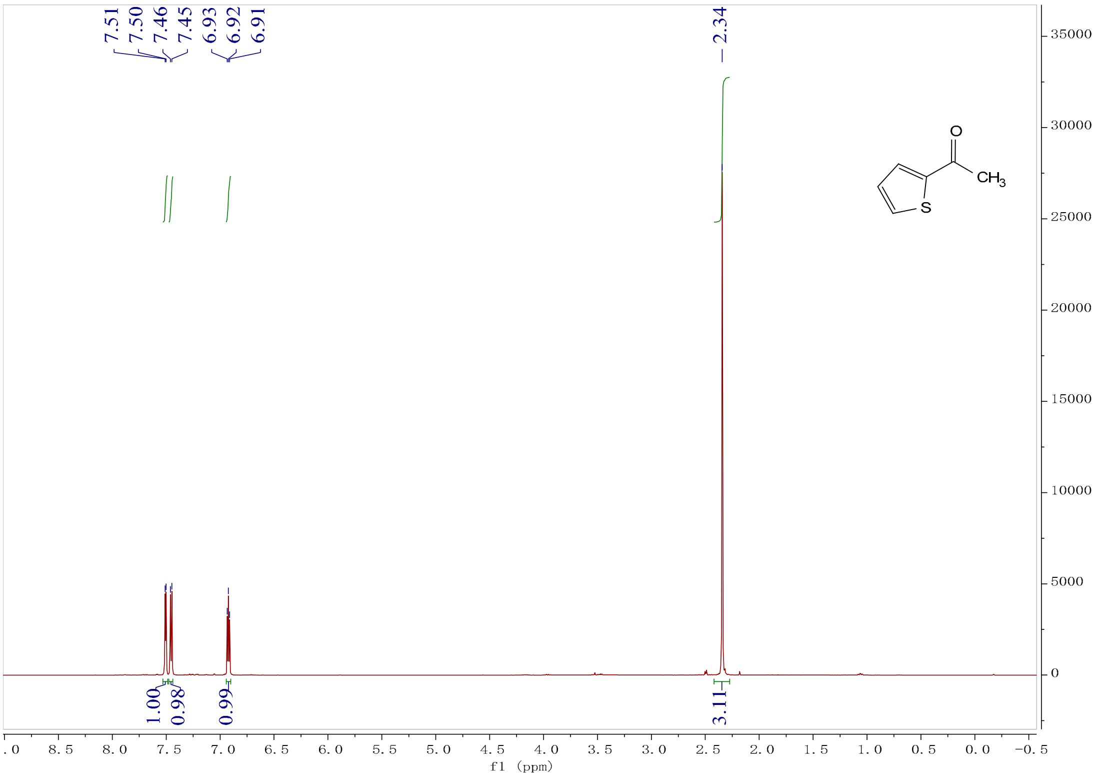

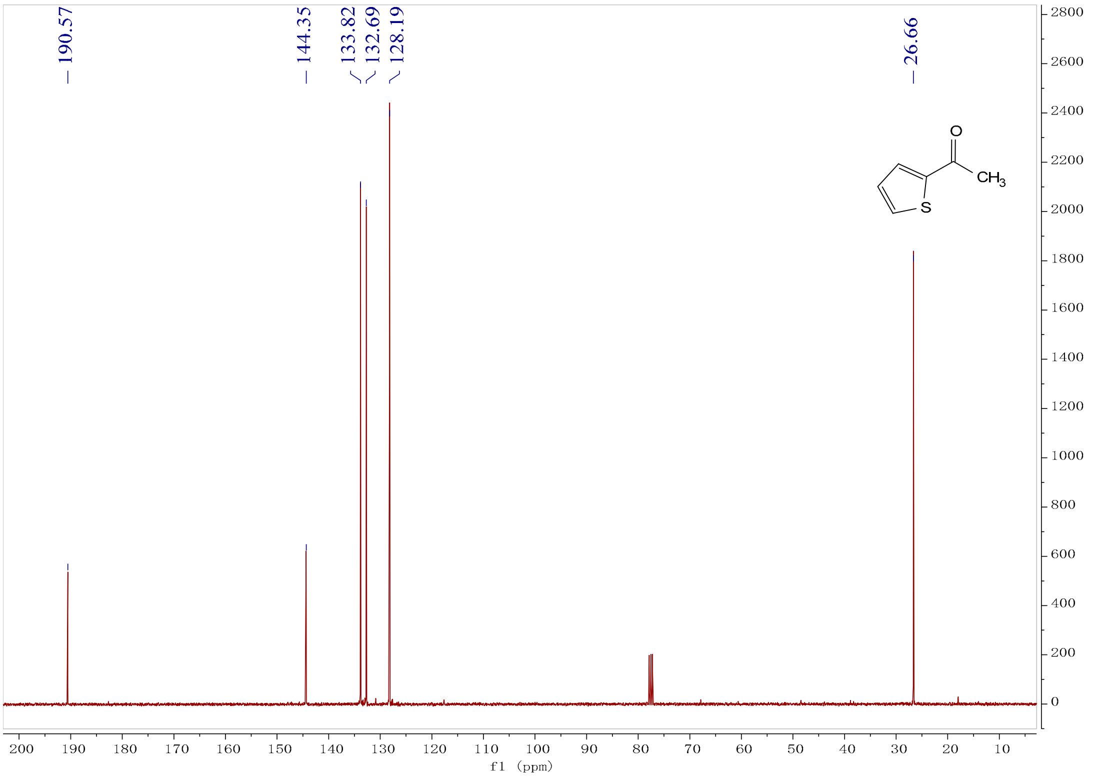

Figure S41. **^{1}H (400 MHz) and ^{13}C {1H} (101 MHz) NMR spectra of 37c in CDCl$_3$.**

Phenyl(thiophen–2–yl)methanone (38c)

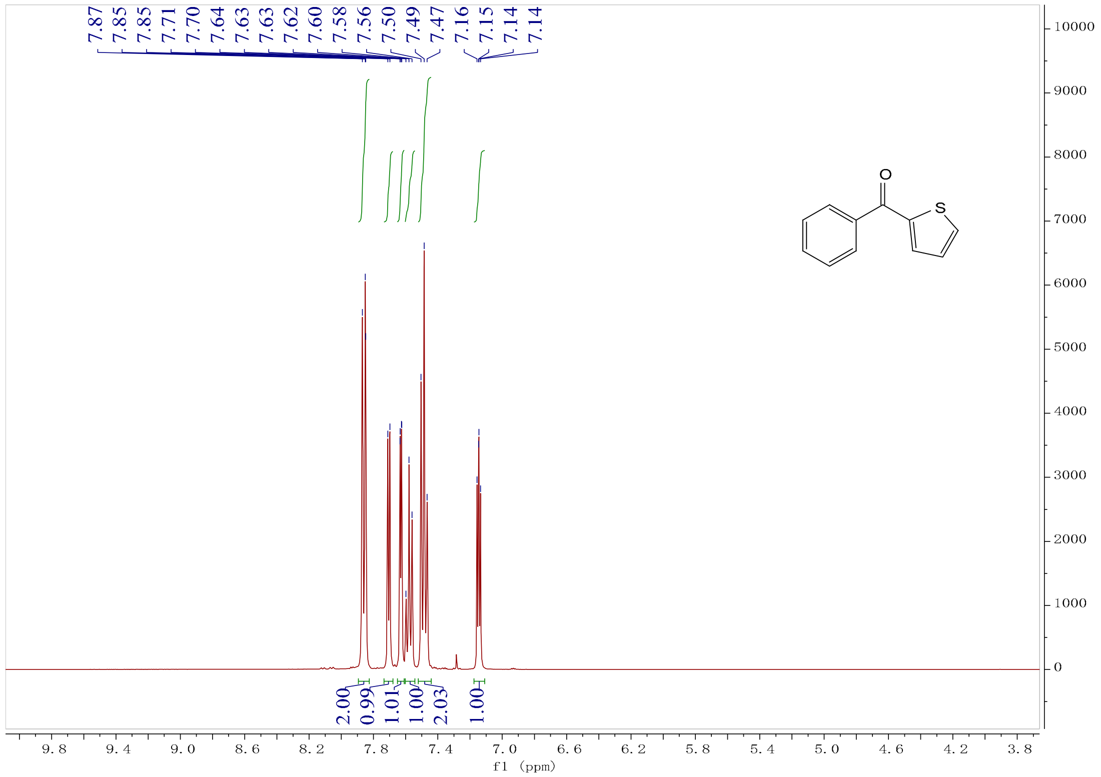

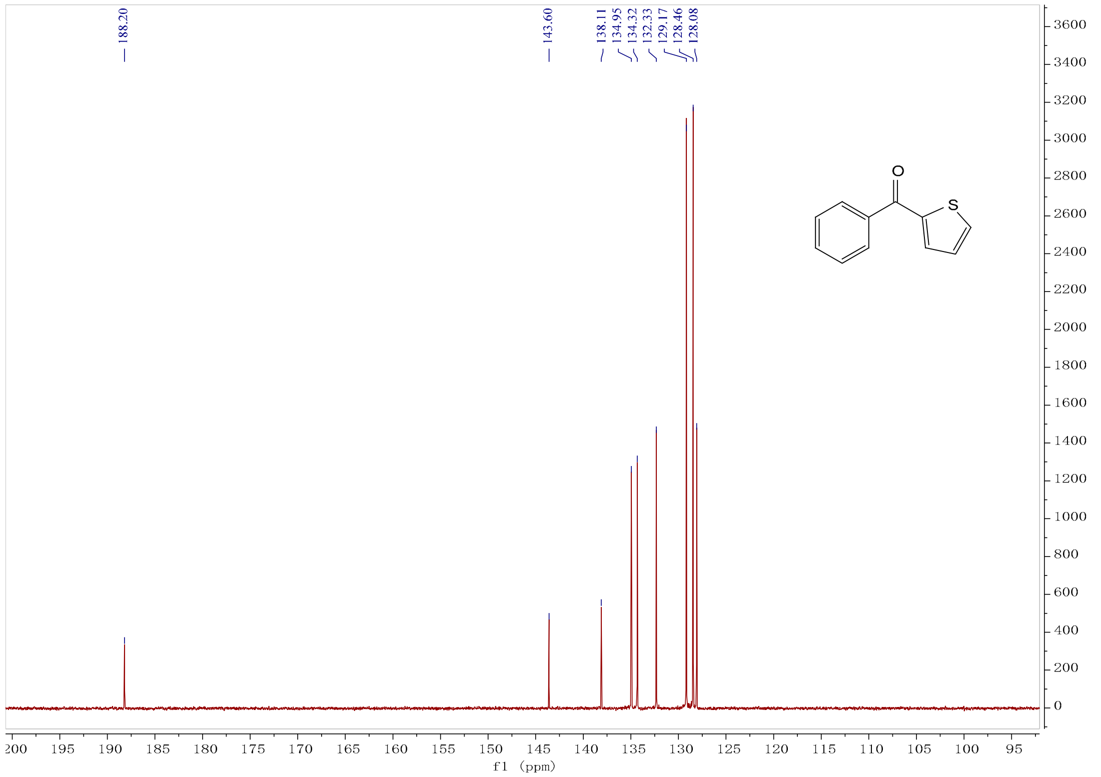

Figure S42. ^{1}H (400 MHz) and ^{13}C {1H} (101 MHz) NMR spectra of 38c in CDCl3.
